# Multiscale Modeling of Cardiovascular Function Predicts That the End-Systolic Pressure Volume Relationship Can Be Targeted via Multiple Therapeutic Strategies

**DOI:** 10.3389/fphys.2020.01043

**Published:** 2020-08-19

**Authors:** Kenneth S. Campbell, Brianna Sierra Chrisman, Stuart G. Campbell

**Affiliations:** ^1^Division of Cardiovascular Medicine, Department of Physiology, University of Kentucky, Lexington, KY, United States; ^2^Department of Biomedical Engineering, Yale University, New Haven, CT, United States

**Keywords:** cardiac function, computer modeling, Frank-Starling, multiscale modeling, ventricular function

## Abstract

Most patients who develop heart failure are unable to elevate their cardiac output on demand due to impaired contractility and/or reduced ventricular filling. Despite decades of research, few effective therapies for heart failure have been developed. In part, this may reflect the difficulty of predicting how perturbations to molecular-level mechanisms that are induced by drugs will scale up to modulate system-level properties such as blood pressure. Computer modeling might help with this process and thereby accelerate the development of better therapies for heart failure. This manuscript presents a new multiscale model that uses a single contractile element to drive an idealized ventricle that pumps blood around a closed circulation. The contractile element was formed by linking an existing model of dynamically coupled myofilaments with a well-established model of myocyte electrophysiology. The resulting framework spans from molecular-level events (including opening of ion channels and transitions between different myosin states) to properties such as ejection fraction that can be measured in patients. Initial calculations showed that the model reproduces many aspects of normal cardiovascular physiology including, for example, pressure-volume loops. Subsequent sensitivity tests then quantified how each model parameter influenced a range of system level properties. The first key finding was that the End Systolic Pressure Volume Relationship, a classic index of cardiac contractility, was ∼50% more sensitive to parameter changes than any other system-level property. The second important result was that parameters that primarily affect ventricular filling, such as passive stiffness and Ca^2+^ reuptake via sarco/endoplasmic reticulum Ca^2+^-ATPase (SERCA), also have a major impact on systolic properties including stroke work, myosin ATPase, and maximum ventricular pressure. These results reinforce the impact of diastolic function on ventricular performance and identify the End Systolic Pressure Volume Relationship as a particularly sensitive system-level property that can be targeted using multiple therapeutic strategies.

## Introduction

Diseases caused by reduced or dysregulated contractile function are a major clinical problem. About half of the 6 million Americans who have heart failure, for example, exhibit depressed contractile function (Benjamin et al., 2018). Another 700,000 Americans have inherited genetic mutations that have been linked to myopathies (Watkins et al., 2011; Houston and Stevens, 2014). Treatment options for most of these patients remain limited. For example, the clinical guidelines for heart failure (Writing Committee et al., 2013) recommend standardized therapies (primarily β-blockers and ACE inhibitors) that were developed > 30 years ago and produce a 5-year survival rate of only 50% (Benjamin et al., 2018). Given these facts, there is a pressing need to leverage the field’s ever-increasing knowledge of molecular and cellular-level processes to enhance clinical care.

Multiscaled computer modeling could accelerate this process. Indeed, a recent paper (Campbell et al., 2019) outlined a potential approach that creates patient-specific computer models that integrate genomic, proteomic, imaging, and functional data and then runs the models forward in time to predict how each patient will respond to possible therapeutic interventions. The authors went on to describe a moonshot goal of running a clinical trial to test whether implementing the model-optimized therapy helps patients more than the current standard of care. While this sort of endeavor still seems some way in the future, it captures the possible long-term impact of patient-focused modeling.

The current work describes one step in the development of multiscale models of cardiovascular function and builds on extensive prior work by many other groups including but not restricted to papers by Negroni and Lascano (1996), Shim et al. (2004), and Pironet et al. (2013). Specifically, the framework described here was constructed by linking the MyoSim model of dynamically-coupled myofilaments (Campbell et al., 2018) with the sophisticated model of myocyte electrophysiology developed by ten Tusscher et al. (2004). This created a contractile system that was based on molecular-level events (including opening of ion channels and transitions between different myosin states) that could be manipulated via numerical parameters. To the authors’ knowledge, the current model is the first to simulate blood circulation using a myofilament system that incorporates transitions between the OFF and ON states of myosin (Irving, 2017). Since these transitions contribute to length-dependent Ca^2+^-activation (Ait-Mou et al., 2016; Kampourakis et al., 2016; Zhang et al., 2017), the current model can help to improve understanding of ventricular function.

Organ-level function was simulated using the technique described by Shin et al. (Shim et al., 2004) and Campbell et al. (2008) which approximates the left ventricle as a hemisphere. In this approach, the volume of the ventricle is related to the length of a contractile element embedded circumferentially in the mid-transmural wall, while the chamber pressure is deduced from the stress in the contractile element via Laplaces’s law. The circulatory system was modeled using zero-dimensional (lumped parameter) compartments (Shi et al., 2011) representing the aorta, arteries, arterioles, capillaries, and veins. Flows between the different compartments were defined by Ohm’s law with one-way valves controlling the movement of blood into and out of the ventricle.

The model spans from molecular-level events to system-level properties but remains simple enough to run on a laptop. The code is open-source and available for free and unrestricted download. The results described in the following pages include sensitivity analyses that demonstrate how modulation of cell and molecular-level processes scale up to impact system-level properties. Some metrics (particularly the End Systolic Pressure Volume Relationship, ESPVR) were predicted to be particularly sensitive to molecular-level interventions while others (for example, stroke volume) were harder to modulate. These types of insights may prove useful as scientists try to translate their research toward improved clinical care.

## Materials and Methods

### Electrophysiological Model

Ten Tusscher et al.’s model of the electrophysiology of a mid-myocardial human myocyte (ten Tusscher et al., 2004) was downloaded as Python source code from CellML.org (Lloyd et al., 2008). The model was based on nine channels, four pumps, and two exchangers and has 17 state variables and 46 numerical parameters. Most of the parameters were kept fixed at the published values but the code was modified to allow adjustments to the following parameters: Ca V_leak_, Ca V_max_up_, g_CaL_ (all related to a calcium dynamics), g_to_ (transient outward current), g_Kr_ (rapid time-dependent potassium current), and g_Ks_ (slow time-dependent potassium current).

When paced at 1 Hz with 3 ms pulses of stimulus current (−52 pA pF^–1^), the base model took several hundred heart-beats to reach steady-state. The simulations presented in this work were initiated using the steady-state solution which was obtained by pre-calculating 1000 consecutive heart-beats.

### MyoSim

The mechanical properties of dynamically-coupled myofilaments were simulated using the MyoSim framework (Campbell, 2014). As shown in Supplementary Figure S1, binding sites on the thin filaments were activated by Ca^2+^. Cross-bridges transitioned between an OFF (also called super-relaxed, or interacting heads motif) state (Alamo et al., 2008; Hooijman et al., 2011; Nag et al., 2017), an ON state (that could attach to actin), and a single bound force-generating state. The bound heads contributed to cooperative activation of the thin filament. The rate of the OFF to ON transition increased linearly with force. As recently described by Campbell et al. (2018), this allows the model to exhibit length-dependent activation and reproduce the timing and magnitude of isometric twitch contractions measured at different sarcomere lengths (Campbell, 2011).

Additional details relating to the MyoSim model are provided in Supplementary Information.

### Ventricle

The ventricle was idealized as a single hemisphere characterized by a chamber volume V_ventricle_, a slack volume V_slack_ (when the passive stress in the MyoSim model was zero), a wall volume W_volume_, and an internal radius r. The wall thickness W_thickness_ was calculated as W_volume_ / (2 π r^2^) with r defined as:

(1) $$r\;=\;{\left(\frac{3\,V_{v\,e\,n\,t\,r\,i\,c\,l\,e}}{2\,\pi }\right)}^{1\,/\,3}$$

The pressure P_ventricle_ inside the ventricle was deduced from Laplace’s law as:

(2) $$P_{v\,e\,n\,t\,r\,i\,c\,l\,e}\;=\;\frac{2\,S\,W_{t\,h\,i\,c\,k\,n\,e\,s\,s}}{r}$$

where S was the wall stress calculated by the MyoSim model.

### Circulation

The systemic circulation was modeled using zero-dimensional compartments representing the aorta, arteries, arterioles, capillaries, and veins. Each compartment had a compliance C_x_ and a resistance R_x_ where x indicated the specific compartment. The pressure P_x_ in each compartment was calculated as P_x_ = V_x_/C_x_ where V_x_ is the compartment volume.

Ohm’s law was used to calculate the blood flow into and out of each compartment. The relevant formulae are included as Eqs (S11) and (S12) in Supplementary Information.

### Model Parameters

Base values for the parameters used in the simulations are shown in Supplementary Table S1. In contrast to many of the authors’ prior publications, no attempt was made to constrain the parameters using new experimental data. Instead, parameters were set to plausible values based on prior experience and typical values for a human. For example, the total blood volume V_total_ was fixed at 5 liters.

### Computer Code

Simulations were performed using code written in Python with non-trivial calculations performed using functions from the Numpy (van der Walt et al., 2011) and Scipy (Jones et al., 2001) libraries. Source code and documentation are available online at https://campbell-muscle-lab.github.io/PyMyoVent/. Simulations implemented with 1 ms time-steps ran in near real-time on a typical PC core. The electrophysiology model was the slowest part of the algorithm.

## Results

**FIGURE 1:**
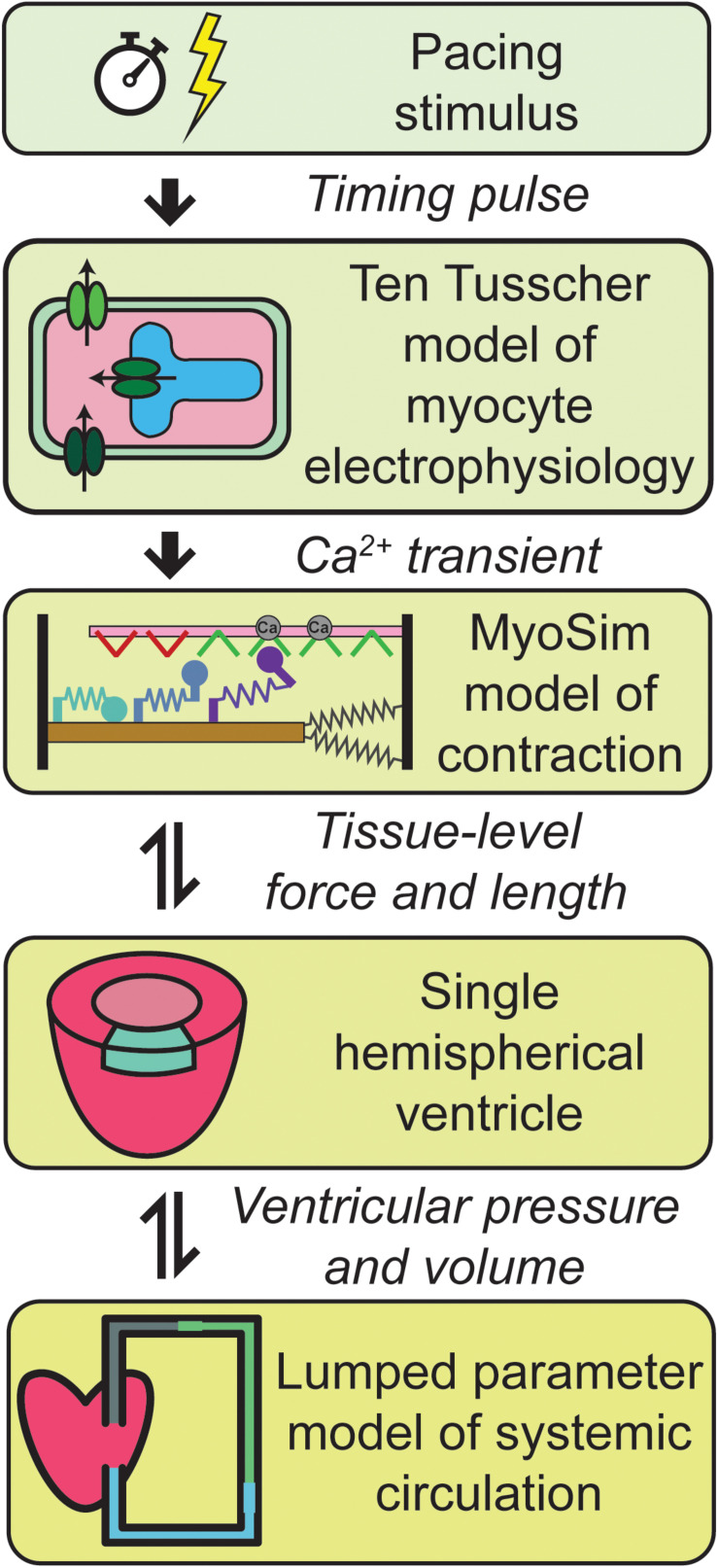
Overview of the multiscale model of cardiovascular physiology. Ca^2+^ transients simulated using a well-established model of the electrophysiology of a human cell (ten Tusscher et al., 2004) drove a contractile model implemented using MyoSim (Campbell, 2014; Campbell et al., 2018). The pressure and volume in the hemispherical ventricle were calculated from the contractile element’s force per unit area and length. The ventricle pumped blood through a systemic circulation that was mimicked using zero dimensional compartments. Additional details are provided in section “Materials and Methods.”

Figure 1 summarizes the multiscale model of the human cardiovascular system that was developed for this work. Heart-beats were initiated at 1 Hz by applying a pacing stimulus to a model published by ten Tusscher et al. (2004) that simulates the electrophysiology of a human mid-myocardial cell. At each time-step, the free intracellular Ca^2+^ concentration predicted by the electrophysiological model was passed to a MyoSim model of sarcomere level-contraction (Campbell, 2014; Campbell et al., 2018). The contraction model was coupled to a ventricle that was idealized as a hemisphere (Campbell et al., 2008). This approximation allowed the pressure and volume in the ventricle to be calculated from the contractile element’s force per unit area and length. The ventricle was in turn coupled to a circulation that was mimicked using lumped parameters and valves that controlled the flow of blood into and out of the heart.

Additional details about each level of the model are provided in section “Materials and Methods.”

### Base Simulation

**FIGURE 2:**
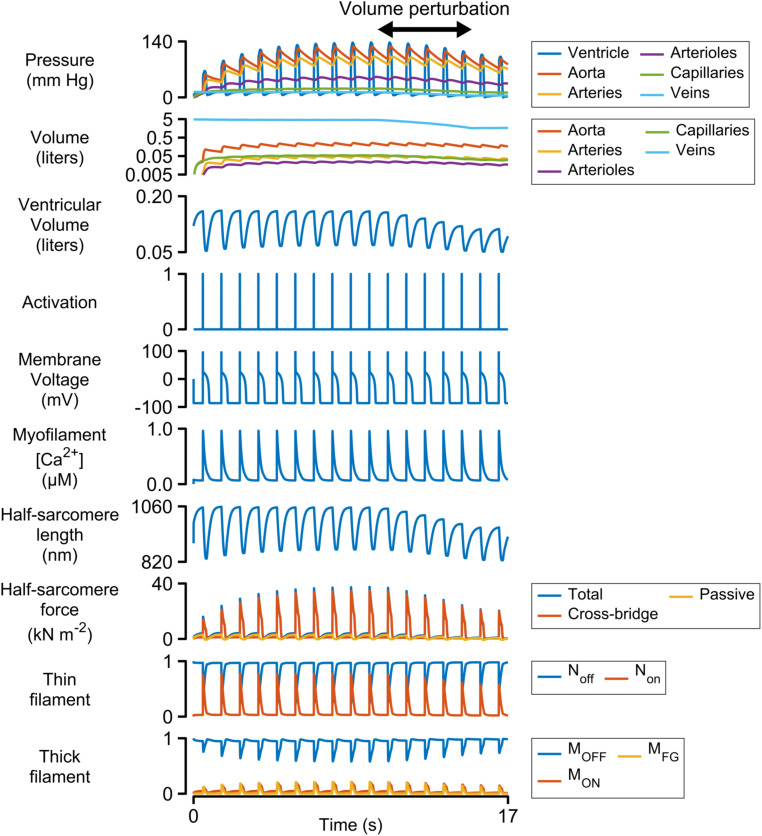
Multiscale simulation of 17 heart-beats. The simulation took about seven beats to reach steady-state as blood was pumped through the systemic circulation. The blood volume was reduced by 60% between the 10th and 15th beats to determine how the ventricle responded to reduced pre-load. The panel showing the volumes of the circulatory compartments is plotted on a log scale. All other panels are plotted with linear scales.

Figure 2 shows a simulation of 17 heart beats performed using the base parameters listed in Supplementary Table S1. The calculations were started with the ventricle filled to 150% of its slack volume and the remainder of the blood in the venous compartment. Heart beats were initiated once per second by activating a 3 ms pulse of stimulus current (−52 pA pF^–1^) in the electrophysiological model.

Each Ca^2+^ transient activated thin filaments in the contractile model. This allowed myosin heads to transition into the force-generating state, thereby raising wall stress and ventricular pressure (Eq. 2). Once the ventricular pressure exceeded aortic pressure, blood was pumped into the aorta, and subsequently through the other circulatory components, with inter-compartmental flow rates defined by Ohm’s law (Eq. S12 in Supplementary Information). The ventricle relaxed when the intracellular Ca^2+^ concentration declined and ventricular pressure dropped below that in the aorta. Once ventricular pressure fell below venous pressure, blood flowed back into the heart, re-stretching the ventricle and extending the contractile element.

The simulation evolved toward a steady-state as blood moved through the circulation to fill the arterial, arteriolar, capillary, and venous compartments. This required approximately seven beats with the base parameters. A volume perturbation was imposed during the second half of the simulation (Figure 2, double-headed arrow) to determine how the ventricle responded to reduced pre-load (ventricular filling).

**FIGURE 3:**
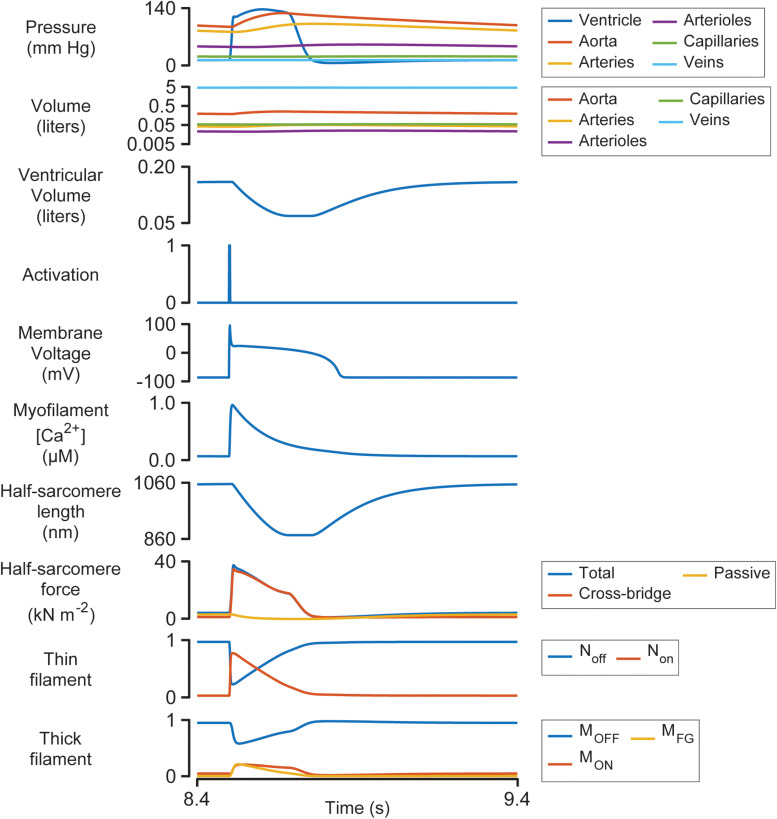
Multiscale simulation of a heart-beat at steady-state. The figure shows the 8th beat from Figure 2 on an expanded time-scale.

Figure 3 illustrates the steady-state behavior of the system on an expanded time-scale. The heart beat was initiated by the activation pulse which triggered an action potential and an intracellular Ca^2+^ transient. N_on_ rose sharply as the thin filament was activated by the Ca^2+^ signal (see Supplementary Figure S1 and additional details in Supplementary Information). This allowed some of the myosin heads in the M_ON_ state to transition into the M_FG_ state. The force developed by the attached cross-bridges accelerated the J_1_ transition and pulled additional heads from M_OFF_ to M_ON_ and subsequently to M_FG_ via a positive-feedback loop that was modulated by thin filament cooperativity and force-dependent recruitment (Campbell et al., 2018). Ventricular pressure scaled with the force generated by the cross-bridges and increased quickly while the chamber remained isovolumic.

Once ventricular pressure exceeded aortic pressure, blood was pumped into the aorta, and the contractile element shortened as the ventricle started to contract. The shortening reduced the force generated by attached cross-bridges and the M_OFF_ state started to repopulate. Although half-sarcomere force decreased by ∼50% during ejection, ventricular pressure remained elevated due to the diminishing internal radius of the chamber (Eq. 2).

The final stages of relaxation progressed under isometric conditions as the thin filament deactivated and myosin heads that detached from the M_FG_ state were no longer able to re-bind to available sites.

### End Systolic Pressure Volume Relationship

**FIGURE 4:**
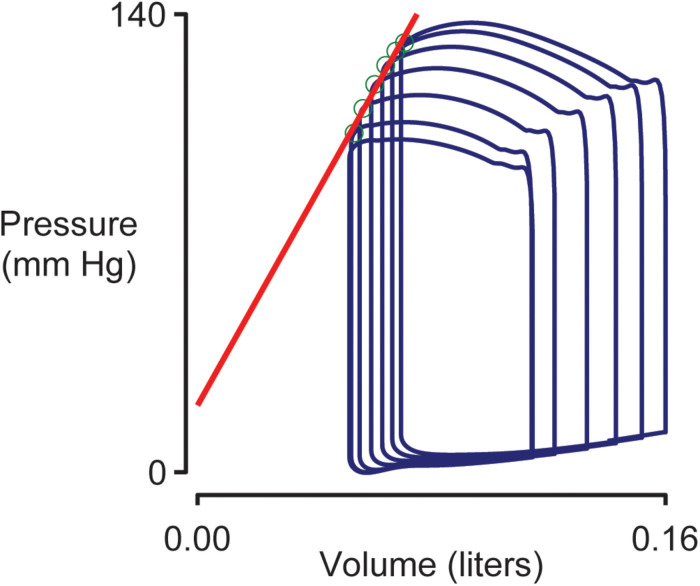
Simulated pressure volume loops and the End Systolic Pressure Volume Relationship (ESPVR). Ventricular pressure plotted against ventricular volume for beats 10–15 of the simulation shown in Figure 2. The ESPVR was calculated as the slope of a regression line (shown in red) fitted to the top-left corner (green circles) of each pressure-volume loop.

Figure 4 shows pressure-volume loops (ventricular pressure plotted against ventricular volume) for beats 10–15 of the simulation shown in Figure 2. Blood was being removed from the venous compartment during this phase of the simulation so the pressure-volume loops showed a progressive trend toward lower pressure and lower volume. The End Systolic Pressure Volume Relationship (ESPVR, an index of cardiac contractility) was calculated as the slope of a regression line fitted to the top left corner of each loop. In this example, the ESPVR was 1170 mm Hg liter^–1^.

### Sensitivity Analysis

**FIGURE 5:**
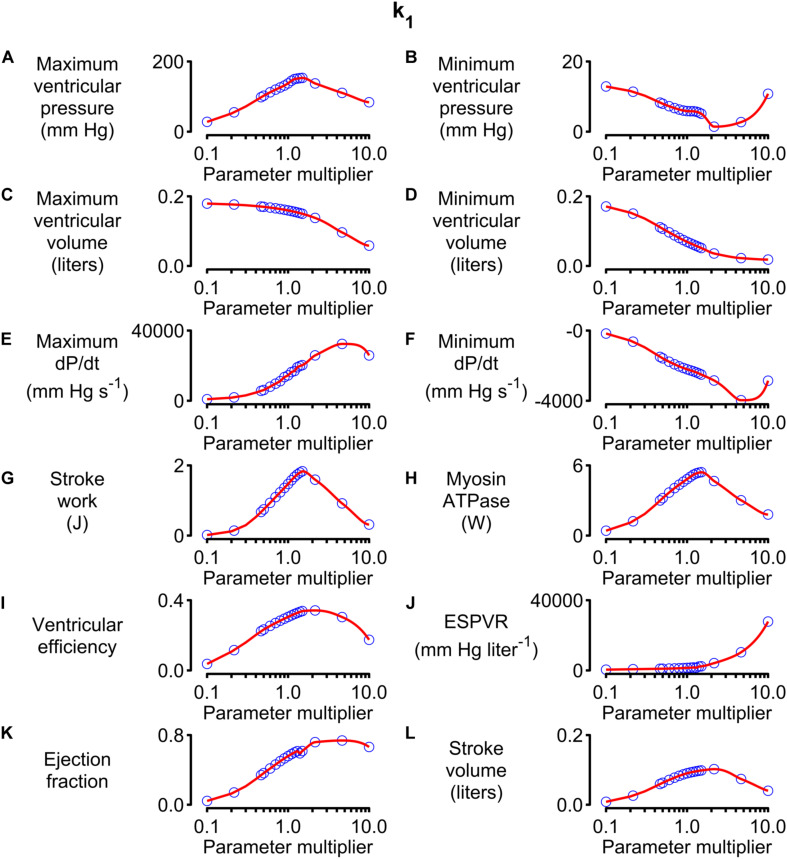
Effects of changing k_1_ on system-level properties. **(A–L)** Show values (blue circles) for 12 system-level properties (for example, maximum ventricular pressure) predicted for values of k_1_ ranging from 0.1 to 10 times the value shown in Supplementary Table S1. The red lines show the best-fit of a 5th order polynomial to the simulated data. The relative sensitivity for each property was defined as the slope of the polynomial at the base parameter value (that is, when the multiplier was 1) divided by the corresponding y value. The division normalized the slope to facilitate comparisons between different properties.

Figure 5 shows how 12 system-level properties varied as the k_1_ parameter was adjusted from 0.1 to 10 times the base value shown in Supplementary Table S1. Steady-state beats and single-beat estimates of the ESPVR for three of the k_1_ parameter values are shown in Supplementary Figures S2, S3. Although the limits are somewhat arbitrary, the 0.1 to 10x range is probably large enough to encompass the functional effects that could be achieved through pharmaceutical modulation of the molecular transition. The red lines in each panel show the best-fit of a 5th order polynomial to the simulated data. The slope of each polynomial was evaluated at the base parameter value and normalized to the corresponding y value to produce relative sensitivity metrics that defined how each system-level property varied with small adjustments in k_1_. For example, the relative sensitivity metric was +0.49 for maximum ventricular pressure (because it increased with small increases in k_1_ near the base value, Figure 5A) but −0.17 for minimum ventricular pressure (because this property decreased as k_1_ was increased, Figure 5B).

Note that the sensitivity metric only quantifies the effect of small changes in the model parameter. Larger changes often produced more complex effects that, in many cases, resulted in non-physiological behavior. For example, maximum ventricular pressure initially increased as k_1_ was elevated above its base value because the OFF/ON thick filament equilibrium was shifted toward the ON state. This corresponds to increased cardiac contractility. However, if k_1_ was increased by more than a factor of ∼3, the active contraction became so strong that the ventricle ejected most of its contents into the circulation and shrank down to a small volume. Cardiac output thus decreased to a non-physiological extent if contractility increased beyond a certain point. This effect is illustrated in Supplementary Figure S4.

Calculations summarizing the effects of adjusting other model parameters are shown in Supplementary Figures S5–S34. Note that each parameter was scaled from 0.1 to 10 times its base value in order to maintain a fixed proportional change. Non-monotonic relationships between model parameters and system-level properties (similar to the relationship between k_1_ and stroke volume described above) were observed frequently.

Alternative strategies, including spline interpolation, could have been used to deduce sensitivity metrics but 5th order polynomials were chosen because they produced satisfactory fits to the simulated data and the calculations were straightforward to implement.

**FIGURE 6:**
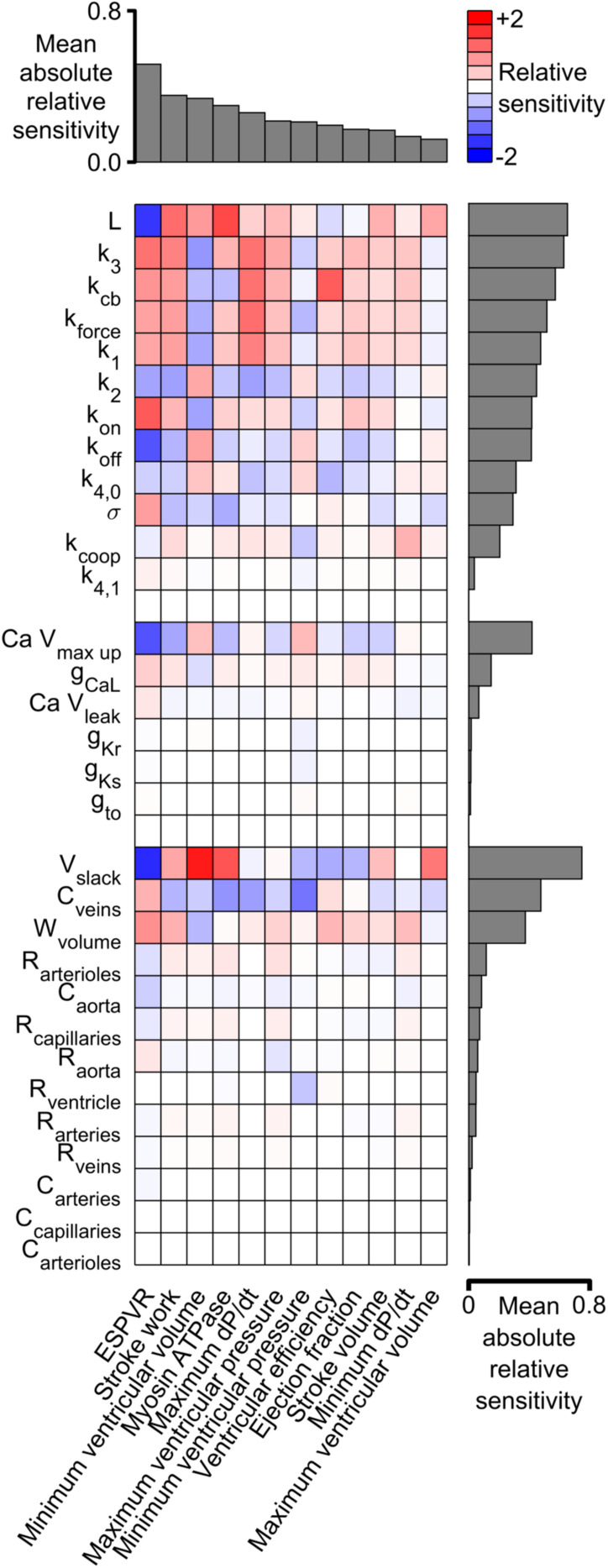
Relative sensitivities for each combination of system-level property and model parameter. Red colors show property / parameter combinations that were positively correlated for small changes in the base parameter value. For example, stroke work, minimum ventricular pressure, and myosin ATPase all increased as L became larger (red boxes in columns 2–4 of the top row and Supplementary Figure S5). Blue colors show negative correlations. For example, ESPVR decreased as L became larger (blue box in column 1 of top row). The gray bars above the matrix show the mean of the absolute relative sensitivity values for each system-level property. The bars on the right show the corresponding means for each model parameter. The model parameters are clustered in three groups corresponding to the sarcomeric, electrophysiological, and circulatory levels of the model.

Figure 6 shows the sensitivity metrics for every combination of system-level property and model parameter as a heat-map. The first key finding from this analysis is shown by the gray bars above the matrix. These are arranged in order of size and represent the mean value of the absolute relative sensitivities for each system-level property (The absolute term prevents positive and negative relative sensitivities from canceling out). The highest bar, and thus the most sensitive property, was the ESPVR which had a mean absolute relative sensitivity of 0.52. The next most sensitive property was stroke work with a mean value of 0.35. One implication is that perturbing a randomly-chosen model parameter is likely to change the ESPVR at least 50% more than any other system-level property.

A second key finding is that both systolic and diastolic properties were sensitive to ventricular filling. The gray bars to the right of the heat-map show the mean of the absolute sensitivities for each model parameter. The bars are separated into three groups corresponding to the sarcomeric, electrophysiological, and circulatory levels of the model. The most sensitive parameters in each group were L (the length constant for the passive tension curve, equation S9), Ca V_max up_ (a parameter that controls how quickly Ca^2+^ is pumped into the sarcoplasmic reticulum), and V_slack_ (the volume at which passive wall stress in the ventricle is zero). Although these parameters are most obviously related to diastolic function, the sensitivity metrics show that they also influenced systolic properties. For example, all three parameters had a strong impact on ESPVR, stroke work, and minimum ventricular volume. These findings reinforce the impact of diastolic function on ventricular performance and could be interpreted as a molecular-level correlate of the Frank-Starling mechanism.

## Discussion

This work presents a computer model that bridges from molecular-level mechanisms (for example, opening of ion channels) to system-level cardiovascular properties (for example, blood pressure) that are measured in patients. The approach helps narrow the wide gap that currently separates myocyte biophysics from clinical cardiology and might have the potential to help accelerate translational research. For example, Figure 6 illustrates how sensitivity analysis can be used to identify molecular-level interventions that have a particularly large impact on cardiovascular function. Similar calculations, perhaps based on more sophisticated models, might prove useful for *in silico* screens designed to identify potential therapeutic targets (Campbell et al., 2019).

### Alternative Strategies for Sensitivity Analysis

The sensitivity metrics used in this work quantified the relationship between individual molecular-level parameters and a suite of system-level properties. This approach was selected because many of the molecular parameters in the model can targeted *in vivo* using current and/or potential future pharmaceutical interventions. Other mathematical approaches are possible. For example, Sobel analysis is a variance-based sensitivity method that can quantify the effects of combinations of parameters (Sobol, 1993). It can therefore test for interactions between system inputs (Wenk, 2011).

Another option is to test how system-level function depends on chamber-level properties. For example, as shown in Eq. (S9), the passive wall stress at a given half-sarcomere length depends on three molecular-level parameters: σ (a scaling factor), L_slack_ (the half-sarcomere length at zero passive stress), and L (a parameter that defines the curvature of the passive length-tension relationship). Supplementary Figure S35 shows how system-level properties varied as σ and L were adjusted simultaneously so that passive stress at a given half-sarcomere length was held constant. Essentially, this presentation shows how the system is influenced by the non-linear stiffness of the ventricle. Accordingly, this type of analysis might be useful for scientists and clinicians who think primarily about chamber properties rather than biophysical parameters. It could also be extended to quantify the effects of association constants (for example, k_1_/k_2_, Eqs S3 and S4) rather than the individual parameters.

### Physiological Insights

Figure 6 showed that the End Systolic Pressure Volume Relationship (ESPVR) was ∼50% more sensitive to the model parameters than any other system-level property. Indeed, the absolute values of the ESPVR sensitivities exceeded 0.3 for 10 of the 12 sarcomere-level parameters. The sensitivities were positive for k_3_, k_cb_, k_force_, k_1_, k_on_, and σ, and negative for L, k_off_, and k_4,0_ (see Supplementary Information for additional details about the mechanisms controlled by each parameter). These are important findings because the ESPVR is a classic measure of cardiac contractility and frequently depressed in patients who have heart failure (Parmley, 1985).

One interpretation of these results is that contractility is reduced in heart failure precisely because the EPSVR is impacted by so many mechanisms. A subtle deficit in nearly any aspect of sarcomere-level function could compromise contractility, with the ESPVR’s high sensitivity to molecular-level function compounding the system-level problem.

An alternative, more optimistic, interpretation is that contractility can be rescued using multiple therapeutic strategies. Consider a hypothetical patient who develops heart failure because they have inherited a mutation that reduces the rate of cross-bridge binding (k_3_, Eq. S5 in Supplementary Information). This individual could potentially be treated with a small molecule, such as omecamtiv mecarbil, that accelerates the transition and reverses the original deficit (Malik et al., 2011; Swenson et al., 2017). If this approach failed, perhaps because the mutation also altered the drug’s binding pocket, Figure 6 suggests that alternative treatment strategies could include: enhancing force-dependent recruitment (k_force_), increasing titin-based stiffness (σ), reducing the thin filament off rate (k_off_) and/or slowing myosin detachment (k_4,0_). While the ultimate goal is, of course, to develop computer models that are specific to individual patients, even the simple analyses described here might help clinicians to weigh options in cases that are particularly challenging.

Additional physiological insights can be gained by viewing the relationships between sensitivity metrics. For example, the metrics for stroke work and stroke volume (equivalent to cardiac output in this work since heart rate was fixed) are correlated: parameter modifications that increased one, also increased the other. Unfortunately, the mean absolute relative sensitivity for stroke work was 0.35, almost double the corresponding value for stroke volume. This implies that it could be difficult to enhance cardiac output in patients without a greater than proportional change in metabolic cost. Again, this could be problematic for patients who have heart failure and who already struggling to maintain adequate circulation.

The parameter sweeps (Figure 5 and Supplementary Figures S5–S34) summarize large amounts of data but the results for L (Supplementary Figure S5) are particularly note-worthy. This parameter controls the curvature of the passive length tension relationship (Eq. S9) and had a particularly strong effect on most system-level properties. As noted earlier, the high mean absolute relative sensitivity of L reinforces the importance of diastolic function for ventricular performance. The additional point made here is that ventricular efficiency exhibited a clear optimum near L’s base parameter value (Supplementary Figure S5I). Ventricles with smaller values of L (stiffer chambers) or larger values (more compliant chambers) used greater amounts of ATP to perform a given amount of work.

The main sources of passive tension in myocardium are collagen, titin, microtubules, and intermediate filaments but titin is probably the most important component at physiological sarcomere lengths (Granzier and Irving, 1995). Titin’s stiffness can be modulated by multiple post-translational mechanisms (Hidalgo and Granzier, 2013) and the relative expression of its isoforms changes with disease (Nagueh et al., 2004). Perhaps the relationship between titin’s stiffness and ventricular efficiency is one of the driving forces underpinning these complex regulatory effects?

Another parameter that deserves comment is k_1_. As shown in Eq. (S3) in Supplementary Information, this parameter modulates the rate at which myosin heads transition from the OFF to the ON state. Since only heads in the ON state can attach to actin, k_1_ has a strong influence on contractility. This is evident from the parameter sensitivities shown in Figure 6 and the simulations shown in Supplementary Figures S2, S3. The last of these figures shows single-beat estimates of the ESPVR determined using the curve-fitting method described by Mirsky et al. (1987). These simulations suggest that the ESPVR increases by a factor of 2.65 as k_1_ is increased from 1 to 3 s^–1^. OFF / ON transitions in striated muscle are currently a very exciting area of research and are thought to be regulated by myosin-binding protein-C (McNamara et al., 2016) as well as being an important therapeutic target (Anderson et al., 2018). It may be useful to note that the MyoSim framework has also been used to investigate how phosphorylation of regulatory light chain accelerates the ON transition in cardiac trabeculae from rodents and thereby increases Ca^2+^ sensitivity (Kampourakis et al., 2016; Campbell et al., 2018). In the current simulations, this could be modeled as a phosphorylation dependent change in the k_1_ parameter.

The final physiological insight described here relates to stroke volume. The parameter sweeps show that this system-level property has a clear optimum for about 50% of the model parameters (including, for example, k_1_, Figure 5, and k_on_, Supplementary Figure S11). The optimum reflects the fact that weak contractions do not pump much blood while contractions that are excessively strong squeeze the ventricle dry (Supplementary Figure S4). Contractility seems to be a situation where you really can have too much of a good thing!

### Limitations

Despite its strengths, the current model has several limitations. These include: (1) the single ventricle framework, (2) the omission of ventricular geometry and torsional effects, (3) the absence of autonomic control, and (4) the one-way coupling of the electrophysiological and contractile modules. These issues are considered in more detail in the following paragraphs.

The single ventricle framework is probably the most obvious limitation of the current model. Human hearts have two ventricles, with the left chamber pumping blood through the systemic circulation to the right side of the heart, and the right ventricle pumping blood through the pulmonary circulation to complete the circuit. Averaged over time, the flows through the systemic and pulmonary systems must be equal. However, perturbations such as a change in posture or a Valsalva maneuver, produce short-term differences in left and right ventricular output. Similarly, clinical conditions that initially effect one ventricle (for example, pulmonary hypertension) can induce long-term remodeling in the other ventricle (Vonk Noordegraaf et al., 2017). It’s clearly impossible to study these types of effects with a single ventricle framework.

Lumens et al. (2009) developed the TriSeg model to overcome this issue. The approach developed by these authors is conceptually similar to the single ventricle framework presented here but uses a more sophisticated geometry. Specifically, the TriSeg model simulates a biventricular heart using three contractile elements, one in the left ventricular free wall, one in the right ventricular free wall, and one in the septum. This allows simulation of complex organ-level effects including interactions between the left and right ventricles and septal geometry dynamics. One disadvantage of Lumens et al.’s original implementation was that contractile forces were calculated using a heuristic approach, that is, from equations that linked myofiber strain and force but were not based on a molecular-level model.

More recent calculations using the TriSeg framework performed by Tewari et al. (2016) replaced the heuristic equations with a sophisticated cross-bridge model and added in an additional model of myocardial metabolism. However, Tewari et al.’s contractions were driven with experimentally-recorded Ca^2+^ transients rather than with an electrophysiological model as in this manuscript. This data-driven simulation approach is very useful because it eliminates potential uncertainties associated with the electrophysiological model. A drawback is that it makes it difficult to investigate how electrophysiological changes influence system-level behavior. For example, Tewari et al.’s model can’t predict how SERCA activity influences function (Supplementary Figure S16) because the simulations don’t include this level of molecular detail. An exciting next step would be to combine the strengths of the different frameworks and create a TriSeg model that integrates models of electrophysiology, metabolism, and contraction.

The second limitation relates to ventricular geometry. The current framework idealizes the ventricle as a thin-walled hemisphere. This is useful because it allows the ventricular volume and pressure to be calculated from the chamber’s circumference and wall-stress. However, the simplification completely neglects many important features. For example, the current framework cannot reproduce cardiac torsion, the complex twisting motion of the heart (Russel et al., 2009). Nor can the model incorporate transmural (Haynes et al., 2014) or potential base to apex variation (Sharma et al., 2003) in contractile properties and fiber alignment (Streeter et al., 1969). Since the ventricle is mimicked as a hemisphere, it is also impossible to simulate the patient-specific geometries required for personalized simulations. This may be particularly limiting for studies focused on hypertrophic cardiomyopathy. This clinical condition frequently reflects a sarcomeric gene mutation and is often associated with region-specific growth, most commonly involving the basal interventricular septum (Marian and Braunwald, 2017).

The best way to simulate regional and architectural effects is to implement a finite element model. This approach deconstructs the entire heart into small interconnected blocks (the elements), calculates the physical properties (stress and dimensions) of each element, and then uses applied mathematics to integrate the block-level data to infer global function (Guccione et al., 1995). Cardiac finite element modeling is a highly evolved field and multiple groups have used the technique to investigate complex behaviors including transmural variation (Wang et al., 2016), torsion (Bagnoli et al., 2011), and regional growth (Klepach et al., 2012; Lee et al., 2016). Although many cardiac finite element models are based on phenomenological contraction laws, two recent publications have used the MyoSim model to simulate the contractile properties of each element. (Zhang et al., 2018; Mann et al., 2020). The main disadvantage of the approach is that the calculations are very involved. As a result, finite element simulations are typically run on a dedicated computer cluster and typically require hours to complete. This differentiates the technique from the current model which is simple enough to run in near real-time on a laptop.

One of the disadvantages of using the current model to screen potential therapeutic strategies is that the calculations assume that the heart is otherwise unperturbed and continues to beat once per second. A real heart would obviously be subject to autonomic control. Although beyond the scope of this work, it will be possible to reproduce autonomic control in future research by implementing a virtual baroreceptor (Kosinski et al., 2018). This could be done by monitoring the arterial blood pressure predicted by the model and adjusting the heart-rate in order to maintain pressure within pre-set limits. This chronotropic mechanism might be enough to maintain homeostasis during small perturbations but, as in the case of real hearts, additional changes in contractility would probably be required during large perturbations. These could be implemented by, for example, modulating the L-type Ca^2+^ current in the electrophysiological model (Bodi et al., 2005) or accelerating the J_1_ transition (Supplementary Figure S1) to mimic the effects of increased phosphorylation of regulatory light chain (Kampourakis et al., 2016).

The final limitation that will be discussed is the one-way coupling of the electrophysiological and contractile models. As shown in Figure 1, the current framework uses ten Tusscher et al.’s electrophysiological model to predict the Ca^2+^ concentration in the myofibrillar space. More succinctly, the electrophysiology drives the contraction. Although this approach is practical, powerful, and nearly universal in current contractile modeling, it overlooks experimental data that show that quick length changes can perturb the intracellular Ca^2+^ concentration (Wakayama et al., 2001; Ter Keurs et al., 2006; Ter Keurs and Boyden, 2007). These experimental results imply that the contractile system can influence a cell’s electrophysiological status (presumably through variable buffering of Ca^2+^ ions by troponin) as well as the other way around. Again, this limitation could be corrected by re-writing the computer code so that the differential equations defining the electrophysiology were solved at the same time as the equations governing the contractile system. In practice, this would be a significant technical undertaking, and again beyond the scope of the current work.

## Data Availability Statement

All datasets generated for this study are included in the article/Supplementary Material.

## Author Contributions

KC planned the project, developed the computer software, and wrote the manuscript. BC developed the first prototype of the software and edited the manuscript. SC planned the project and edited the manuscript. All authors contributed to the article and approved the submitted version.

## Conflict of Interest

The authors declare that the research was conducted in the absence of any commercial or financial relationships that could be construed as a potential conflict of interest.
