# Supplementary material for: Multiscale Modeling of Cardiovascular Function Predicts That the End-Systolic Pressure Volume Relationship Can Be Targeted via Multiple Therapeutic Strategies
Source: Front Physiol. 2020 Aug 19;11:1043. doi: 10.3389/fphys.2020.01043 (PMC7466769; doi:10.3389/fphys.2020.01043)
Supplement: Supplementary file 1 [file Data_Sheet_1.pdf]

Supplementary Information

Multiscale modeling of Multiscale modeling of cardiovascular function predicts that the End-Systolic Pressure Volume Relationship can be targeted via multiple therapeutic strategies

[*1] Kenneth S. Campbell, [2,#] Brianna Sierra Chrisman, [2] Stuart G. Campbell

[1] Department of Physiology and Division of Cardiovascular Medicine, University of Kentucky, Lexington, Kentucky, United States of America

[2] Department of Biomedical Engineering, Yale University, New Haven, Connecticut, United States of America

[#] Current address: Department of Bioengineering, Stanford University, Stanford, California, United States of America

* Corresponding author

Email: k.s.campbell@uky.edu (KSC)

Additional details relating to the MyoSim contraction model

The MyoSim model used in this work was described in detail by Campbell et al. (Campbell et al., 2018). Key details are reproduced in this supplement to enhance clarity.

As shown in Fig S1, binding sites on the thin filament transitioned between an inactive state termed N_{off} (which myosin heads could not attach to) and an active state N_{on} (which was available for myosin binding). Activated binding sites could not switch back to the inactive state if a myosin head was attached. Myosin heads transitioned between an OFF state (that could not interact with actin), an ON state (that could potentially bind to actin), and a single attached force-generating state.

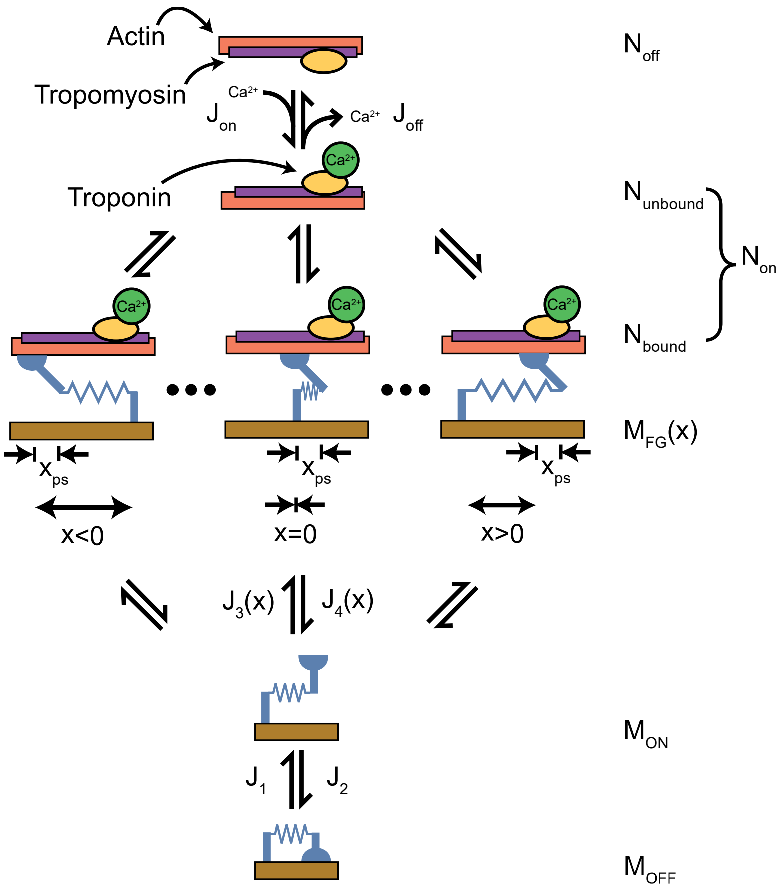

Fig S1: MyoSim kinetic scheme.

Sites on the thin filament switch between states that are available (N_{on}) and unavailable (N_{off}) for cross-bridges to bind to. Myosin heads transition between an OFF detached state, an ON detached state, and a single attached force-generating state. J terms indicate fluxes between different states.

Thin filament transitions

The fraction of binding sites in the active state was defined as $N_{on.}$ The flux for the N_{off} to N_{on} transition (that is, the number of sites per unit time switching from N_{off} to N_{on}) was defined as

$$J_{on} = k_{on}\left[Ca^{2+}\right]\left(N_{overlap} - N_{on}\right)\left(1 + k_{coop}\left(\frac{N_{on}}{N_{overlap}}\right)\right) \tag{S1}$$

where k_{on} is a rate constant, $N_{overlap}$ is the fraction of binding sites that are in range of myosin heads and depends on the prevailing half-sarcomere length as described in Campbell (Campbell, 2009), and k_{coop} is a constant that defines the strength of thin filament cooperativity.

The flux of binding sites through the off transition was defined as

$$J_{off} = k_{off}\left(N_{on} - N_{bound}\right)\left(1 + k_{coop}\left(\frac{N_{overlap} - N_{on}}{N_{overlap}}\right)\right) \tag{S2}$$

where k_{off} is a rate constant and the other terms are as defined previously. The (N_0-N_{bound}) term means that only unbound sites can deactivate (that is, myosin heads have to detach before a binding site can revert to the off state). The $k_{coop}((N_{overlap}$-$N_{on})/N_{overlap})$ term causes binding sites in the off state to induce further deactivation.

Thick filament transitions

The flux of myosin heads transitioning from the M_{OFF} state to the M_{ON} configuration was defined as

$$J_1 = k_1(1 + k_{force}F_{total})\,M_{OFF} \tag{S3}$$

where k_1 is a rate constant, k_{force} is a parameter with units of N^{-1} m^2, F_{total} is the force in the muscle, and M_{OFF} is the proportion of myosin heads in the OFF state.

The flux of myosin heads into the OFF state was defined as

$$J_2 = k_2 \, M_{ON} \tag{S4}$$

where k_2 is a rate constant and M_{ON} is the proportion of myosin heads in the ON state.

Myosin heads bound to actin with a flux J_3 defined as

$$J_3\left(x\right) = k_3 \, e^{\frac{-k_{cb}x^2}{2k_BT}} M_{ON}\left(N_{on} - N_{bound}\right) \tag{S5}$$

where k_3 is a rate constant, k_{cb} is the stiffness of the cross-bridge link, k_B is Boltzmann's constant (1.38×10^{-23} J K^{-1}) and T is the temperature. Equation 5 mimics a second order reaction so that the rate at which myosin heads attach to the thin filament increases with the product of the proportion of myosin heads that are able to attach (M_{ON}) and the fraction of available binding sites (N_{on}-N_{bound}). The Gaussian form of the myosin strain-dependence reflects the probability of the cross-bridge spring being extended to x by random Brownian motion when the myosin head binds to actin (Campbell and Lakie, 1998).

Similarly, the flux through the myosin detachment step was defined as

$$J_4\left(x\right) = \left(k_{4,0} + k_{4,1}\left(x - x_{ps}\right)^4\right) M_{FG}\left(x\right) \tag{S6}$$

where $k_{4,0}$ is a rate constant, $k_{4,1}$ is a parameter that sets the strain dependence of the cross-bridge detachment rate, x_{ps} is the power-stroke of an attached cross-bridge, and $M_{FG}(x)$ is the proportion of cross-bridges attached to actin with spring-lengths between x and x+δx.

Calculations

The kinetic scheme shown in Fig S1 was simulated by discretizing the relevant differential equations to yield

$$\begin{aligned}\frac{dN_{off}}{dt} &= -J_{on} + J_{off}\\ \frac{dN_{on}}{dt} &= J_{on} - J_{off}\\ \frac{dM_{OFF}}{dt} &= -J_1 + J_2\\ \frac{dM_{ON}}{dt} &= \left(J_1 + \sum_{i=1}^{n} J_{4,x_i}\right) - \left(J_2 + \sum_{i=1}^{n} J_{3,x_i}\right)\\ \frac{dM_{FG,i}}{dt} &= J_{3,x_i} - J_{4,x_i} \quad \text{where} \quad i=1\ldots n\end{aligned} \tag{S7}$$

Cross-bridge populations were evaluated with 0.5 nm resolution over the range -10 nm ≤ x ≤ 10 nm. n was thus equal to 41 giving a complete set of 45 equations. These were integrated numerically with adaptive step-size control. Calculations were initiated with all binding sites in the N_{off} configuration and all myosin heads in the M_{OFF} state.

Dynamic interfilamentary movement was incorporated into simulations when required by using polynomial interpolation to displace the distribution that described the number of heads bound with each spring length (Campbell, 2014). Filament compliance effects (Huxley et al., 1994;Wakabayashi et al., 1994) were mimicked by assuming that a half-sarcomere length change of Δx displaced each actin-myosin link by ½.Δx (Getz et al., 1998;Campbell, 2009).

Muscle force was calculated as

$$\begin{aligned}F_{total} &= F_{active} + F_{passive}\\ &\text{where}\\ F_{active} &= N_0 k_{cb} \sum_{i=1}^{n} M_{FG,i}\left(x_i + x_{ps}\right)\end{aligned} \tag{S8}$$

and N_0 is the number of myosin heads in a hypothetical cardiac half-sarcomere with a cross-sectional area of 1 m^2. N_0 was set to 6.9×10^{16} m^{-2} throughout this work based on the assumptions that (a) myofibrils occupy ~60%

of the cross-sectional area of myocardium, (b) half-thick filaments contain 283 myosin heads, and (c) half-thick filaments have a spatial density of 4.07×10^{14} m^{-2} within myofibrils (Linari et al., 2007;Campbell, 2014).

Passive force was calculated as

$$F_{passive} = \sigma\left(e^{\frac{x_{hs}-L_{slack}}{L}}-1\right) \qquad \text{(S9)}$$

where x_{hs} is the length of the half-sarcomere, and σ, L_{slack},, and L are model parameters. In this equation, σ is a scaling parameter, L_{slack} defines the length of the half-sarcomere at which passive force is zero, and L sets the curvature of the passive-force / length relationship. These properties reflect collagen content and the isoform and posttranslational status of titin which change with disease in humans (Hidalgo and Granzier, 2013;LeWinter and Granzier, 2014).

Myosin ATPase was calculated as

$$ATP_{ase} = \frac{N_0\, W_{volume}\, \Delta G`}{L_0\, N_A}\sum_{i=1}^{n} J_{4,x_i} \qquad \text{(S10)}$$

where ΔG` is the free energy produced by ATP hydrolysis (70 kJ mol^{-1}), L_0 is the reference length of half-sarcomere (1.1 μm), and N_A is Avogadro's number (6.02×10^{23} mol^{-1}). Ventricular efficiency was calculated as stroke work divided by myosin ATPase and omits the energy required to maintain the ionic gradients.

Circulation

Equation S11 defines the rate of change of the volume of each compartment in the circulatory model. Each term is simply the difference between the blood flows into and out of the compartment.

$$
\begin{aligned}
\frac{dV_{aorta}}{dt} &= Q_{ventricle\ to\ aorta} - Q_{aorta\ to\ arteries} \\
\frac{dV_{arteries}}{dt} &= Q_{aorta\ to\ arteries} - Q_{arteries\ to\ arterioles} \\
\frac{dV_{arterioles}}{dt} &= Q_{arteries\ to\ arterioles} - Q_{arterioles\ to\ capillaries} \\
\frac{dV_{capillaries}}{dt} &= Q_{arterioles\ to\ capillaries} - Q_{capillaries\ to\ veins} \\
\frac{dV_{veins}}{dt} &= Q_{capillaries\ to\ veins} - Q_{veins\ to\ ventricle} \\
\frac{dV_{ventricle}}{dt} &= Q_{veins\ to\ ventricle} - Q_{ventricle\ to\ aorta}
\end{aligned}
\qquad \text{(Eq S11)}
$$

Equation S12 defines the inter-compartmental flows. The aortic and mitral valves were simulated using simple condition statements based on the pressure gradient. Other flows were defined by Ohm’s law.

$$
\begin{aligned}
Q_{ventricle\ to\ aorta} &= \begin{cases} \dfrac{P_{ventricle} - P_{aorta}}{R_{aorta}} & \text{when} \quad P_{ventricle} \geq P_{aorta} \\ 0 & \text{otherwise} \end{cases} \\
Q_{aorta\ to\ arteries} &= \frac{P_{aorta} - P_{arteries}}{R_{arteries}} \\
Q_{arteries\ to\ arterioles} &= \frac{P_{arteries} - P_{arterioles}}{R_{arterioles}} \\
Q_{arterioles\ to\ capillaries} &= \frac{P_{arterioles} - P_{capillaries}}{R_{capillaries}} \\
Q_{capillaries\ to\ veins} &= \frac{P_{capillaries} - P_{veins}}{R_{veins}} \\
Q_{veins\ to\ ventricle} &= \begin{cases} \dfrac{P_{veins} - P_{ventricle}}{R_{ventricle}} & \text{when} \quad P_{veins} \geq P_{ventricle} \\ 0 & \text{otherwise} \end{cases}
\end{aligned}
\qquad \text{(Eq S12)}
$$

References for supplementary information

Campbell, K.S. (2009). Interactions between connected half-sarcomeres produce emergent behavior in a mathematical model of muscle. *PLOS Computational Biology* 5**,** e1000560. doi:1000510.1001371/journal.pcbi.1000560.

Campbell, K.S. (2014). Dynamic coupling of regulated binding sites and cycling myosin heads in striated muscle. *J Gen Physiol* 143**,** 387-399.

Campbell, K.S., Janssen, P.M.L., and Campbell, S.G. (2018). Force-Dependent Recruitment from the Myosin Off State Contributes to Length-Dependent Activation. *Biophys J* 115**,** 543-553.

Campbell, K.S., and Lakie, M. (1998). A cross-bridge mechanism can explain the thixotropic short-range elastic component of relaxed frog skeletal muscle. *Journal of Physiology* 510.3**,** 941-962.

Getz, E.B., Cooke, R., and Lehman, S.L. (1998). Phase transition in force during ramp stretches of skeletal muscle. *Biophysical Journal* 75**,** 2971-2983.

Hidalgo, C., and Granzier, H. (2013). Tuning the molecular giant titin through phosphorylation: role in health and disease. *Trends Cardiovasc Med* 23**,** 165-171.

Huxley, H.E., Stewart, A., Sosa, H., and Irving, T. (1994). X-ray diffraction measurements of the extensibility of actin and myosin filaments in contracting muscle. *Biophysical Journal* 67**,** 2411-2421.

Lewinter, M.M., and Granzier, H.L. (2014). Cardiac titin and heart disease. *J Cardiovasc Pharmacol* 63**,** 207-212.

Linari, M., Caremani, M., Piperio, C., Brandt, P., and Lombardi, V. (2007). Stiffness and fraction of myosin motors responsible for active force in permeabilized muscle fibers from rabbit psoas. *Biophysical Journal* 92**,** 2476-2490.

Mirsky, I., Tajimi, T., and Peterson, K.L. (1987). The development of the entire end-systolic pressure-volume and ejection fraction-afterload relations: a new concept of systolic myocardial stiffness. *Circulation* 76**,** 343-356.

Ten Tusscher, K.H., Noble, D., Noble, P.J., and Panfilov, A.V. (2004). A model for human ventricular tissue. *Am J Physiol Heart Circ Physiol* 286**,** H1573-1589.

Wakabayashi, K., Sugimoto, Y., Tanaka, H., Ueno, Y., Takezawa, Y., and Amemiya, Y. (1994). X-ray diffraction evidence for the extensibility of actin and myosin filaments during muscle contraction. *Biophysical Journal* 67**,** 2422-2435.

Table S1: Base parameter values

Component	Parameter	Value	Units
MyoSim	k_{on}	5×10^8	M^{-1} s^{-1}
	k_{off}	200	s^{-1}
	k_{coop}	5	Dimensionless
	k_1	2	s^{-1}
	k_2	200	s^{-1}
	k_3	100	s^{-1} nm^{-1}
	$k_{4,0}$	200	s^{-1}
	$k_{4,1}$	0.1	s^{-1} nm^{-4}
	k_{cb}	0.001	pN nm^{-1}
	x_{ps}	5	nm
	$k_{falloff}$	0.0024	nm^{-1}
	σ	500	N m^{-2}
	L	80	nm
	L_{slack}	900	nm
Ventricle	W_{volume}	0.1	L
	V_{slack}	0.08	L
	$R_{ventricle}$	20	mm Hg L^{-1} s
Circulation	V_{total}	5	L
	R_{aorta}	40	mm Hg L^{-1} s
	$R_{arteries}$	200	mm Hg L^{-1} s
	$R_{arterioles}$	500	mm Hg L^{-1} s
	$R_{capillaries}$	300	mm Hg L^{-1} s
	R_{veins}	100	mm Hg L^{-1} s
	C_{aorta}	0.002	$(mm Hg)^{-1}$ L
	$C_{arteries}$	0.0005	$(mm Hg)^{-1}$ L
	$C_{arterioles}$	0.0005	$(mm Hg)^{-1}$ L
	$C_{capillaries}$	0.0025	$(mm Hg)^{-1}$ L
	C_{veins}	0.35	$(mm Hg)^{-1}$ L

Base values for the electrophysiological model remained as published by ten Tusscher et al. (ten Tusscher et al., 2004).

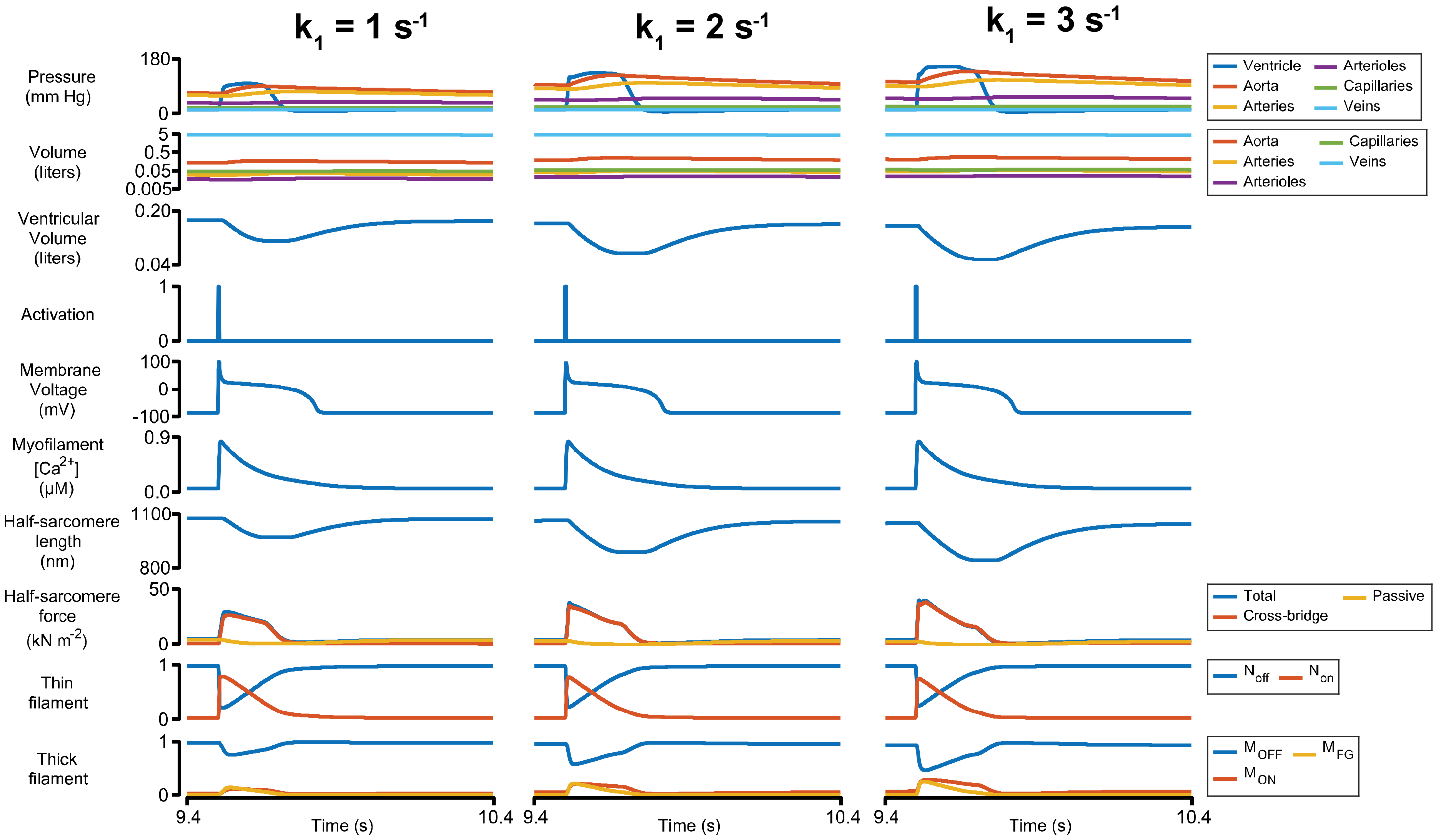

Fig S2. Steady-state beats for three different values of k_1.

This figure is similar to Fig 3 in the main text but shows steady-state beats for simulations with 3 different values of k_1. Note that the systolic pressure increases with k_1 over this limited range.

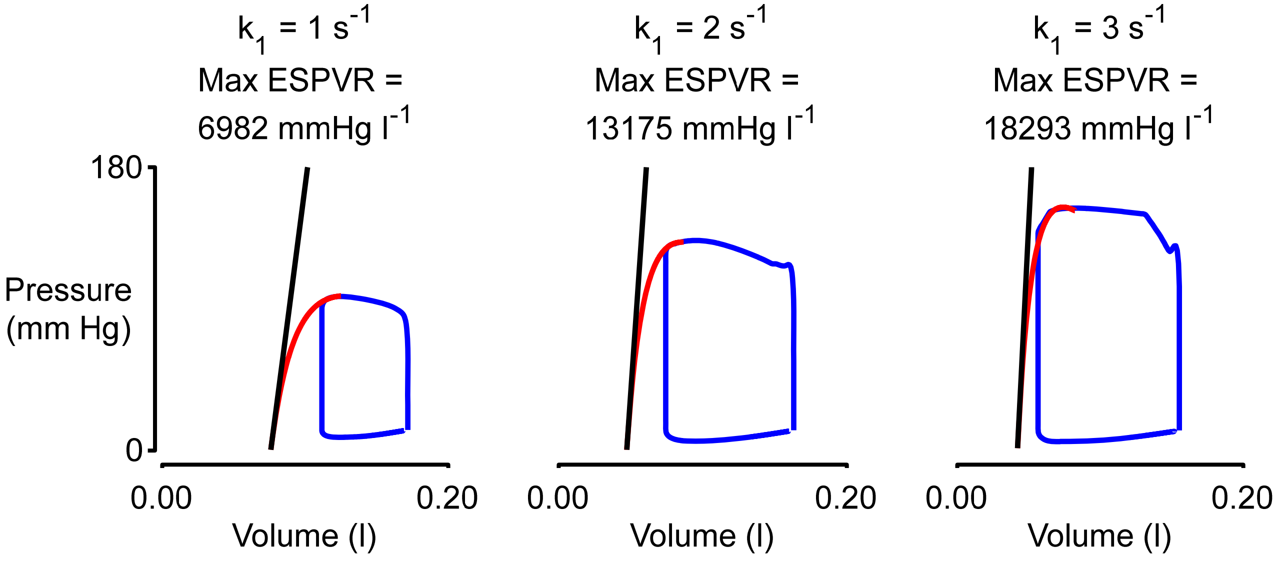

Fig S3. Single beat analysis of ESPVR

Panels show the single beats from Fig S2 as pressure-volume loops. The maximum slope of the ESPVR was calculated as described by Mirsky et al. (Mirsky et al., 1987) by extrapolating a fit to the pressure-volume data from the late phase of ejection to a ventricular pressure of zero. Note that the ESPVR increased with k_1 over this range as shown in Fig 5.

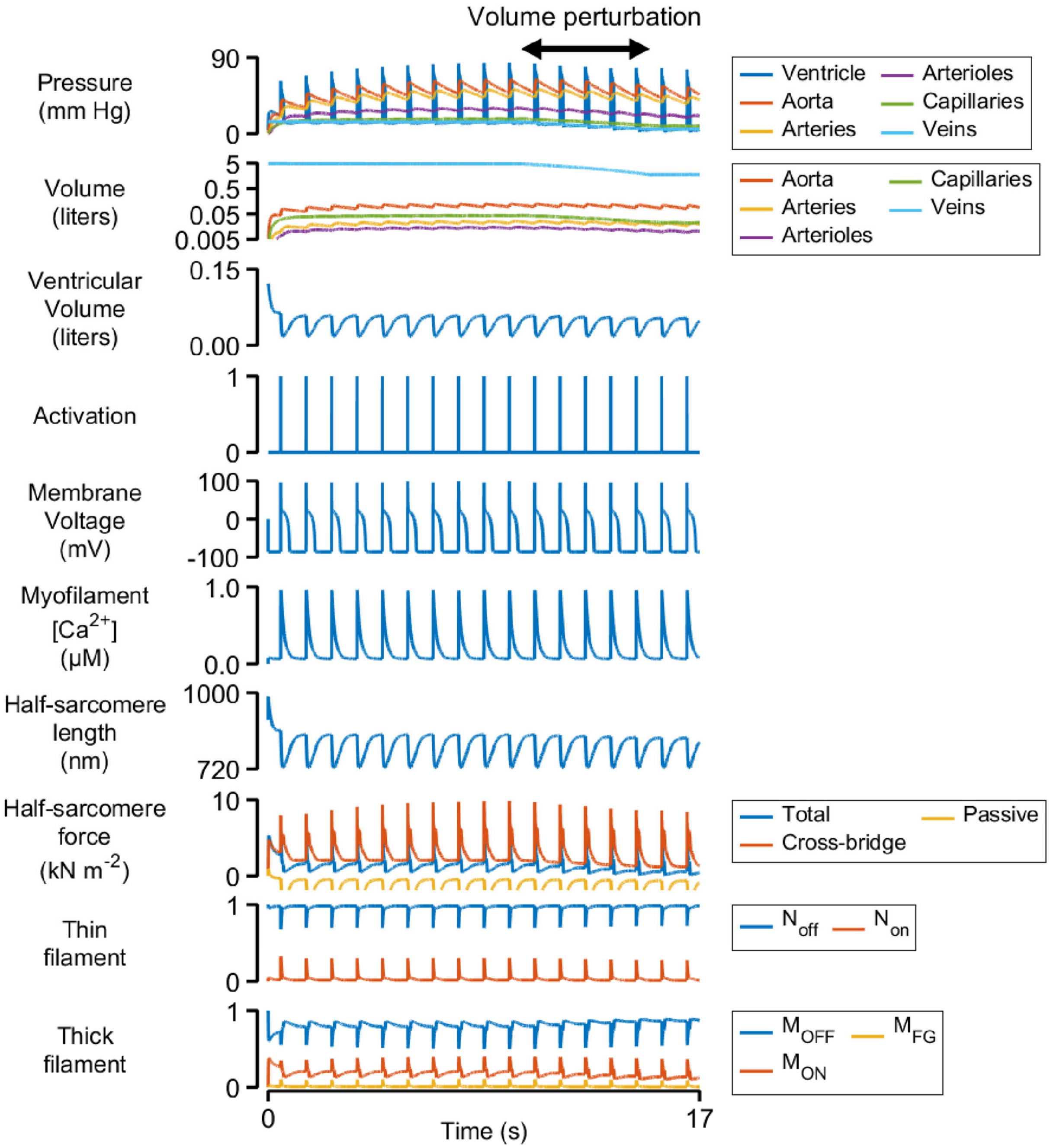

Fig S4. Excess contractility can reduce cardiac function.

The figure shows a simulation identical to Fig 2 in the main text but with the value of k_1 increased 10-fold. This modification activated cross-bridge force generation between Ca^{2+} transients and prevented the ventricle from filling adequately during diastole. In this simulation, enhanced contractility depresses stroke volume.

L

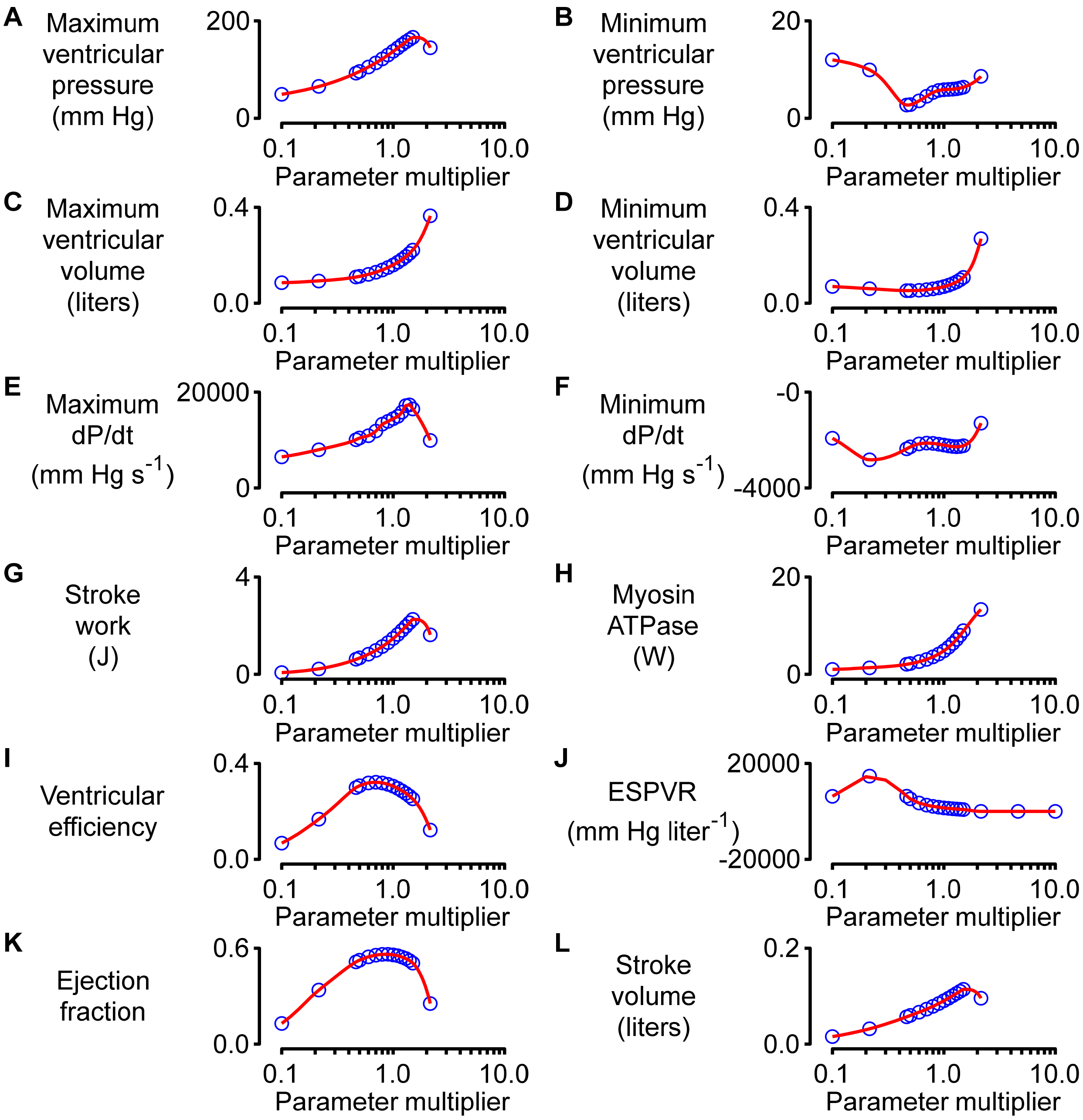

Figure S5: Effects of changing L on system-level cardiovascular properties.

Panels A to L show values (blue circles) for 12 system-level properties (for example, maximum ventricular pressure) predicted for values of L (equation S9) ranging from 0.1 to 10 times the value shown in Table S1. The red lines shows the best-fit of a 5th order polynomial to the simulated data.

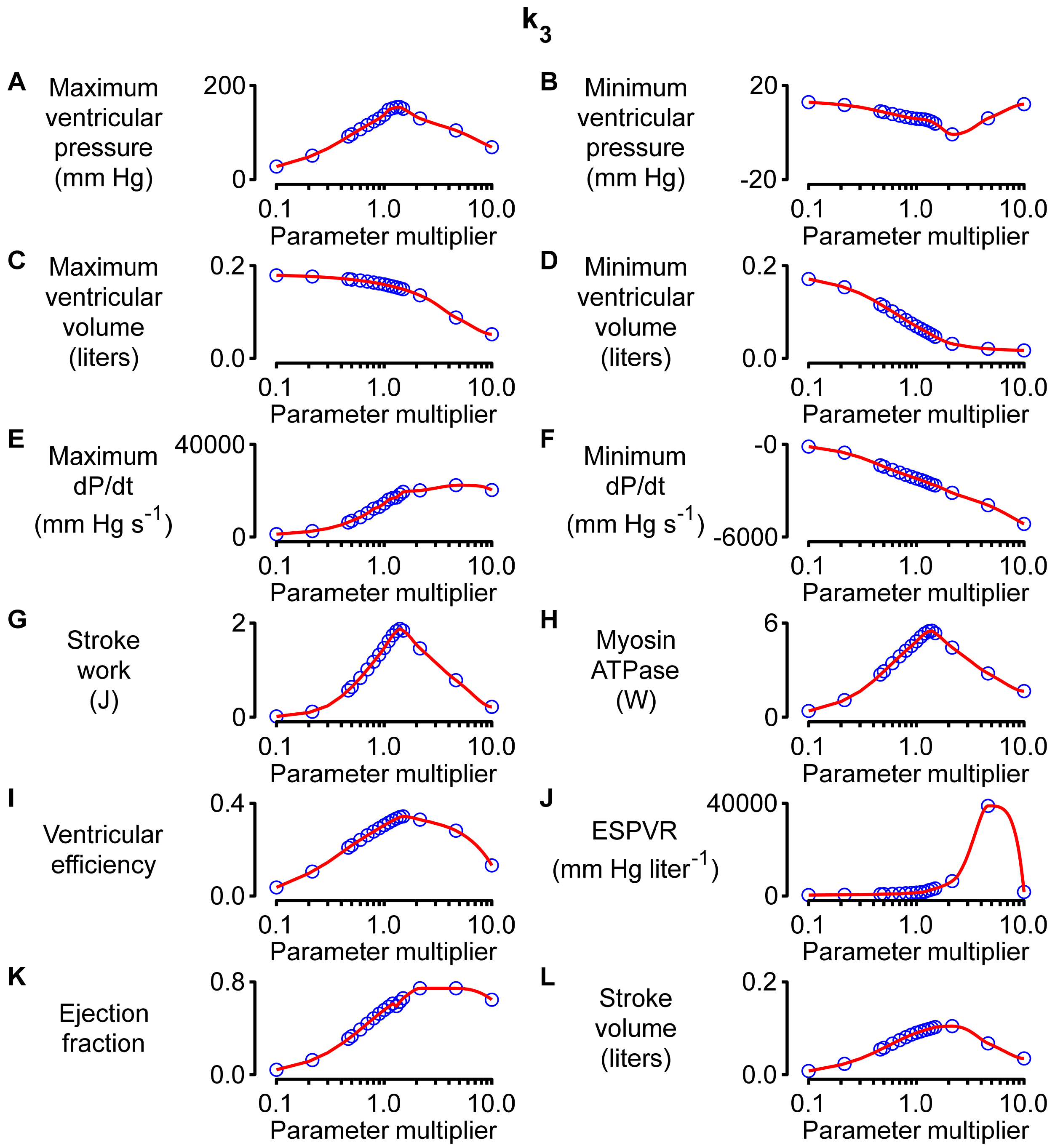

Figure S6: Effects of changing k_3 on system-level cardiovascular properties.

Panels A to L show values (blue circles) for 12 system-level properties (for example, maximum ventricular pressure) predicted for values of k_3 (equation S5) ranging from 0.1 to 10 times the value shown in Table S1. The red lines shows the best-fit of a 5th order polynomial to the simulated data.

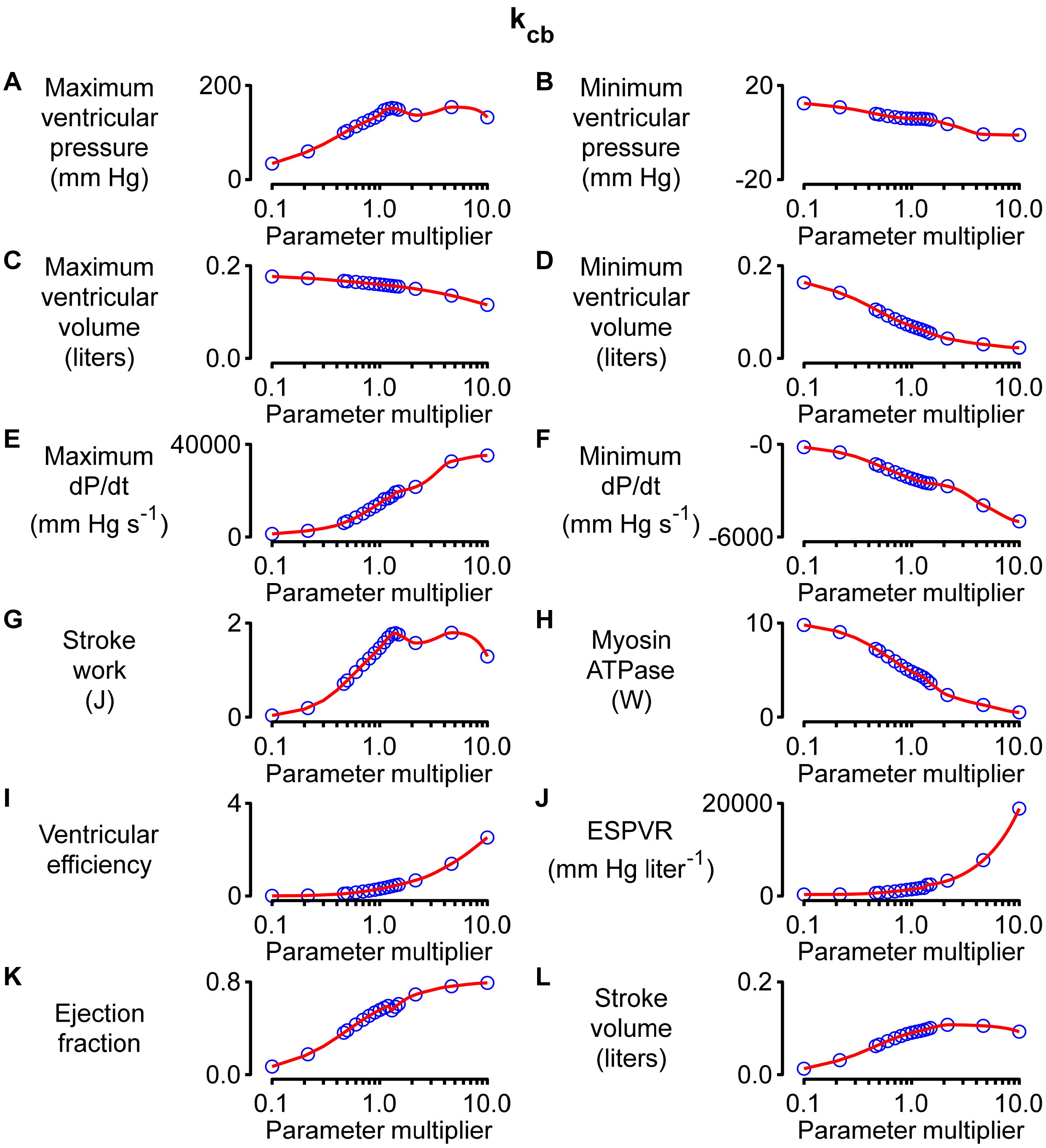

Figure S7: Effects of changing k_{cb} on system-level cardiovascular properties.

Panels A to L show values (blue circles) for 12 system-level properties (for example, maximum ventricular pressure) predicted for values of k_{cb} (equation S5) ranging from 0.1 to 10 times the value shown in Table S1. The red lines shows the best-fit of a 5th order polynomial to the simulated data.

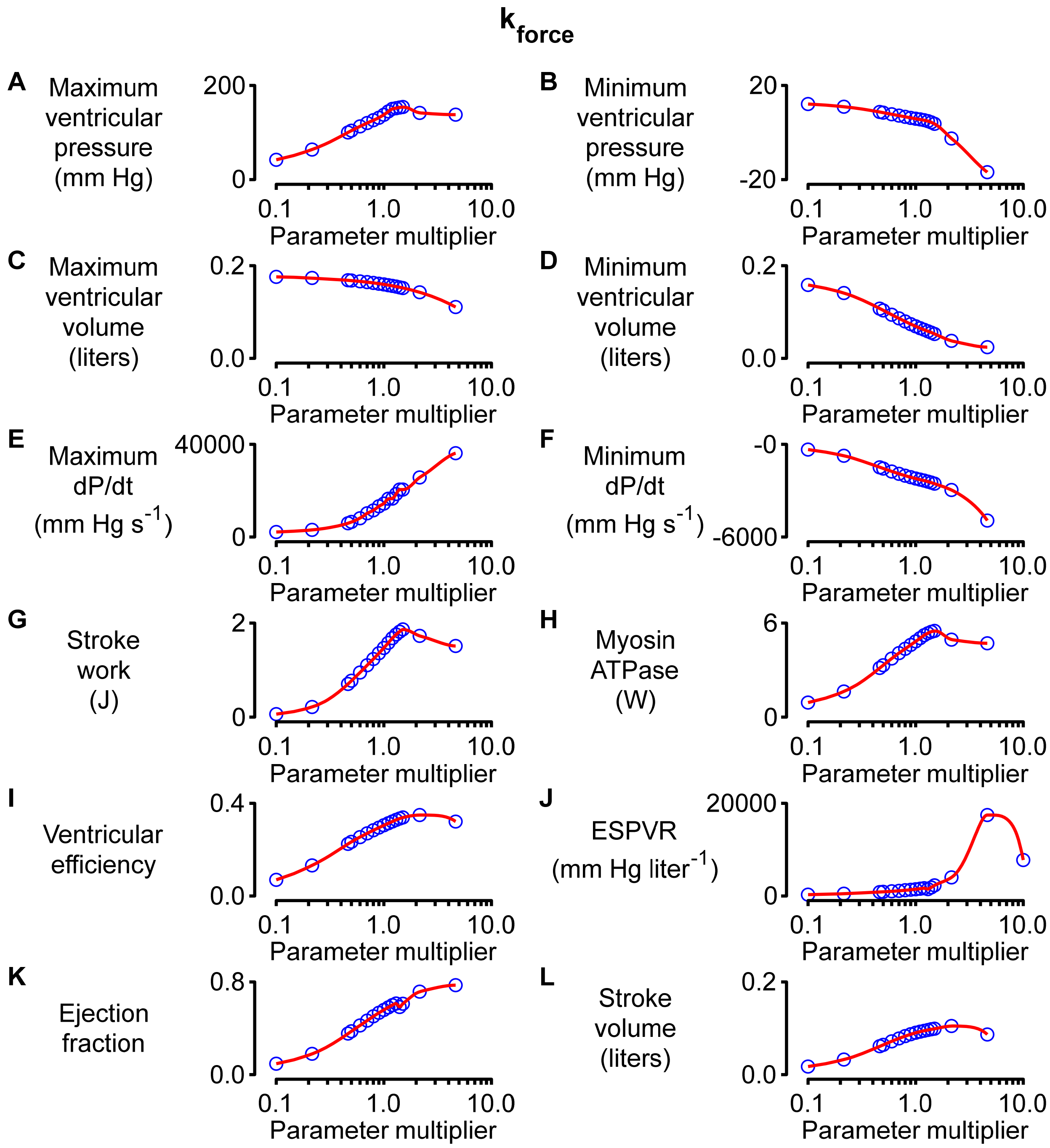

Figure S8: Effects of changing k_{force} on system-level cardiovascular properties.

Panels A to L show values (blue circles) for 12 system-level properties (for example, maximum ventricular pressure) predicted for values of k_{force} (equation S3) ranging from 0.1 to 10 times the value shown in Table S1. The red lines shows the best-fit of a 5th order polynomial to the simulated data.

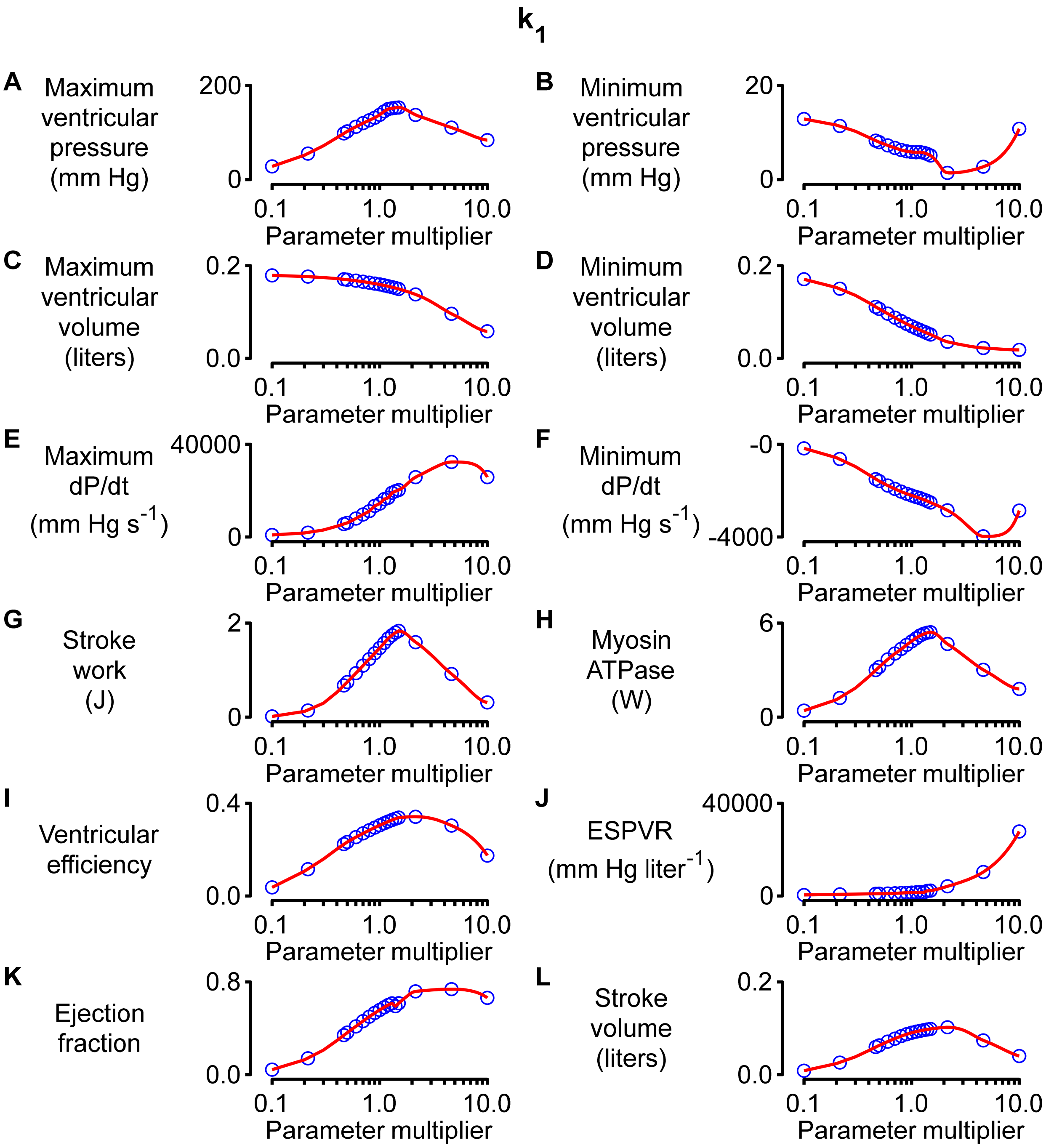

Figure S9: Effects of changing k_1 on system-level cardiovascular properties.

Panels A to L show values (blue circles) for 12 system-level properties (for example, maximum ventricular pressure) predicted for values of k_1 (equation S3) ranging from 0.1 to 10 times the value shown in Table S1. The red lines shows the best-fit of a 5th order polynomial to the simulated data.

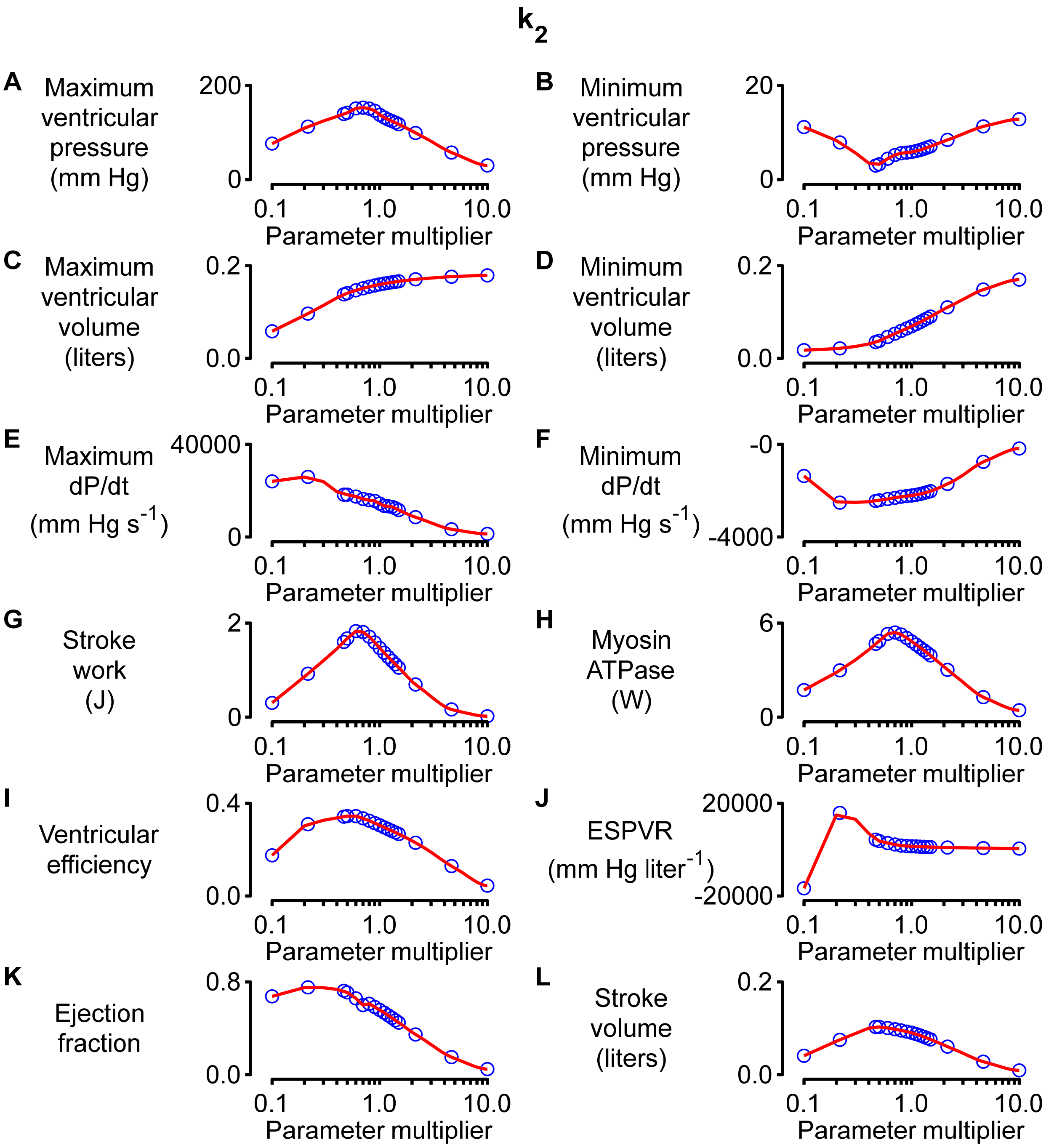

Figure S10: Effects of changing k_2 on system-level cardiovascular properties.

Panels A to L show values (blue circles) for 12 system-level properties (for example, maximum ventricular pressure) predicted for values of k_2 (equation S4) ranging from 0.1 to 10 times the value shown in Table S1. The red lines shows the best-fit of a 5th order polynomial to the simulated data.

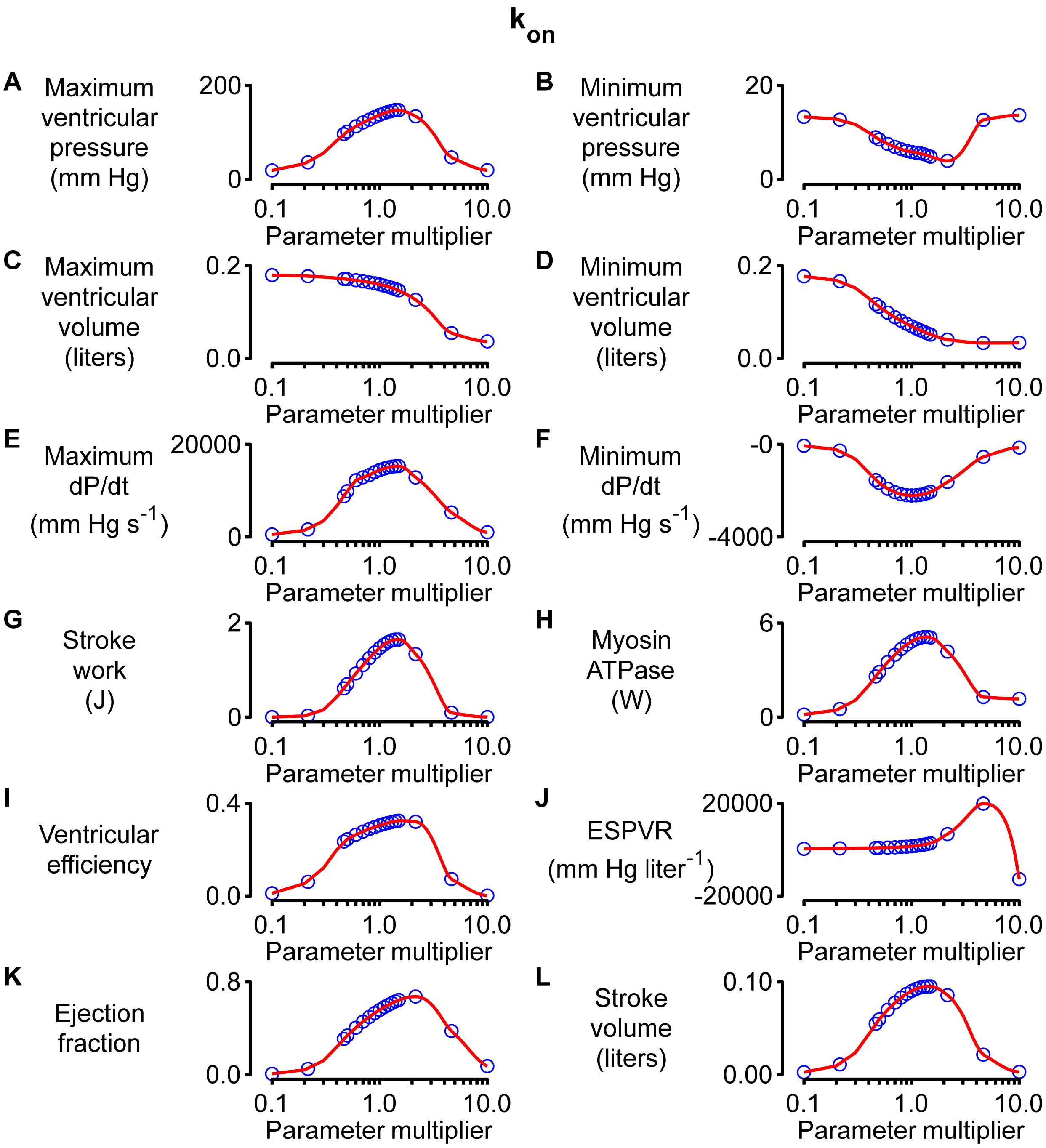

Figure S11: Effects of changing k_{on} on system-level cardiovascular properties.

Panels A to L show values (blue circles) for 12 system-level properties (for example, maximum ventricular pressure) predicted for values of k_{on} (equation S1) ranging from 0.1 to 10 times the value shown in Table S1. The red lines shows the best-fit of a 5th order polynomial to the simulated data.

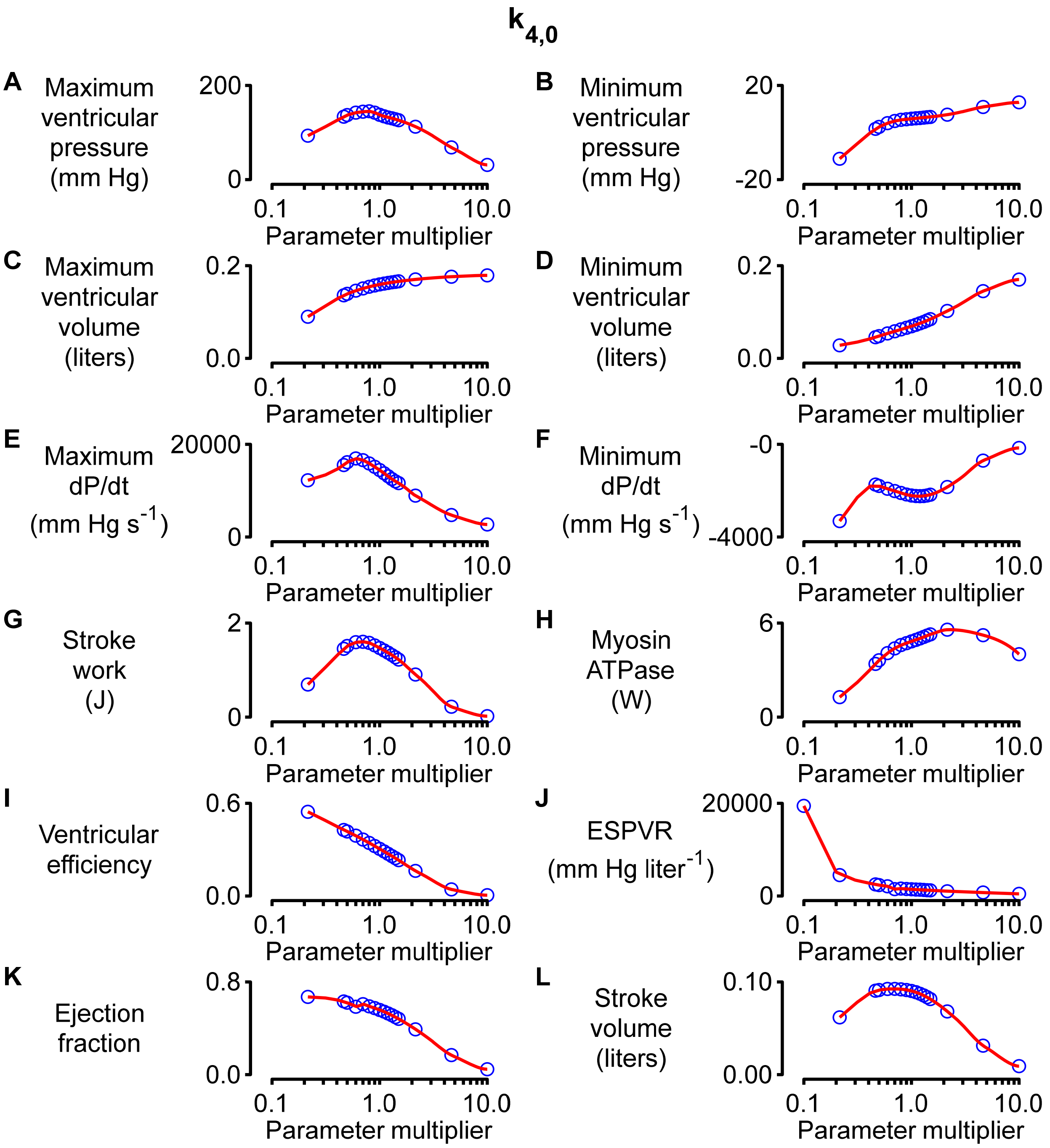

Figure S12: Effects of changing $k_{4,0}$ on system-level cardiovascular properties.

Panels A to L show values (blue circles) for 12 system-level properties (for example, maximum ventricular pressure) predicted for values of $k_{4,0}$ (equation S6) ranging from 0.1 to 10 times the value shown in Table S1. The red lines shows the best-fit of a 5th order polynomial to the simulated data.

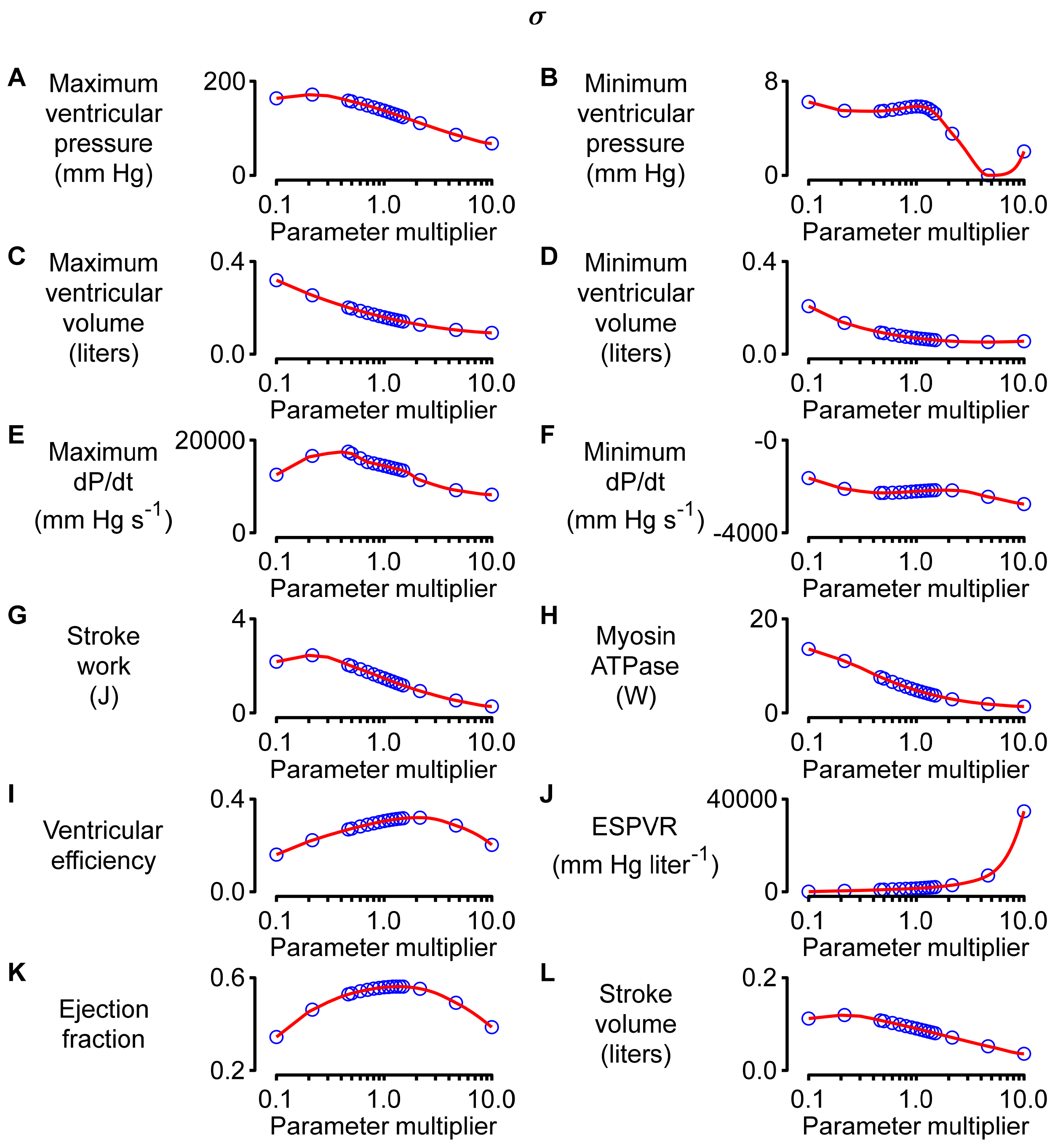

Figure S13: Effects of changing σ on system-level cardiovascular properties.

Panels A to L show values (blue circles) for 12 system-level properties (for example, maximum ventricular pressure) predicted for values of σ (equation S9) ranging from 0.1 to 10 times the value shown in Table S1. The red lines shows the best-fit of a 5th order polynomial to the simulated data.

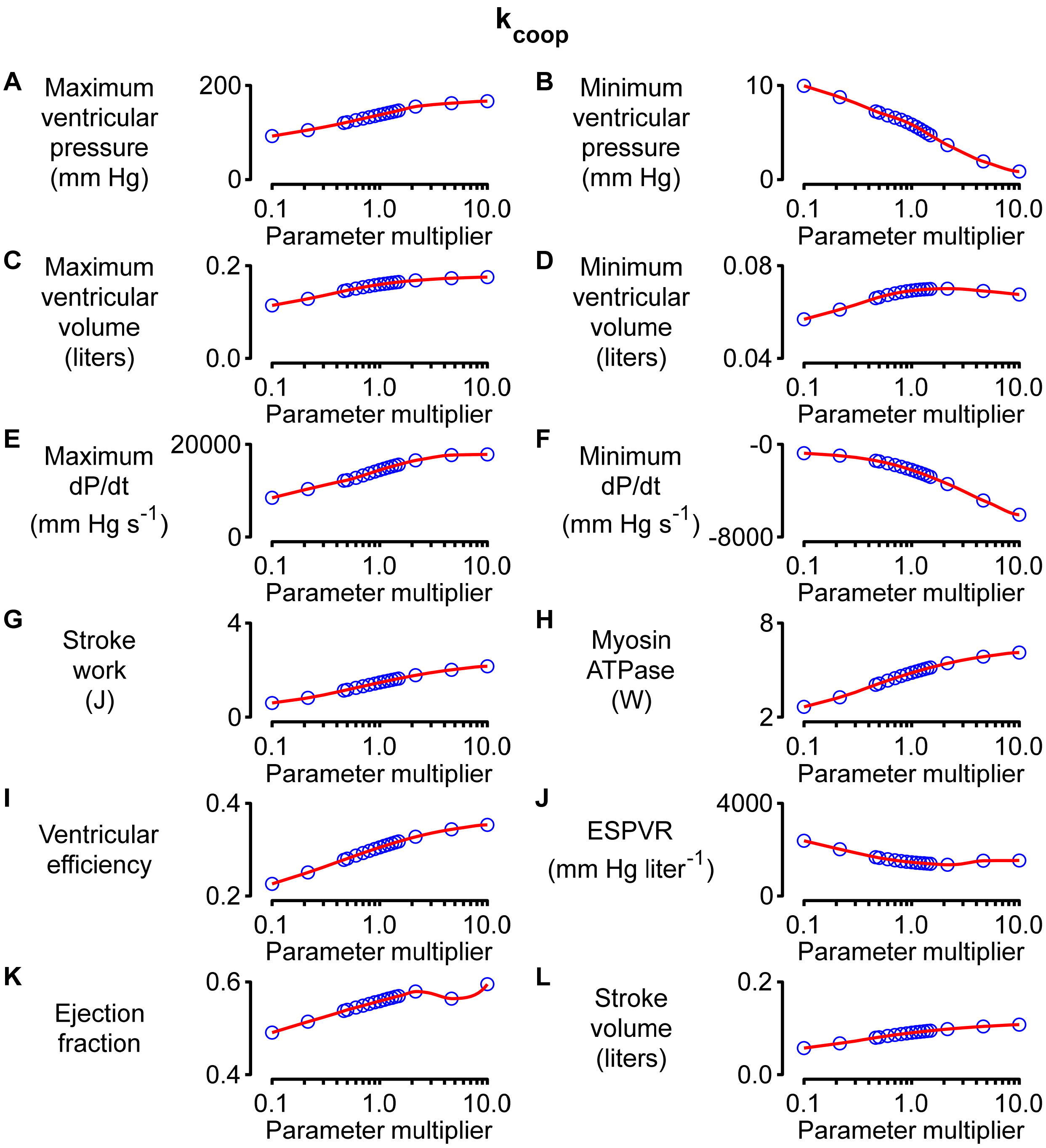

Figure S14: Effects of changing k_{coop} on system-level cardiovascular properties.

Panels A to L show values (blue circles) for 12 system-level properties (for example, maximum ventricular pressure) predicted for values of k_{coop} (equations S1 and S2) ranging from 0.1 to 10 times the value shown in Table S1. The red lines shows the best-fit of a 5th order polynomial to the simulated data.

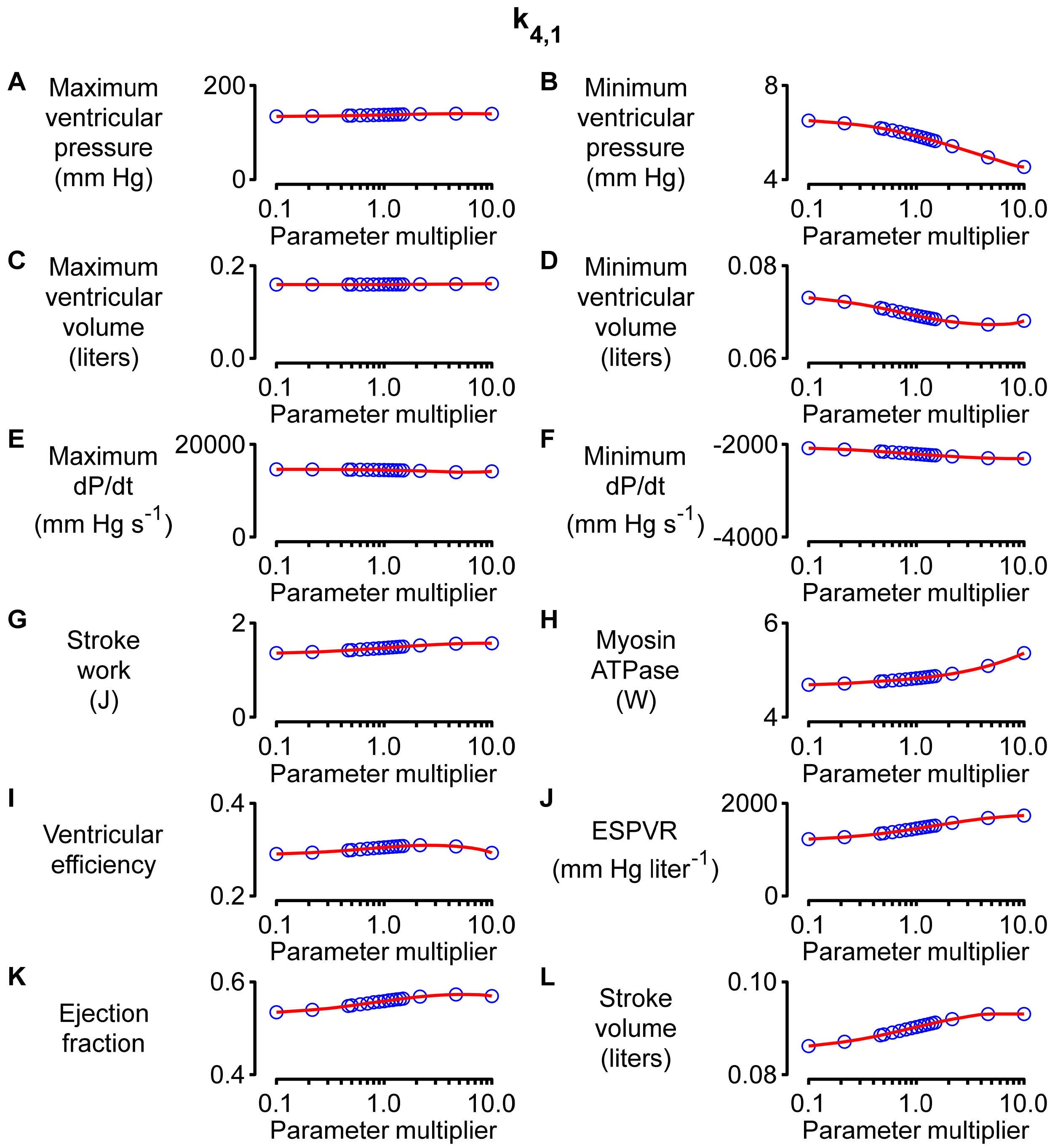

Figure S15: Effects of changing $k_{4,1}$ on system-level cardiovascular properties.

Panels A to L show values (blue circles) for 12 system-level properties (for example, maximum ventricular pressure) predicted for values of $k_{4,1}$ (equation S6) ranging from 0.1 to 10 times the value shown in Table S1. The red lines shows the best-fit of a 5th order polynomial to the simulated data.

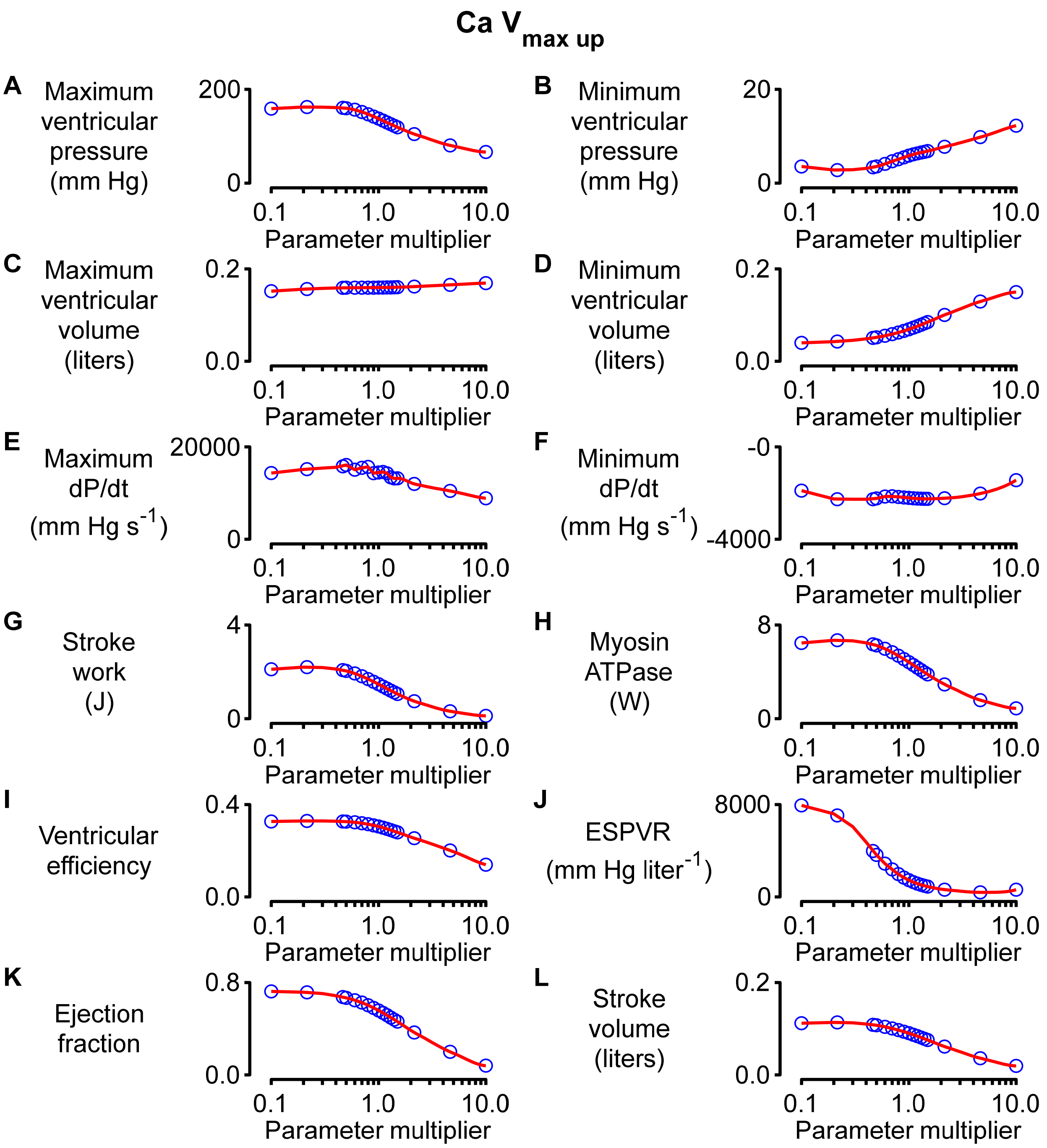

Figure S16: Effects of changing Ca $V_{max\ up}$ on system-level cardiovascular properties.

Panels A to L show values (blue circles) for 12 system-level properties (for example, maximum ventricular pressure) predicted for values of Ca $V_{max\ up}$ ranging from 0.1 to 10 times the base value in ten Tusscher et al's electrophysiological model (ten Tusscher et al., 2004). The red lines shows the best-fit of a 5th order polynomial to the simulated data.

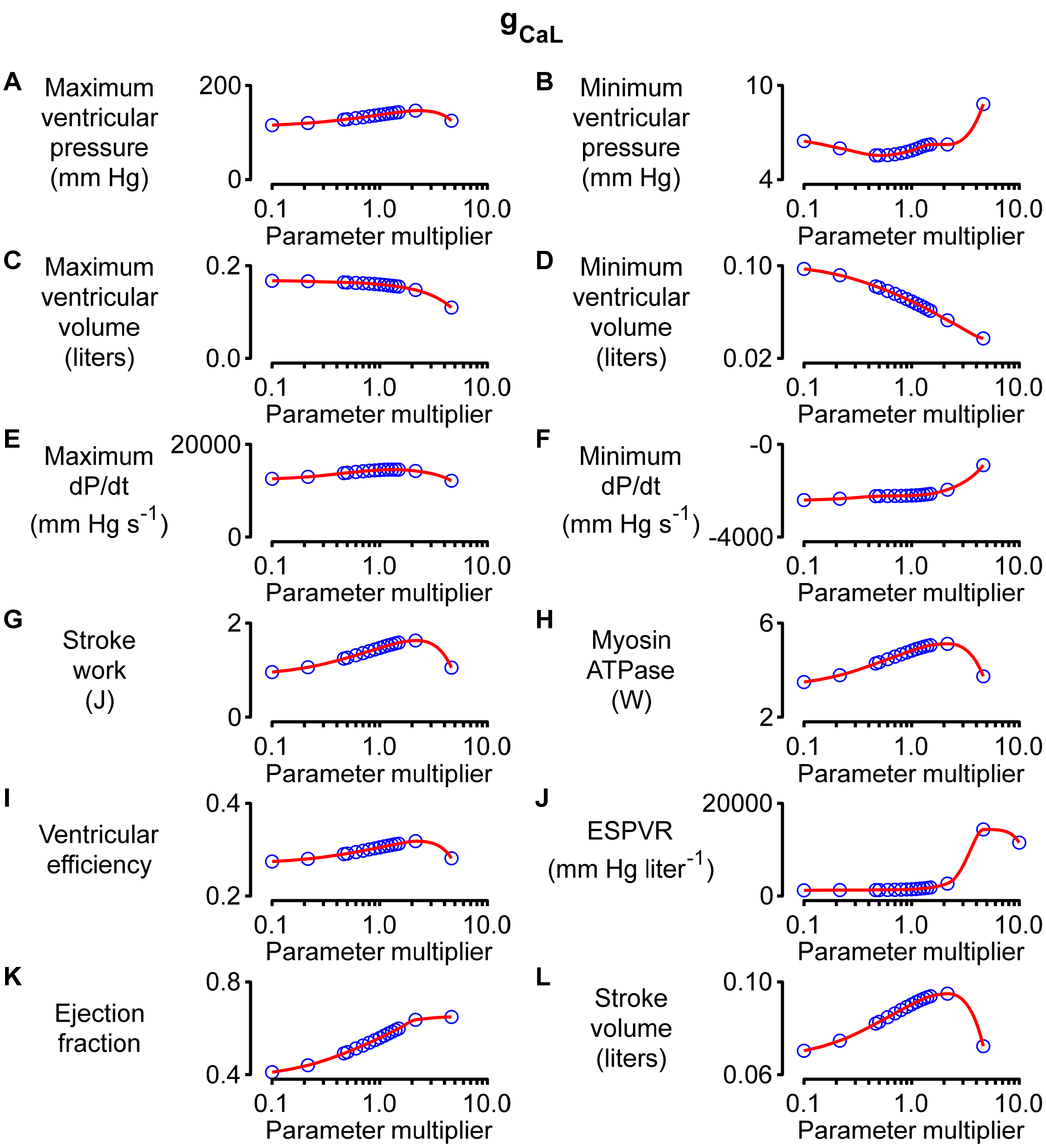

Figure S17: Effects of changing Ca g_{CaL} on system-level cardiovascular properties.

Panels A to L show values (blue circles) for 12 system-level properties (for example, maximum ventricular pressure) predicted for values of Ca g_{CaL} ranging from 0.1 to 10 times the base value in ten Tusscher et al's electrophysiological model (ten Tusscher et al., 2004). The red lines shows the best-fit of a 5th order polynomial to the simulated data.

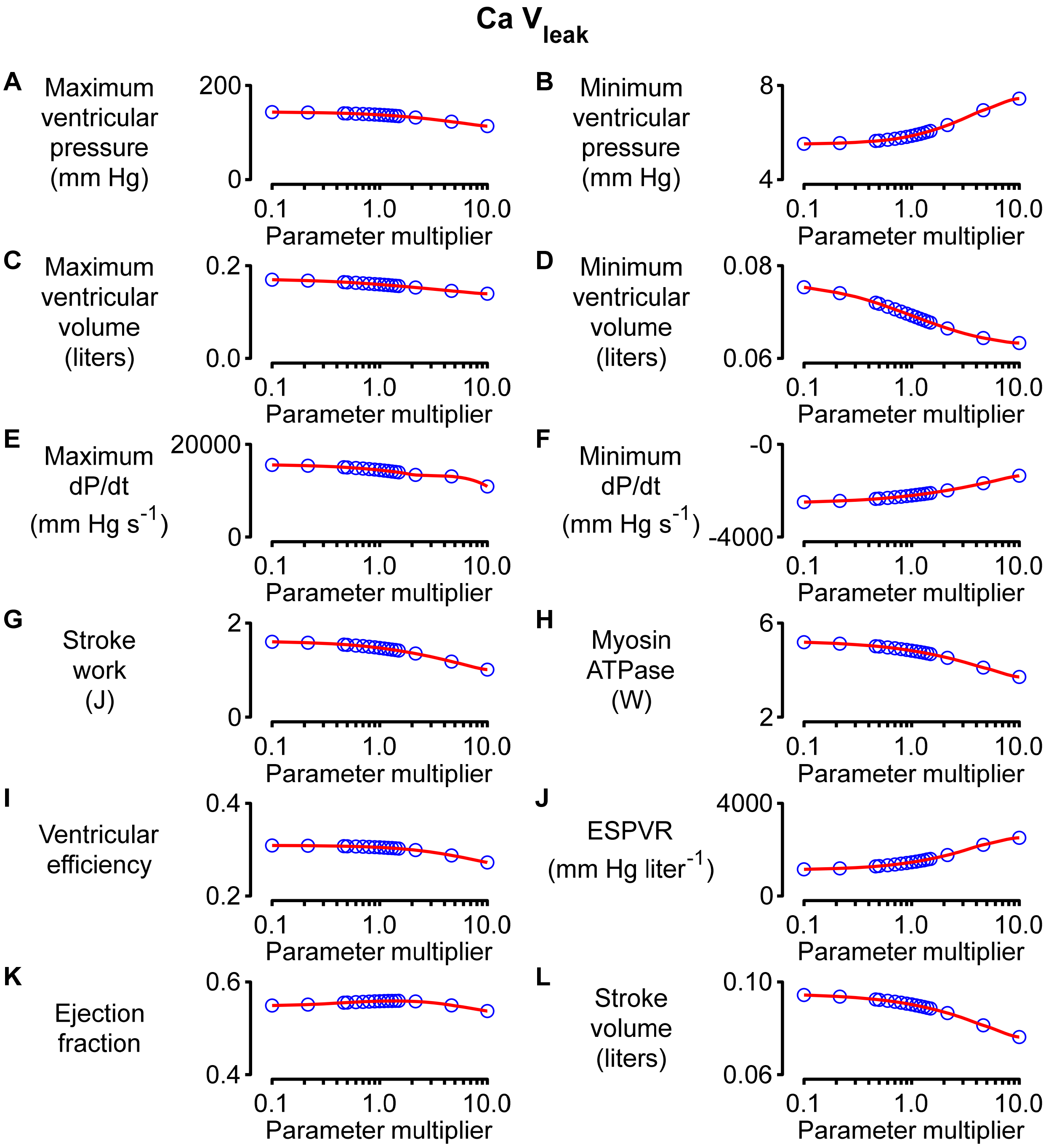

Figure S18: Effects of changing Ca V_{leak} on system-level cardiovascular properties.

Panels A to L show values (blue circles) for 12 system-level properties (for example, maximum ventricular pressure) predicted for values of Ca V_{leak} ranging from 0.1 to 10 times the base value in ten Tusscher et al's electrophysiological model (ten Tusscher et al., 2004). The red lines shows the best-fit of a 5th order polynomial to the simulated data.

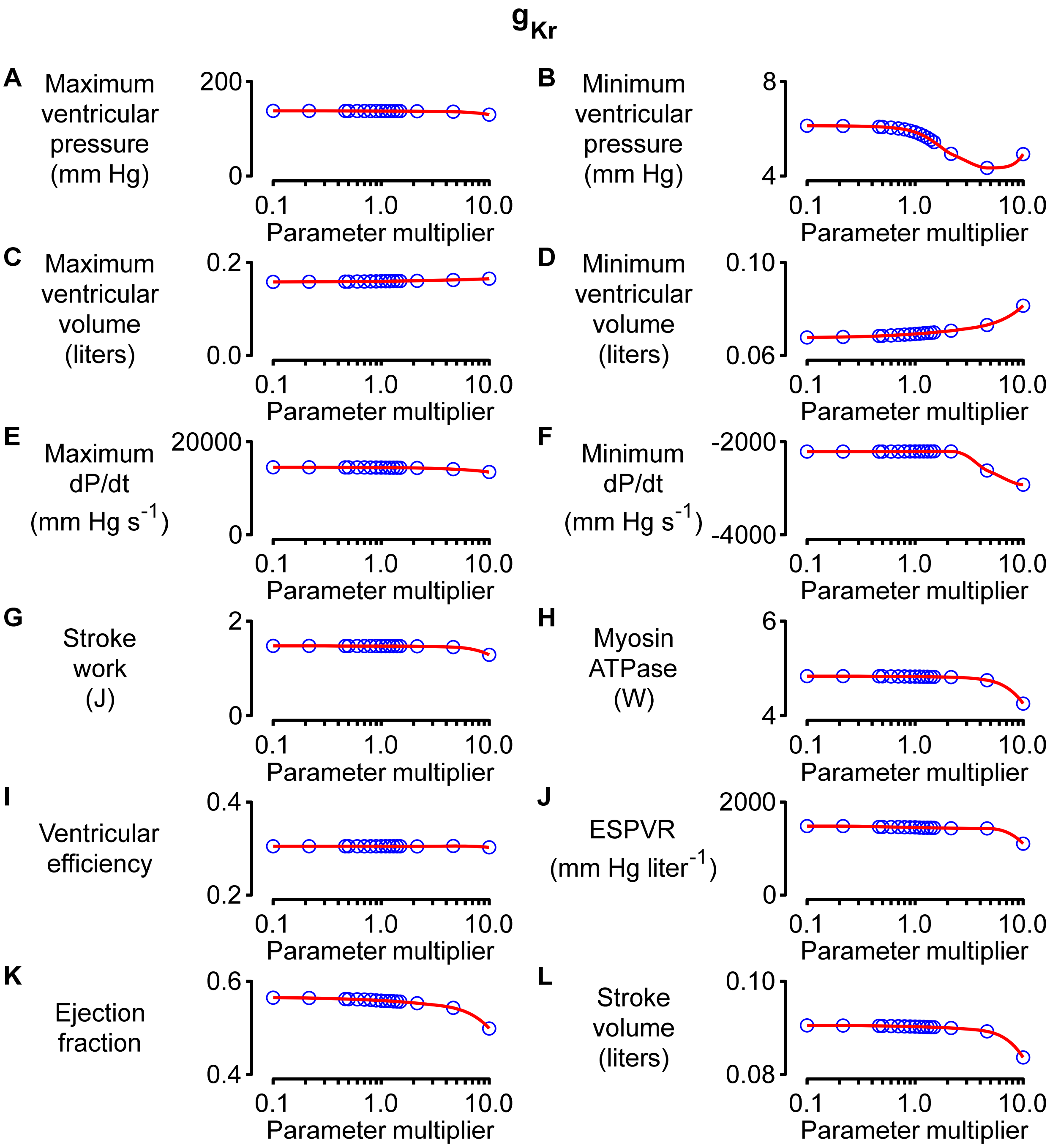

Figure S19: Effects of changing g_{Kr} on system-level cardiovascular properties.

Panels A to L show values (blue circles) for 12 system-level properties (for example, maximum ventricular pressure) predicted for values of g_{Kr} ranging from 0.1 to 10 times the base value in ten Tusscher et al's electrophysiological model (ten Tusscher et al., 2004). The red lines shows the best-fit of a 5th order polynomial to the simulated data.

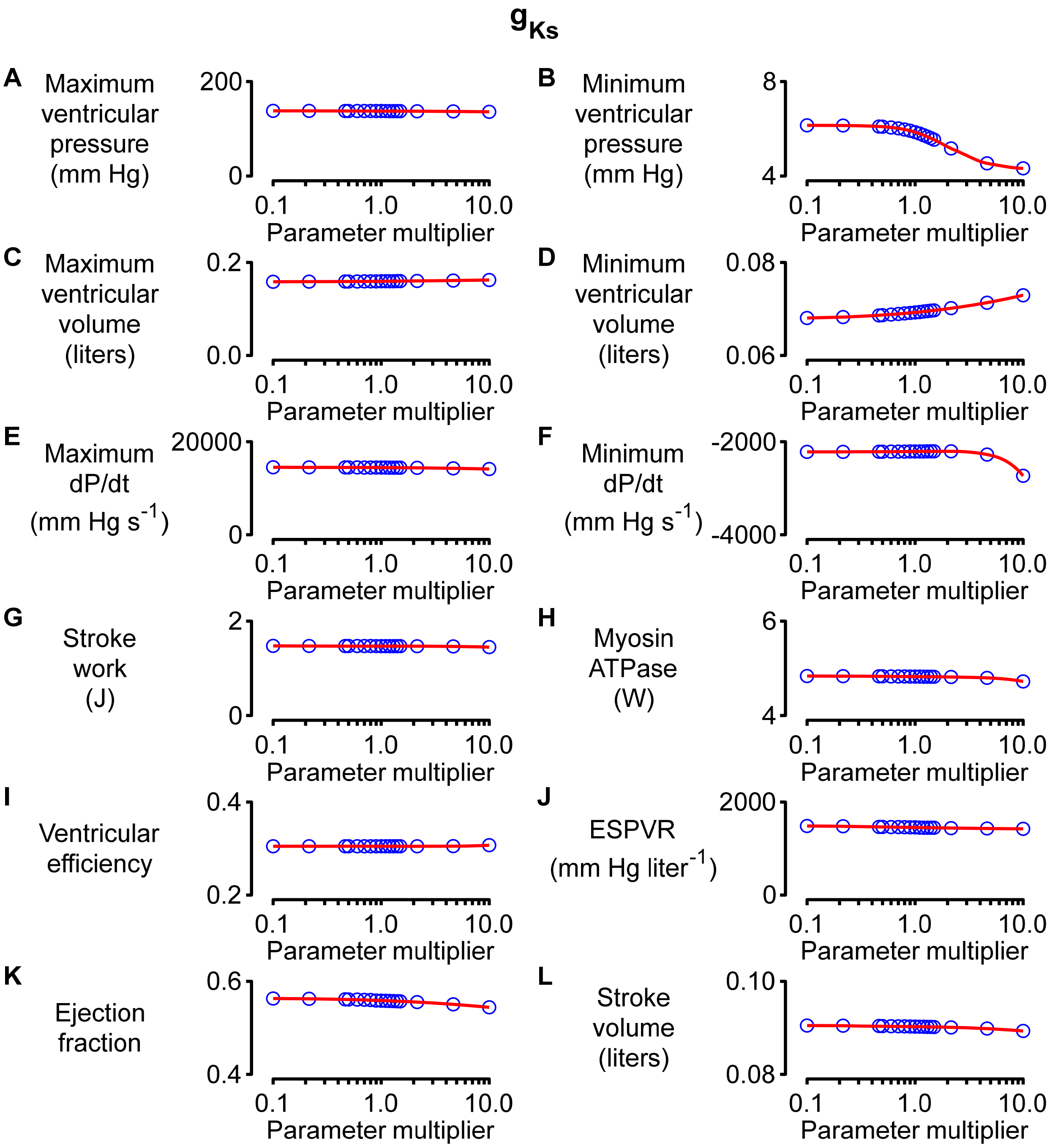

Figure S20: Effects of changing g_{Ks} on system-level cardiovascular properties.

Panels A to L show values (blue circles) for 12 system-level properties (for example, maximum ventricular pressure) predicted for values of g_{Ks} ranging from 0.1 to 10 times the base value in ten Tusscher et al's electrophysiological model (ten Tusscher et al., 2004). The red lines shows the best-fit of a 5th order polynomial to the simulated data.

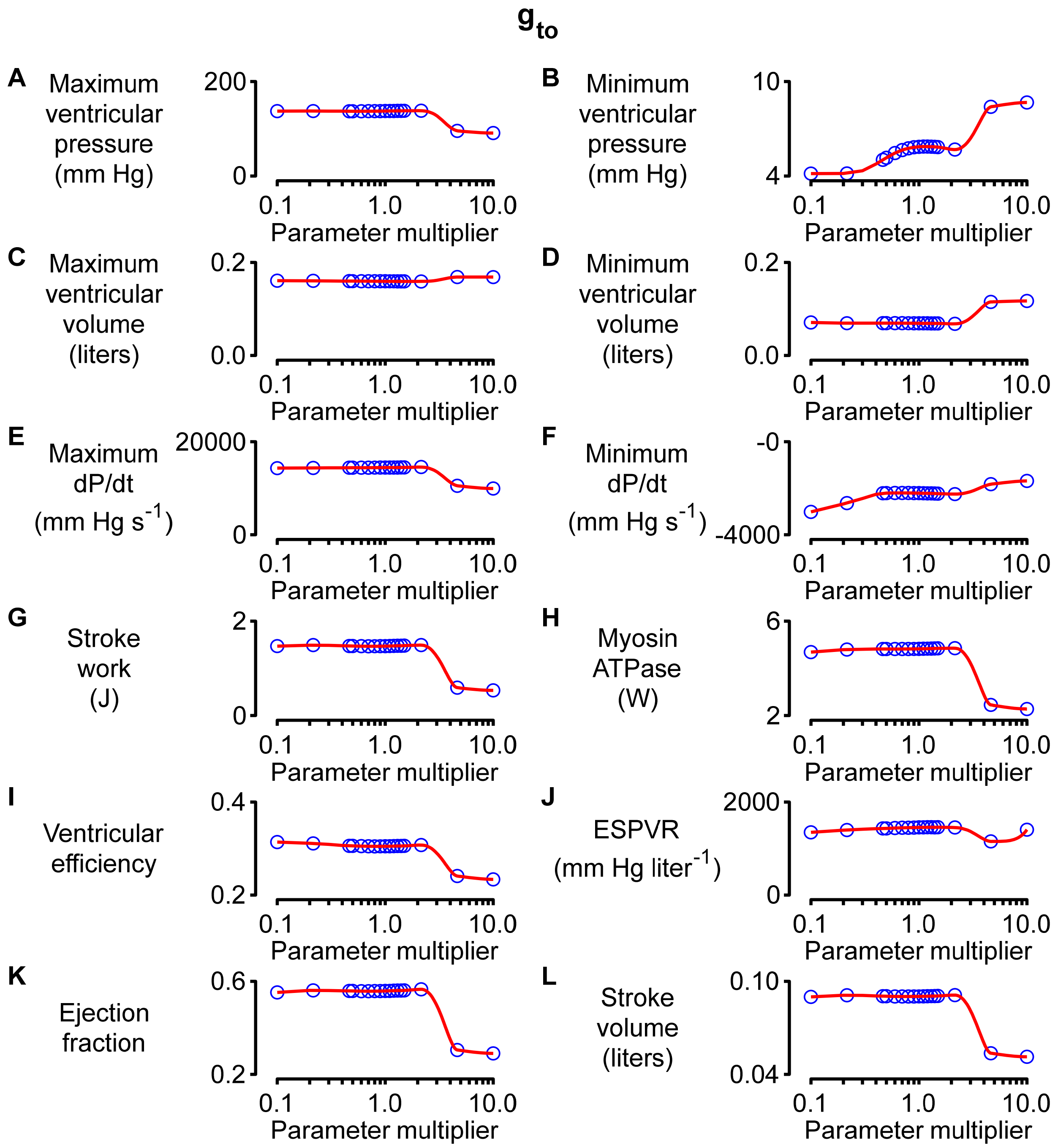

Figure S21: Effects of changing g_{to} on system-level cardiovascular properties.

Panels A to L show values (blue circles) for 12 system-level properties (for example, maximum ventricular pressure) predicted for values of g_{to} ranging from 0.1 to 10 times the base value in ten Tusscher et al's electrophysiological model (ten Tusscher et al., 2004). The red lines shows the best-fit of a 5th order polynomial to the simulated data.

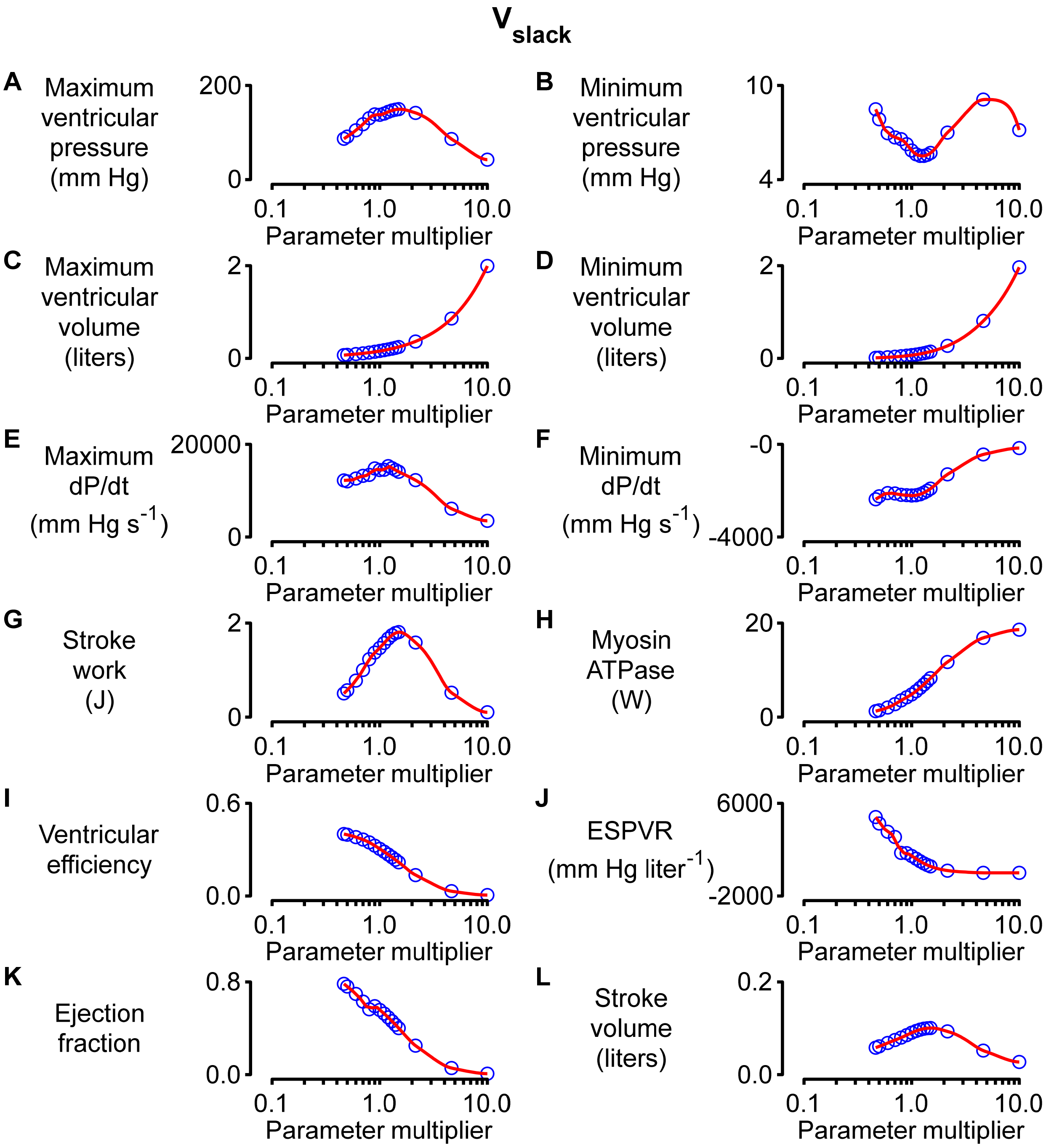

Figure S22: Effects of changing V_{slack} on system-level cardiovascular properties.

Panels A to L show values (blue circles) for 12 system-level properties (for example, maximum ventricular pressure) predicted for values of V_{slac} (see Methods in main text) ranging from 0.1 to 10 times the value shown in Table S1. The red lines shows the best-fit of a 5th order polynomial to the simulated data.

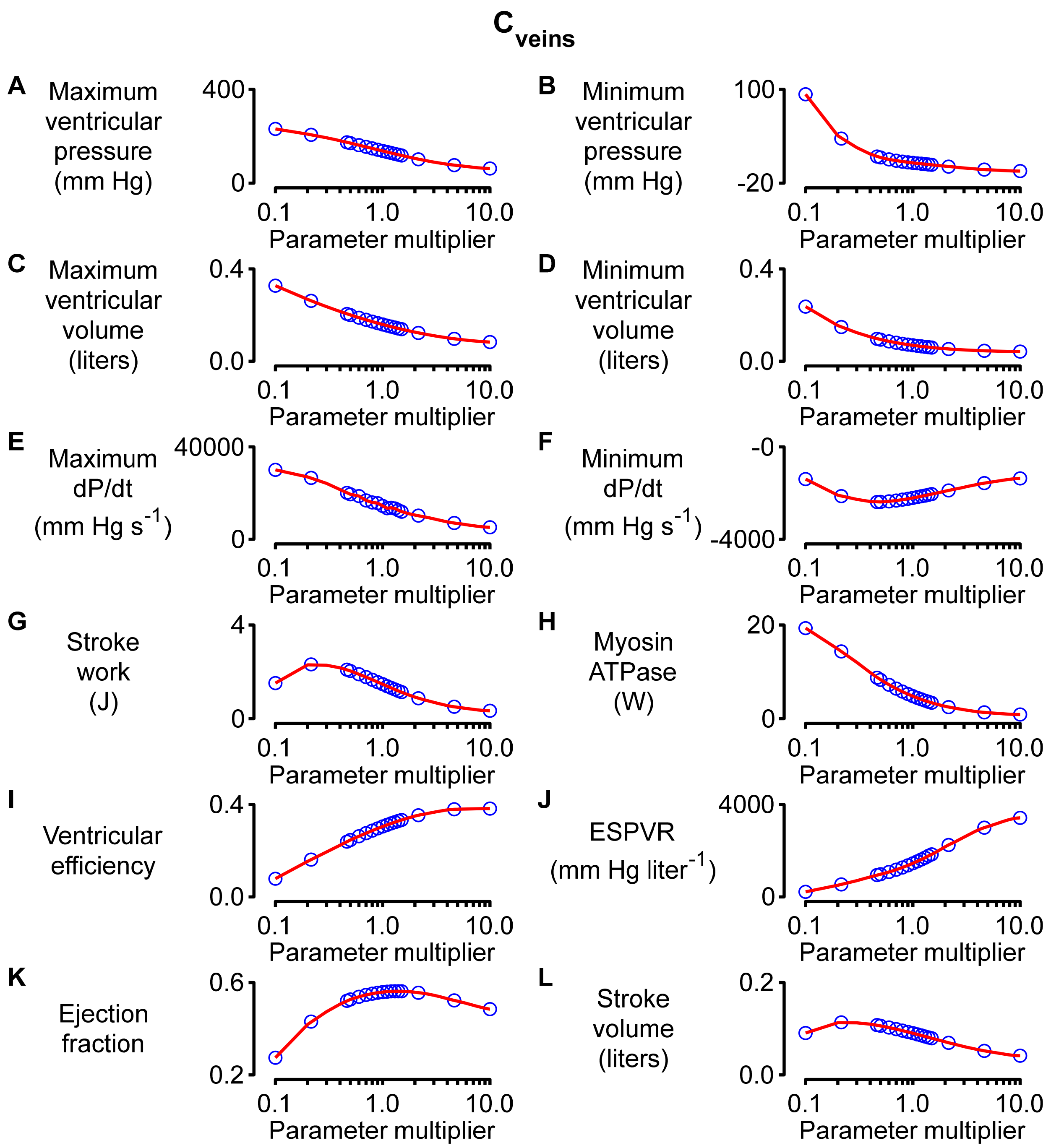

Figure S23: Effects of changing C_{veins} on system-level cardiovascular properties.

Panels A to L show values (blue circles) for 12 system-level properties (for example, maximum ventricular pressure) predicted for values of C_{veins} (see Methods in main text) ranging from 0.1 to 10 times the value shown in Table S1. The red lines shows the best-fit of a 5th order polynomial to the simulated data.

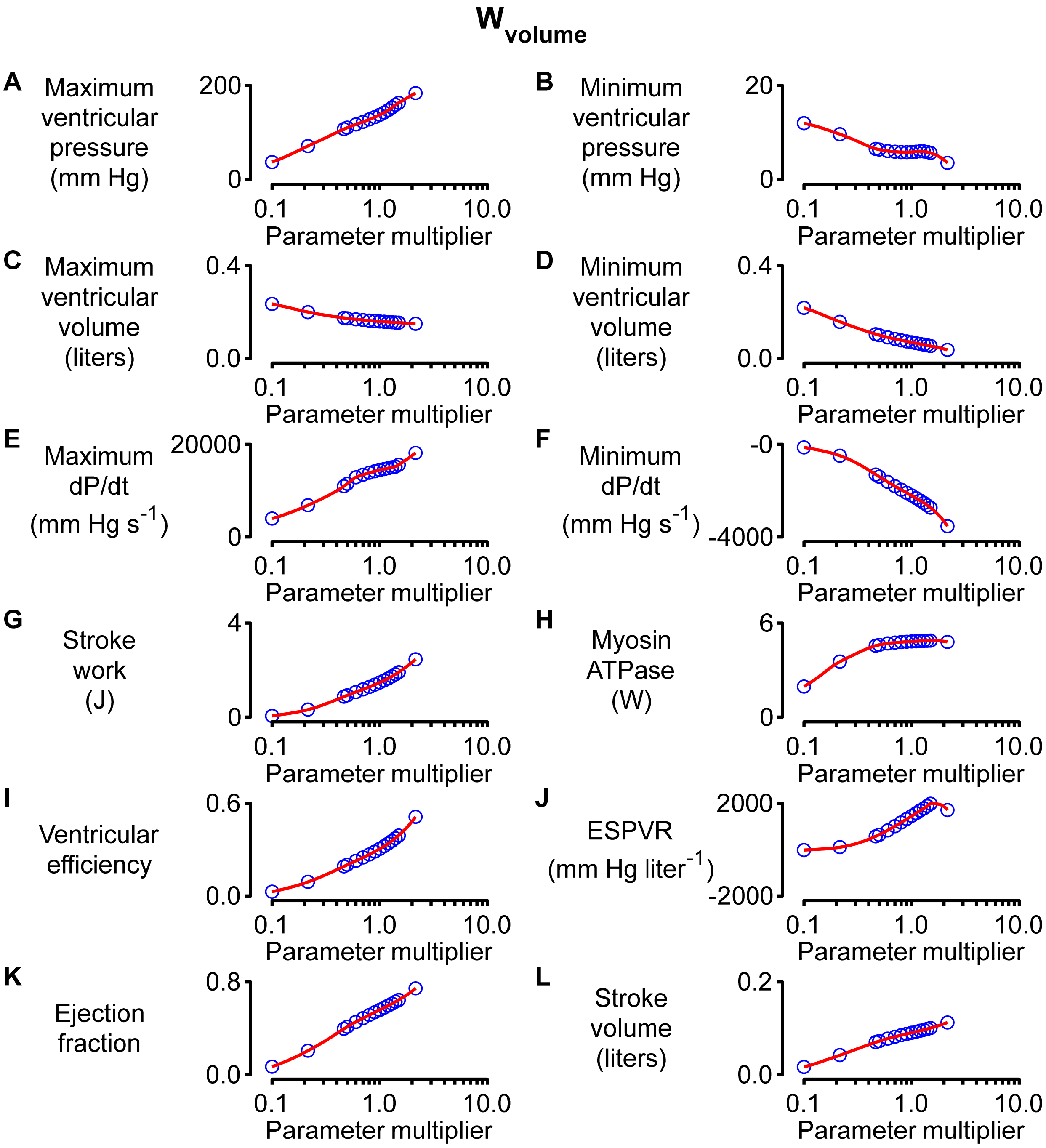

Figure S24: Effects of changing W_{volume} on system-level cardiovascular properties.

Panels A to L show values (blue circles) for 12 system-level properties (for example, maximum ventricular pressure) predicted for values of W_{volume} (see Methods in main text) ranging from 0.1 to 10 times the value shown in Table S1. The red lines shows the best-fit of a 5th order polynomial to the simulated data.

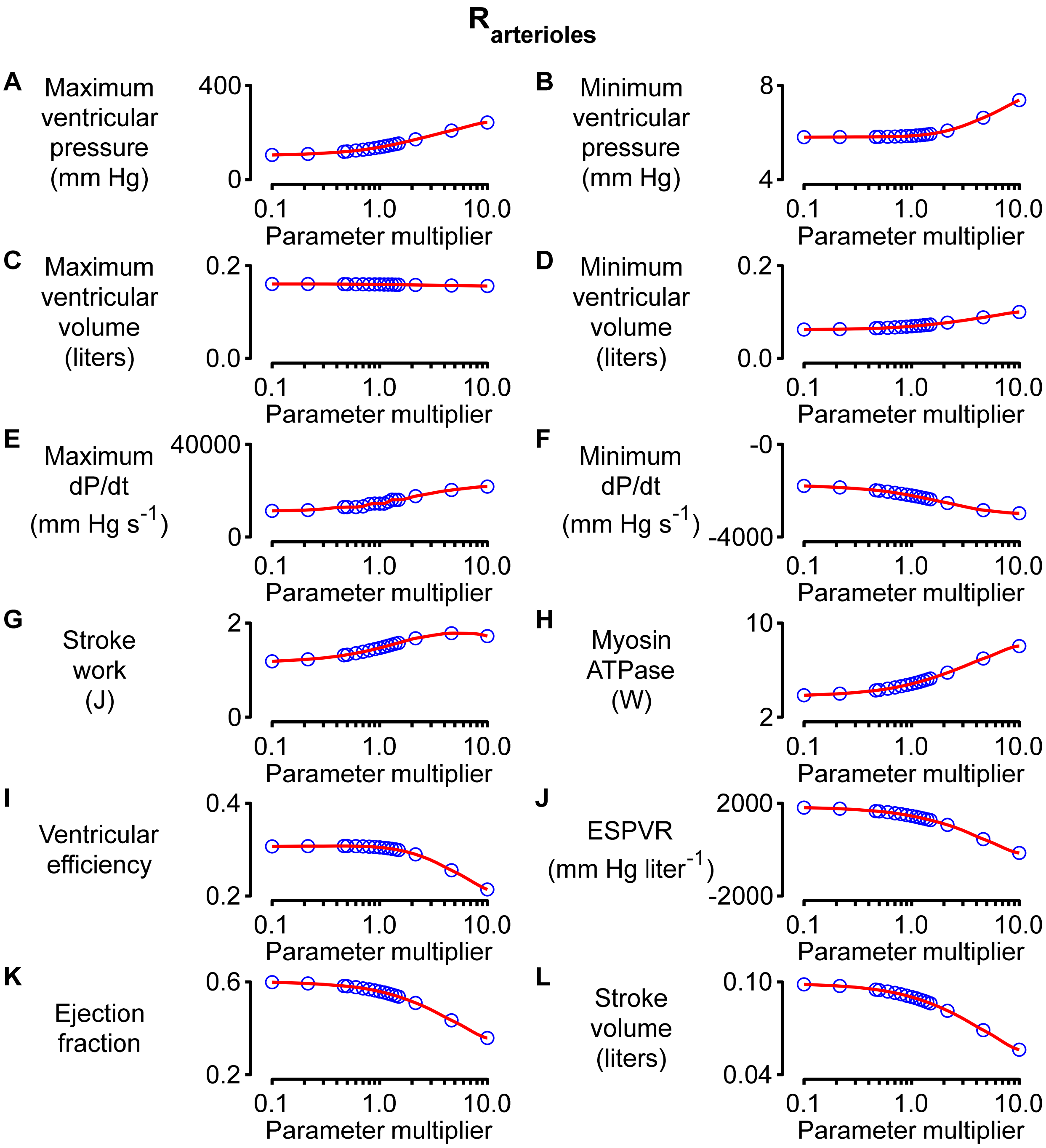

Figure S25: Effects of changing $R_{arterioles}$ on system-level cardiovascular properties.

Panels A to L show values (blue circles) for 12 system-level properties (for example, maximum ventricular pressure) predicted for values of $R_{arterioles}$ (see Methods in main text) ranging from 0.1 to 10 times the value shown in Table S1. The red lines shows the best-fit of a 5th order polynomial to the simulated data.

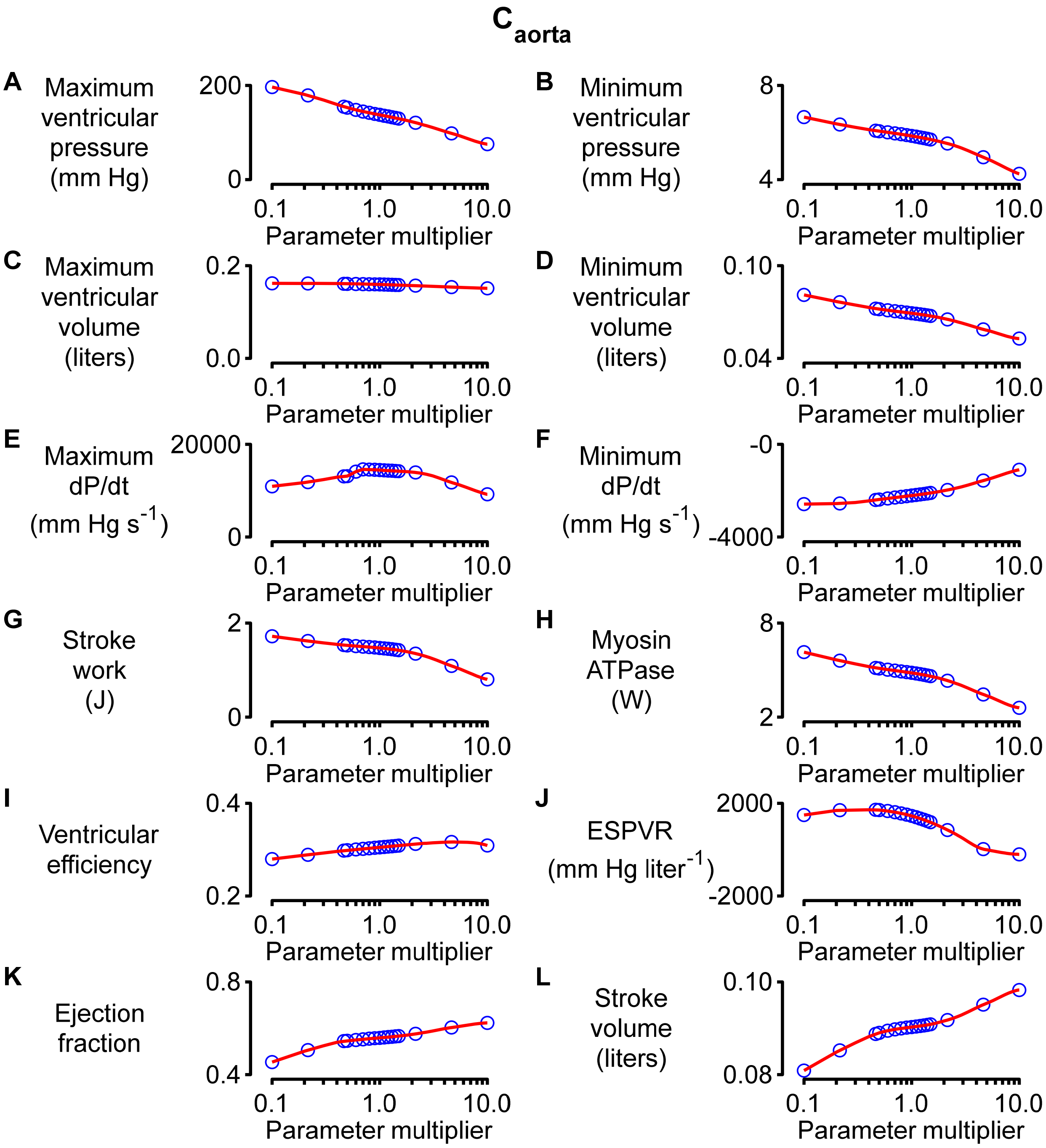

Figure S26: Effects of changing C_{aorta} on system-level cardiovascular properties.

Panels A to L show values (blue circles) for 12 system-level properties (for example, maximum ventricular pressure) predicted for values of C_{aorta} (see Methods in main text) ranging from 0.1 to 10 times the value shown in Table S1. The red lines shows the best-fit of a 5th order polynomial to the simulated data.

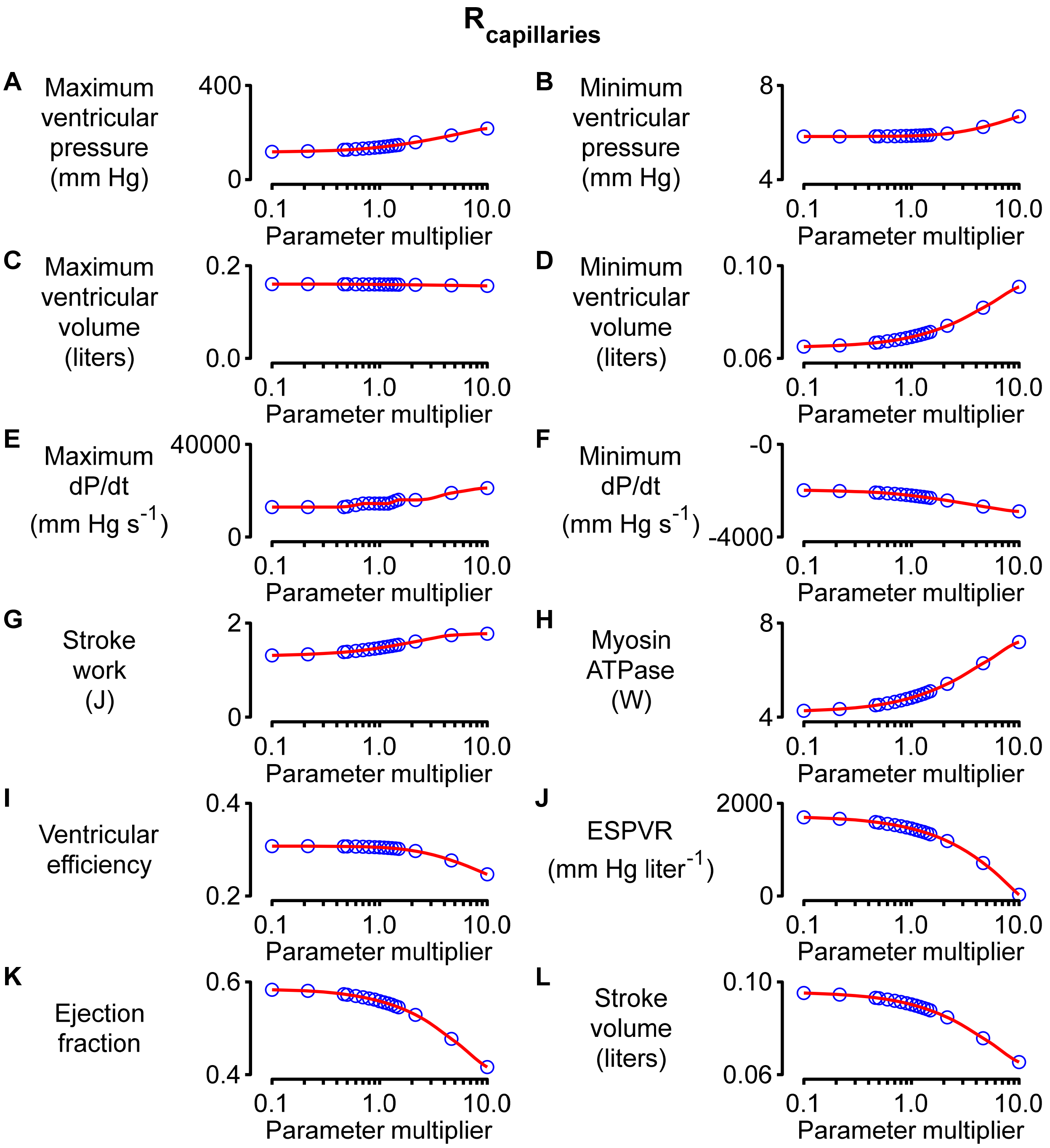

Figure S27: Effects of changing $R_{capillaries}$ on system-level cardiovascular properties.

Panels A to L show values (blue circles) for 12 system-level properties (for example, maximum ventricular pressure) predicted for values of $R_{capillaries}$ (see Methods in main text) ranging from 0.1 to 10 times the value shown in Table S1. The red lines shows the best-fit of a 5th order polynomial to the simulated data.

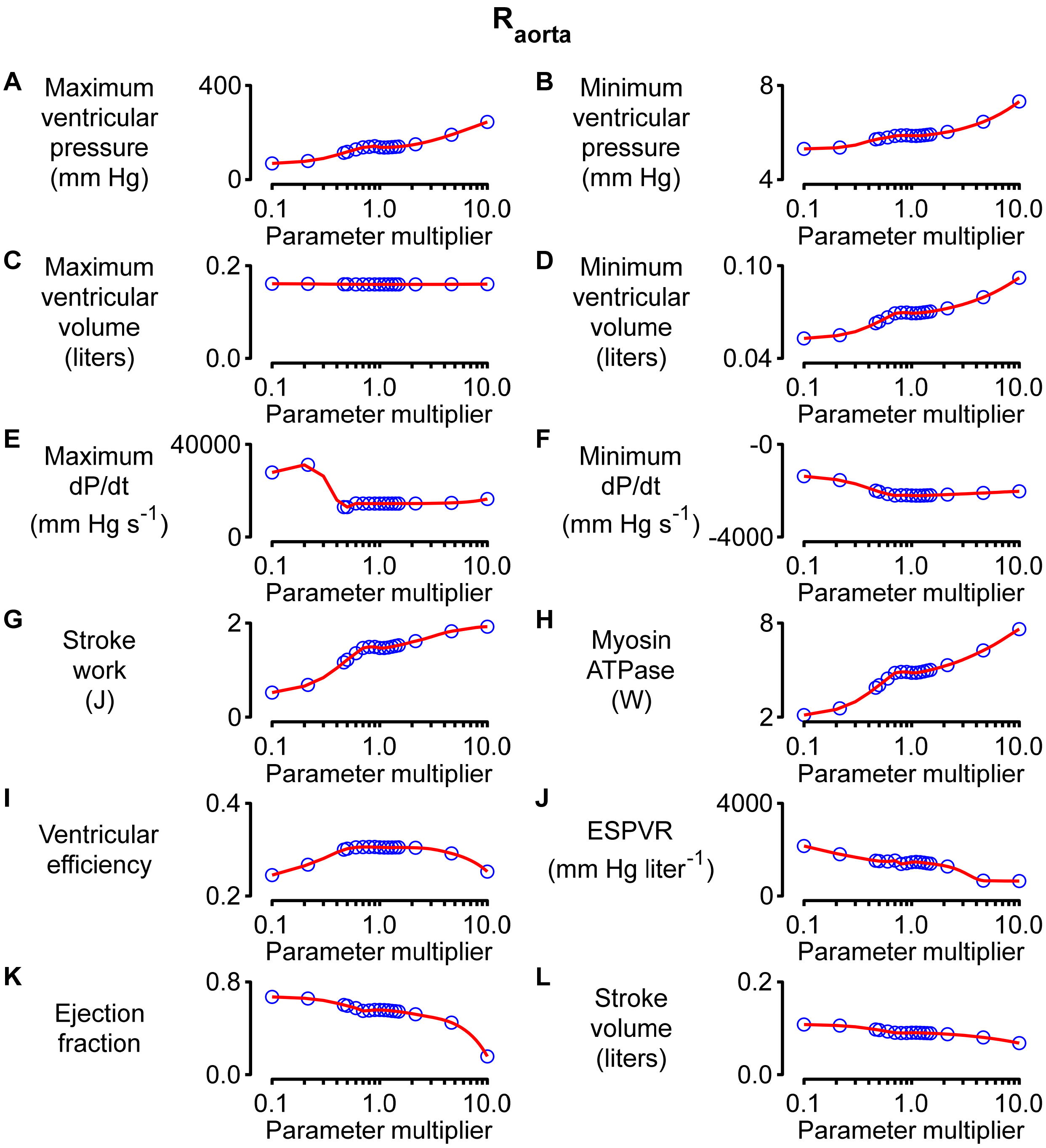

Figure S28: Effects of changing R_{aorta} on system-level cardiovascular properties.

Panels A to L show values (blue circles) for 12 system-level properties (for example, maximum ventricular pressure) predicted for values of R_{aorta} (see Methods in main text) ranging from 0.1 to 10 times the value shown in Table S1. The red lines shows the best-fit of a 5th order polynomial to the simulated data.

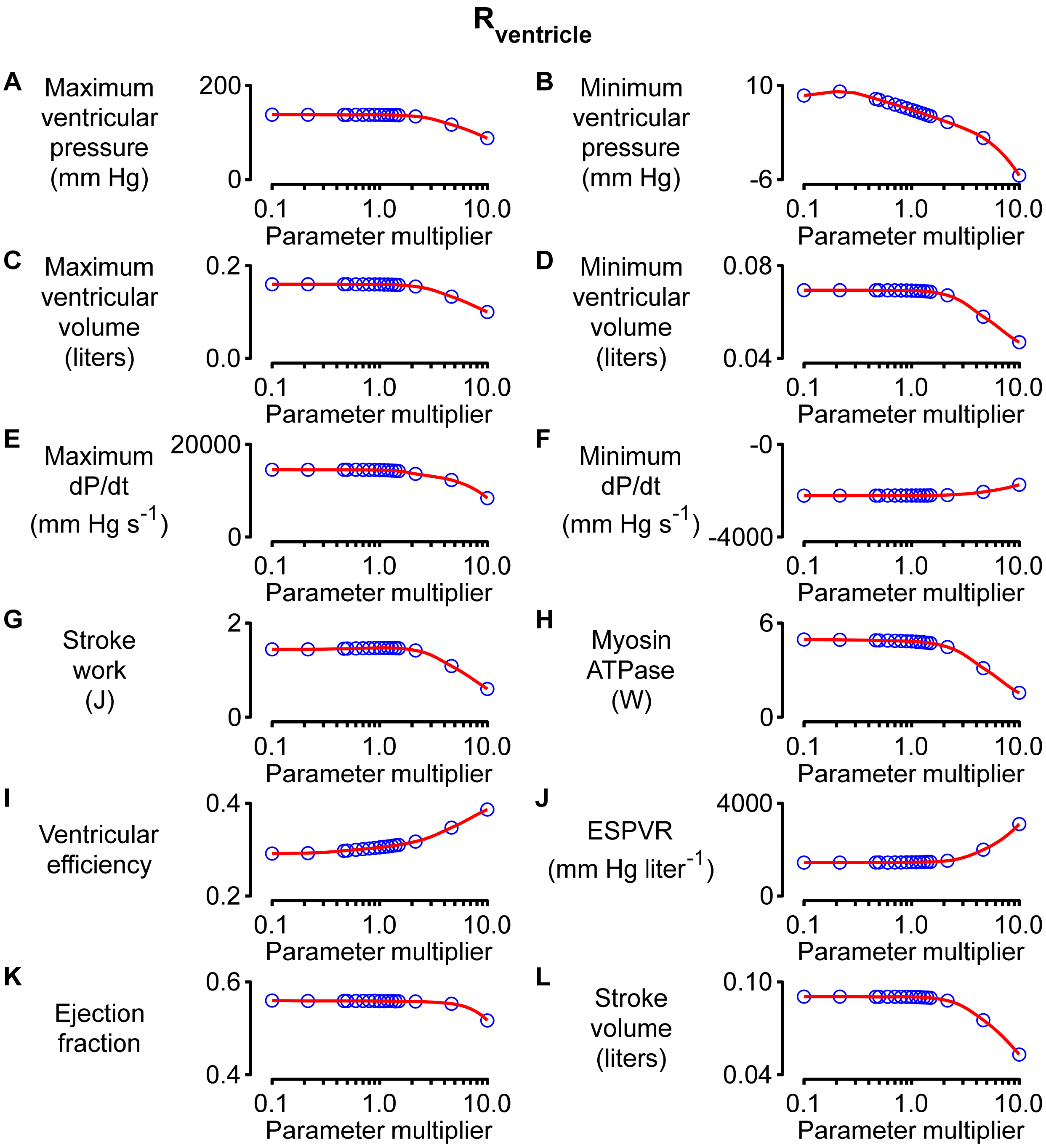

Figure S29: Effects of changing $R_{ventricle}$ on system-level cardiovascular properties.

Panels A to L show values (blue circles) for 12 system-level properties (for example, maximum ventricular pressure) predicted for values of $R_{ventricle}$ (see Methods in main text) ranging from 0.1 to 10 times the value shown in Table S1. The red lines shows the best-fit of a 5th order polynomial to the simulated data.

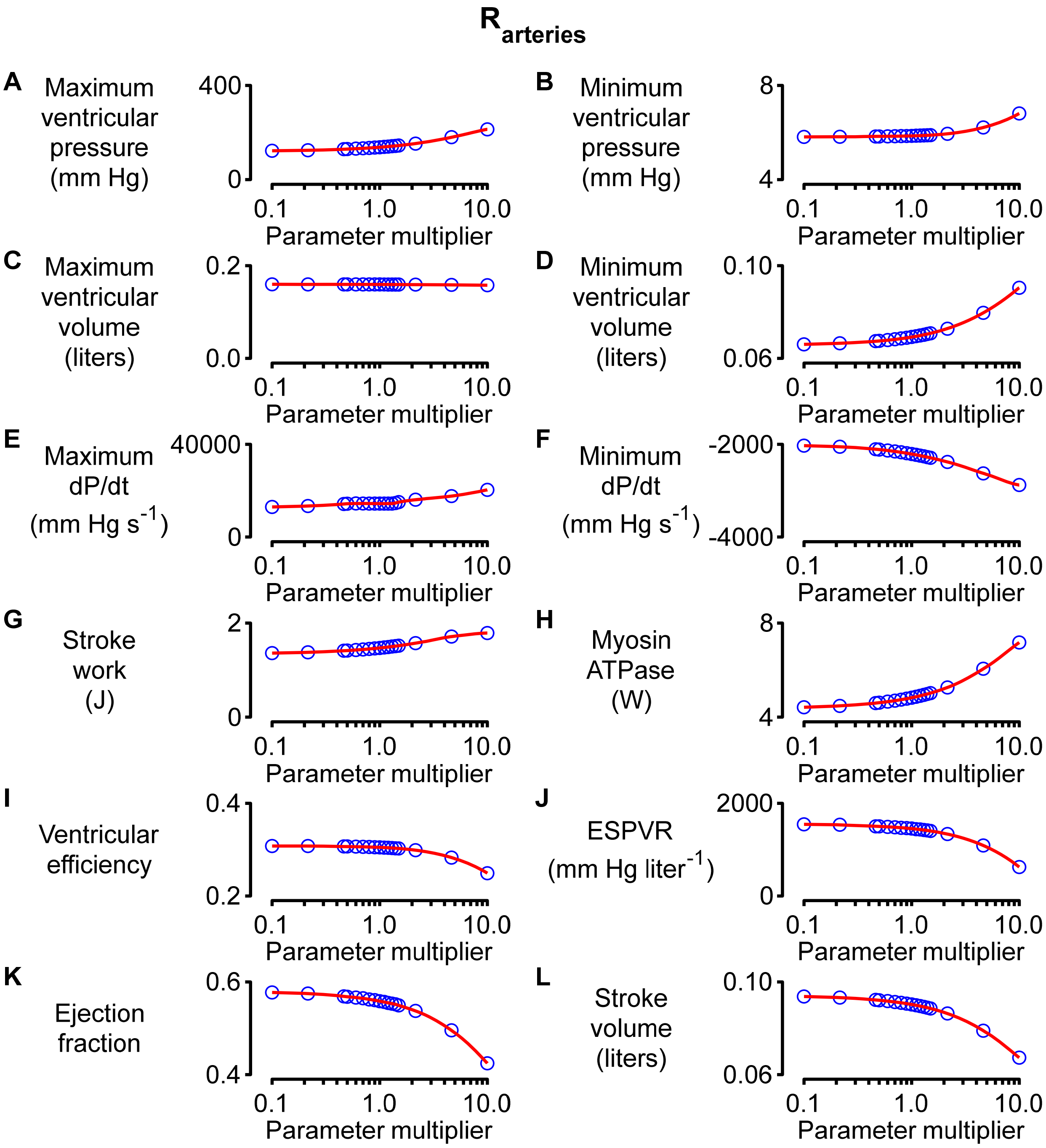

Figure S30: Effects of changing $R_{arteries}$ on system-level cardiovascular properties.

Panels A to L show values (blue circles) for 12 system-level properties (for example, maximum ventricular pressure) predicted for values of $R_{arteries}$ (see Methods in main text) ranging from 0.1 to 10 times the value shown in Table S1. The red lines shows the best-fit of a 5th order polynomial to the simulated data.

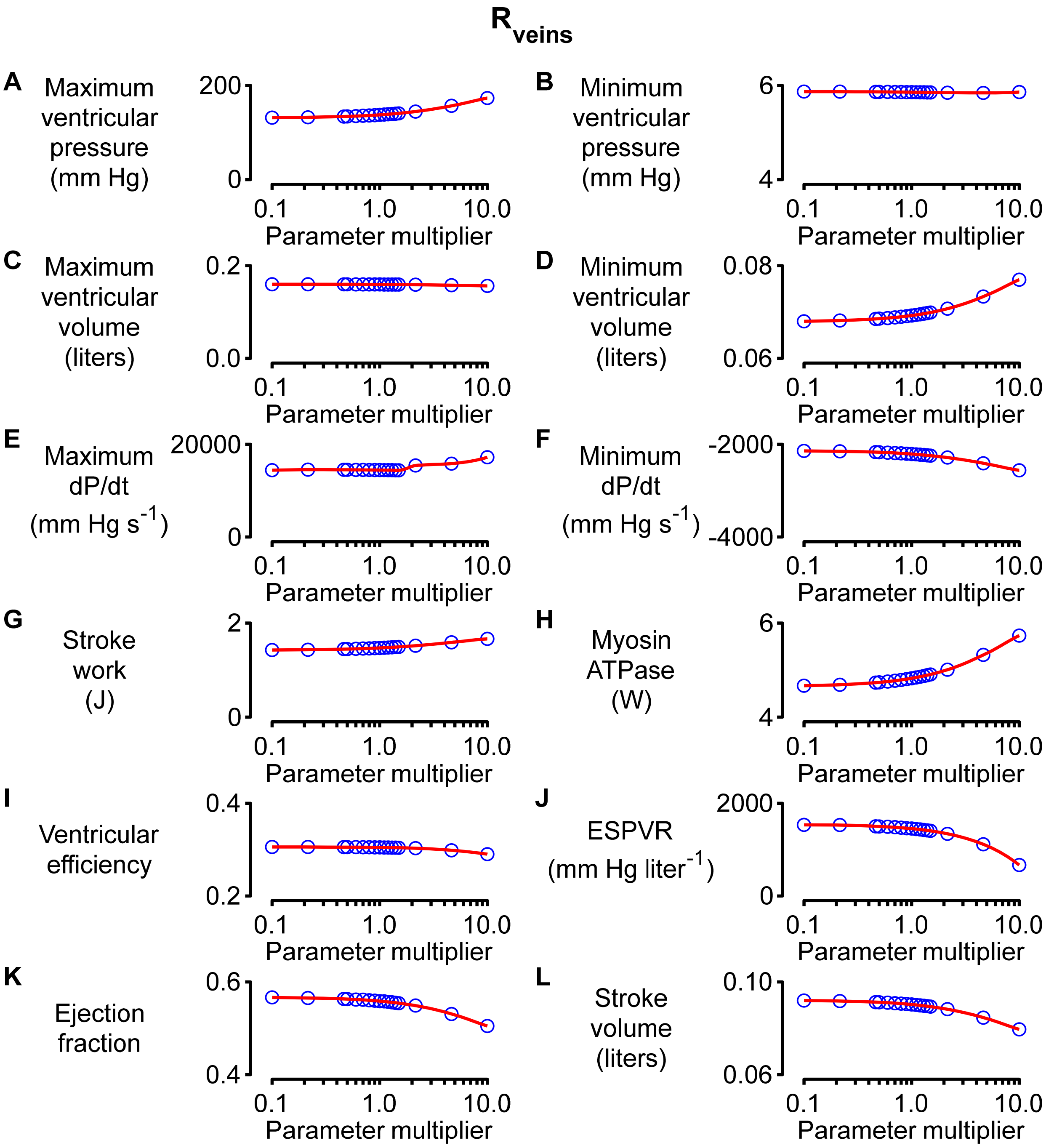

Figure S31: Effects of changing R_{veins} on system-level cardiovascular properties.

Panels A to L show values (blue circles) for 12 system-level properties (for example, maximum ventricular pressure) predicted for values of R_{veins} (see Methods in main text) ranging from 0.1 to 10 times the value shown in Table S1. The red lines shows the best-fit of a 5th order polynomial to the simulated data.

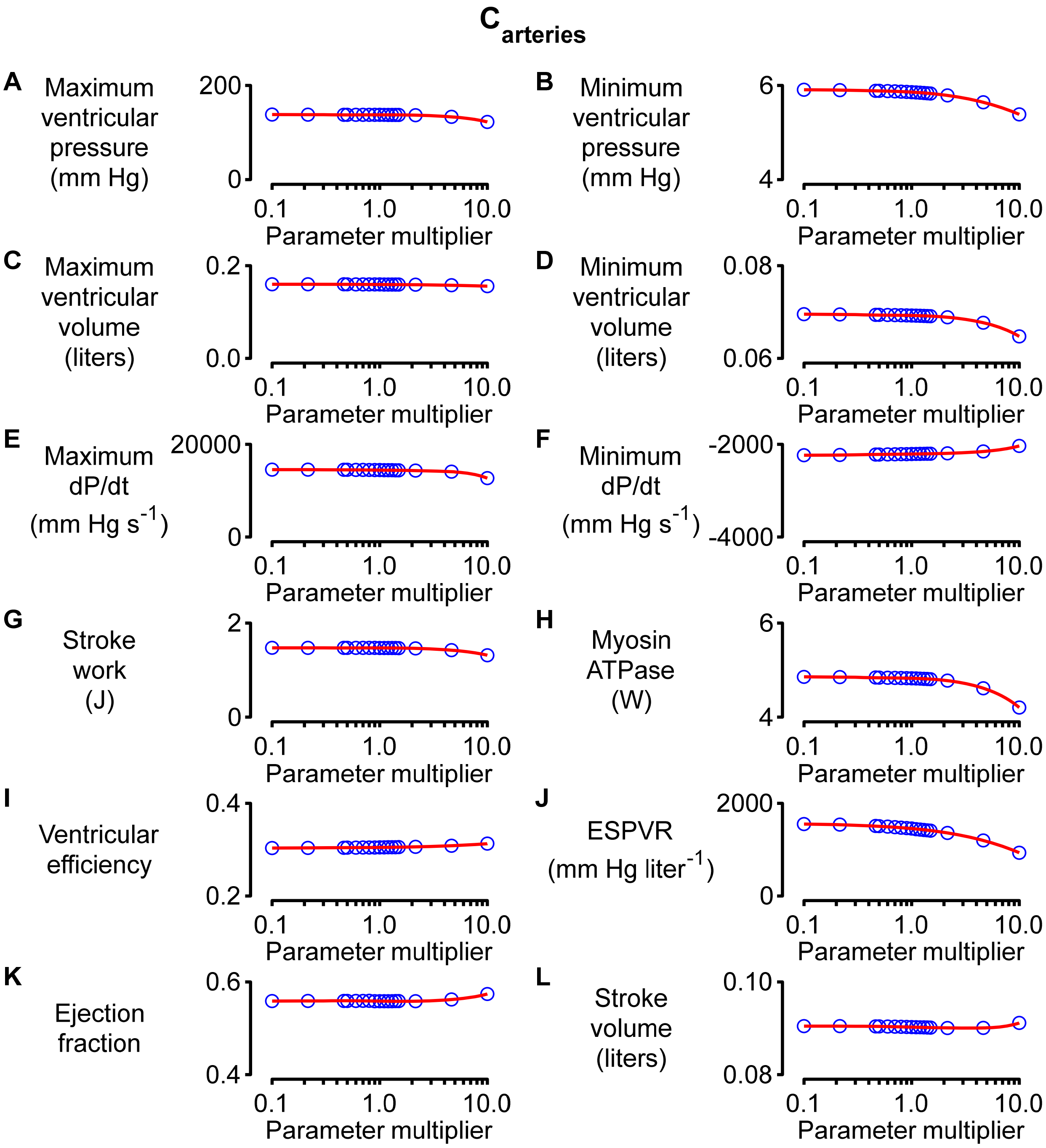

Figure S32: Effects of changing $C_{arteries}$ on system-level cardiovascular properties.

Panels A to L show values (blue circles) for 12 system-level properties (for example, maximum ventricular pressure) predicted for values of $C_{arteries}$ (see Methods in main text) ranging from 0.1 to 10 times the value shown in Table S1. The red lines shows the best-fit of a 5th order polynomial to the simulated data.

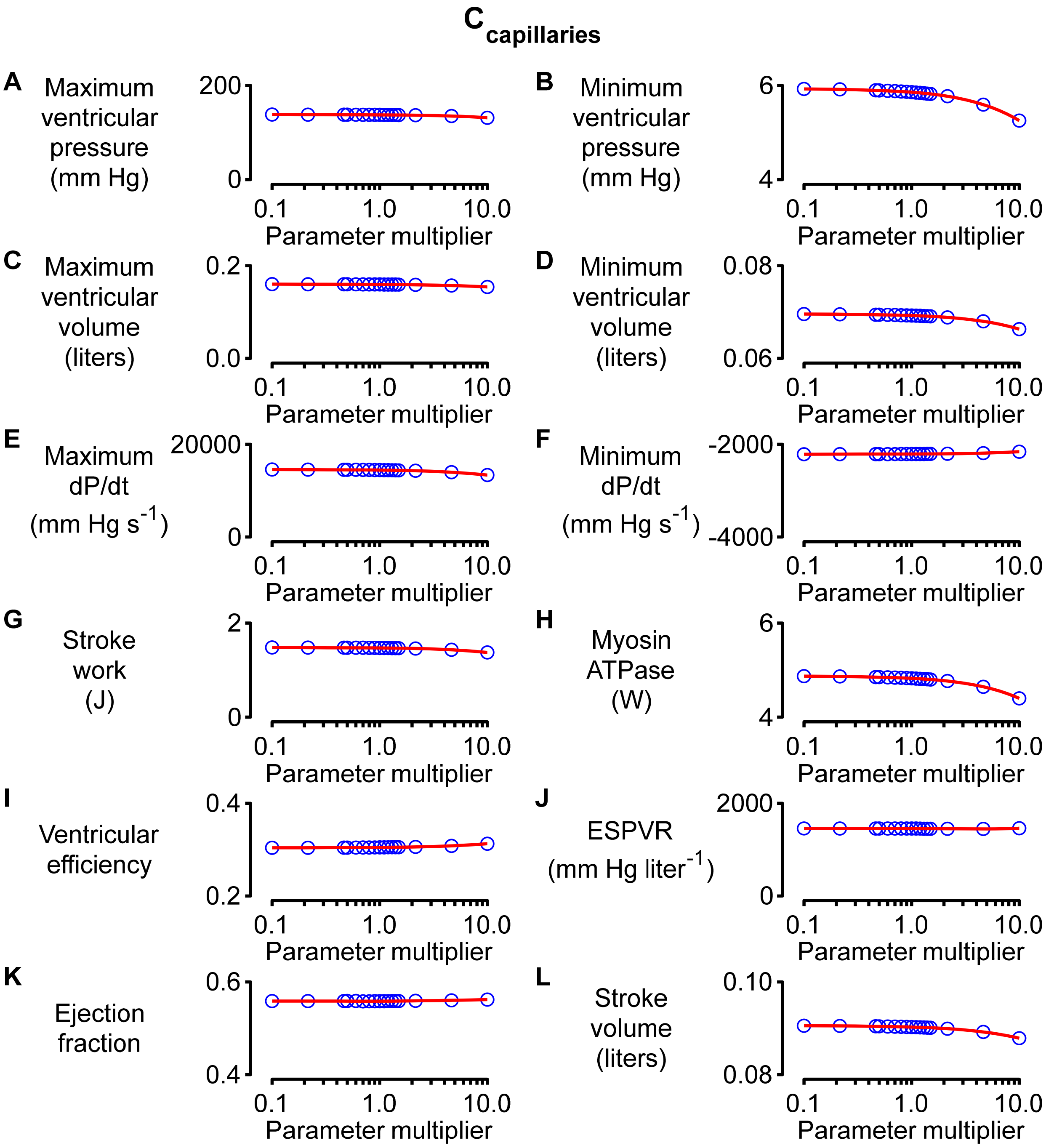

Figure S33: Effects of changing $C_{capillaries}$ on system-level cardiovascular properties.

Panels A to L show values (blue circles) for 12 system-level properties (for example, maximum ventricular pressure) predicted for values of $C_{capillaries}$ (see Methods in main text) ranging from 0.1 to 10 times the value shown in Table S1. The red lines shows the best-fit of a 5th order polynomial to the simulated data.

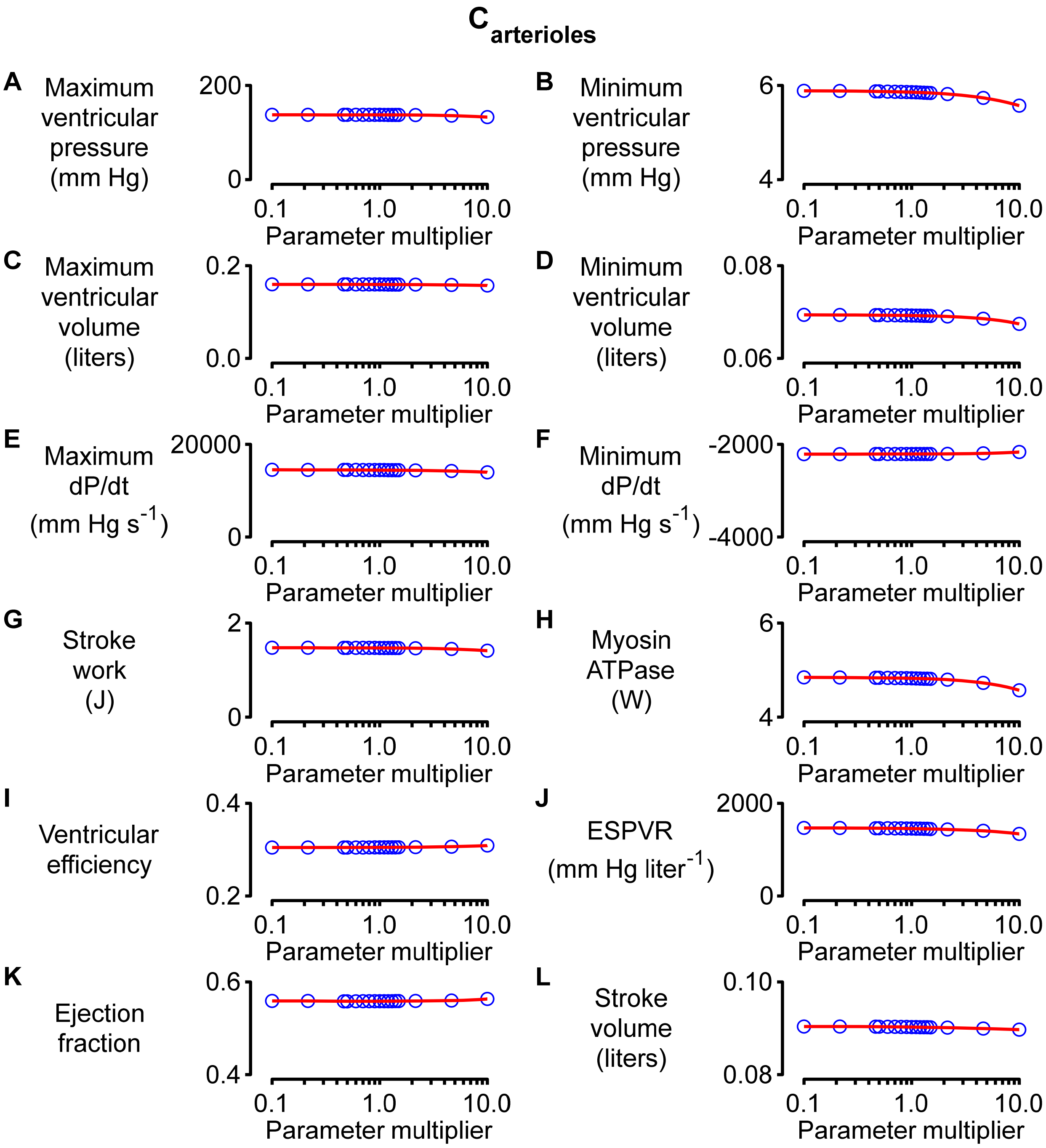

Figure S34: Effects of changing $C_{arterioles}$ on system-level cardiovascular properties.

Panels A to L show values (blue circles) for 12 system-level properties (for example, maximum ventricular pressure) predicted for values of $C_{arterioles}$ (see Methods in main text) ranging from 0.1 to 10 times the value shown in Table S1. The red lines shows the best-fit of a 5th order polynomial to the simulated data.

Constrained passive stress

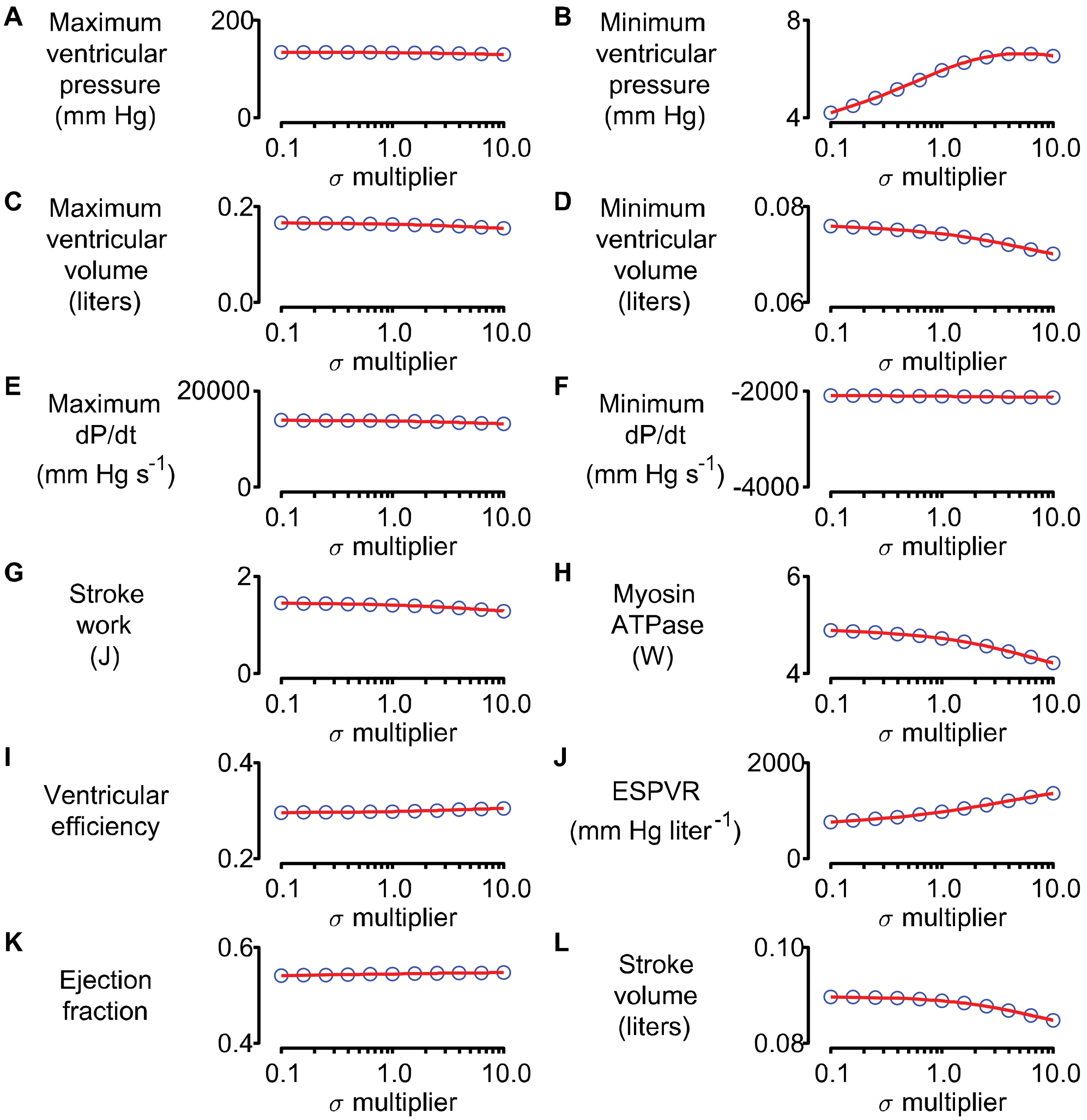

Figure S35: Effects of changing σ while constraining passive stress.

Panels A to L show values (blue circles) for 12 system-level properties (for example, maximum ventricular pressure) predicted for different combinations of σ and L (see equation S9) so that passive stress at a half-sarcomere length of 1069 nm was held constant. Other values as shown in Table S1. The red lines shows the best-fit of a 5th order polynomial to the simulated data.

Ait-Mou, Y., Hsu, K., Farman, G.P., Kumar, M., Greaser, M.L., Irving, T.C., and De Tombe, P.P. (2016). Titin strain contributes to the Frank-Starling law of the heart by structural rearrangements of both thin- and thick-filament proteins. *Proc Natl Acad Sci U S A* 113**,** 2306-2311.

Alamo, L., Wriggers, W., Pinto, A., Bartoli, F., Salazar, L., Zhao, F.Q., Craig, R., and Padron, R. (2008). Three-dimensional reconstruction of tarantula myosin filaments suggests how phosphorylation may regulate myosin activity. *J Mol Biol* 384**,** 780-797.

Bagnoli, P., Malagutti, N., Gastaldi, D., Marcelli, E., Lui, E., Cercenelli, L., Costantino, M.L., Plicchi, G., and Fumero, R. (2011). Computational finite element model of cardiac torsion. *Int J Artif Organs* 34**,** 44-53.

Benjamin, E.J., Virani, S.S., Callaway, C.W., Chamberlain, A.M., Chang, A.R., Cheng, S., Chiuve, S.E., Cushman, M., Delling, F.N., Deo, R., De Ferranti, S.D., Ferguson, J.F., Fornage, M., Gillespie, C., Isasi, C.R., Jimenez, M.C., Jordan, L.C., Judd, S.E., Lackland, D., Lichtman, J.H., Lisabeth, L., Liu, S., Longenecker, C.T., Lutsey, P.L., Mackey, J.S., Matchar, D.B., Matsushita, K., Mussolino, M.E., Nasir, K., O'flaherty, M., Palaniappan, L.P., Pandey, A., Pandey, D.K., Reeves, M.J., Ritchey, M.D., Rodriguez, C.J., Roth, G.A., Rosamond, W.D., Sampson, U.K.A., Satou, G.M., Shah, S.H., Spartano, N.L., Tirschwell, D.L., Tsao, C.W., Voeks, J.H., Willey, J.Z., Wilkins, J.T., Wu, J.H., Alger, H.M., Wong, S.S., Muntner, P., American Heart Association Council On, E., Prevention Statistics, C., and Stroke Statistics, S. (2018). Heart Disease and Stroke Statistics-2018 Update: A Report From the American Heart Association. *Circulation* 137**,** e67-e492.

Bodi, I., Mikala, G., Koch, S.E., Akhter, S.A., and Schwartz, A. (2005). The L-type calcium channel in the heart: the beat goes on. *J Clin Invest* 115**,** 3306-3317.

Campbell, K.B., Simpson, A.M., Campbell, S.G., Granzier, H.L., and Slinker, B.K. (2008). Dynamic left ventricular elastance: a model for integrating cardiac muscle contraction into ventricular pressure-volume relationships. *J Appl Physiol (1985)* 104**,** 958-975.

Campbell, K.S. (2009). Interactions between connected half-sarcomeres produce emergent behavior in a mathematical model of muscle. *PLOS Computational Biology* 5**,** e1000560. doi:1000510.1001371/journal.pcbi.1000560.

Campbell, K.S. (2011). Impact of myocyte strain on cardiac myofilament activation. *Pflugers Arch* 462**,** 3-14.

Campbell, K.S. (2014). Dynamic coupling of regulated binding sites and cycling myosin heads in striated muscle. *J Gen Physiol* 143**,** 387-399.

Campbell, K.S., Janssen, P.M.L., and Campbell, S.G. (2018). Force-Dependent Recruitment from the Myosin Off State Contributes to Length-Dependent Activation. *Biophys J* 115**,** 543-553.

Campbell, K.S., and Lakie, M. (1998). A cross-bridge mechanism can explain the thixotropic short-range elastic component of relaxed frog skeletal muscle. *Journal of Physiology* 510.3**,** 941-962.

Campbell, K.S., Yengo, C.M., Lee, L.C., Kotter, J., Sorrell, V.L., Guglin, M., and Wenk, J.F. (2019). Closing the therapeutic loop. *Arch Biochem Biophys* 663**,** 129-131.

Getz, E.B., Cooke, R., and Lehman, S.L. (1998). Phase transition in force during ramp stretches of skeletal muscle. *Biophysical Journal* 75**,** 2971-2983.

Granzier, H.L., and Irving, T.C. (1995). Passive tension in cardiac muscles: contribution of collagen, titin, microtubules, and intermediate filaments. *Biophysical Journal* 68**,** 1027-1044.

Guccione, J.M., Costa, K.D., and Mcculloch, A.D. (1995). Finite element stress analysis of left ventricular mechanics in the beating dog heart. *J Biomech* 28**,** 1167-1177.

Haynes, P., Nava, K.E., Lawson, B.A., Chung, C.S., Mitov, M.I., Campbell, S.G., Stromberg, A.J., Sadayappan, S., Bonnell, M.R., Hoopes, C.W., and Campbell, K.S. (2014). Transmural heterogeneity of cellular level power output is reduced in human heart failure. *J Mol Cell Cardiol* 72**,** 1-8.

Hidalgo, C., and Granzier, H. (2013). Tuning the molecular giant titin through phosphorylation: role in health and disease. *Trends Cardiovasc Med* 23**,** 165-171.

Hooijman, P., Stewart, M.A., and Cooke, R. (2011). A new state of cardiac myosin with very slow ATP turnover: a potential cardioprotective mechanism in the heart. *Biophys J* 100**,** 1969-1976.

Houston, B.A., and Stevens, G.R. (2014). Hypertrophic cardiomyopathy: a review. *Clin Med Insights Cardiol* 8**,** 53-65.

Huxley, H.E., Stewart, A., Sosa, H., and Irving, T. (1994). X-ray diffraction measurements of the extensibility of actin and myosin filaments in contracting muscle. *Biophysical Journal* 67**,** 2411-2421.

Irving, M. (2017). Regulation of Contraction by the Thick Filaments in Skeletal Muscle. *Biophys J* 113, 2579-2594.

Jones, E., Oliphant, T.E., and Peterson, P. (2001-). *SciPy: Open source scientific tools for Python* [Online]. Available: http://www.scipy.org [Accessed 11 August 2019].

Kampourakis, T., Sun, Y.B., and Irving, M. (2016). Myosin light chain phosphorylation enhances contraction of heart muscle via structural changes in both thick and thin filaments. *Proc Natl Acad Sci U S A* 113, E3039-3047.

Klepach, D., Lee, L.C., Wenk, J.F., Ratcliffe, M.B., Zohdi, T.I., Navia, J.A., Kassab, G.S., Kuhl, E., and Guccione, J.M. (2012). Growth and remodeling of the left ventricle: A case study of myocardial infarction and surgical ventricular restoration. *Mech Res Commun* 42, 134-141.

Kosinski, S.A., Carlson, B.E., Hummel, S.L., Brook, R.D., and Beard, D.A. (2018). Computational model-based assessment of baroreflex function from response to Valsalva maneuver. *J Appl Physiol (1985)* 125, 1944-1967.

Lee, L.C., Sundnes, J., Genet, M., Wenk, J.F., and Wall, S.T. (2016). An integrated electromechanical-growth heart model for simulating cardiac therapies. *Biomech Model Mechanobiol* 15, 791-803.

Lewinter, M.M., and Granzier, H.L. (2014). Cardiac titin and heart disease. *J Cardiovasc Pharmacol* 63, 207-212.

Linari, M., Caremani, M., Piperio, C., Brandt, P., and Lombardi, V. (2007). Stiffness and fraction of myosin motors responsible for active force in permeabilized muscle fibers from rabbit psoas. *Biophysical Journal* 92, 2476-2490.

Lloyd, C.M., Lawson, J.R., Hunter, P.J., and Nielsen, P.F. (2008). The CellML Model Repository. *Bioinformatics* 24, 2122-2123.

Lumens, J., Delhaas, T., Kirn, B., and Arts, T. (2009). Three-wall segment (TriSeg) model describing mechanics and hemodynamics of ventricular interaction. *Ann Biomed Eng* 37, 2234-2255.

Malik, F.I., Hartman, J.J., Elias, K.A., Morgan, B.P., Rodriguez, H., Brejc, K., Anderson, R.L., Sueoka, S.H., Lee, K.H., Finer, J.T., Sakowicz, R., Baliga, R., Cox, D.R., Garard, M., Godinez, G., Kawas, R., Kraynack, E., Lenzi, D., Lu, P.P., Muci, A., Niu, C., Qian, X., Pierce, D.W., Pokrovskii, M., Suehiro, I., Sylvester, S., Tochimoto, T., Valdez, C., Wang, W., Katori, T., Kass, D.A., Shen, Y.T., Vatner, S.F., and Morgans, D.J. (2011). Cardiac myosin activation: a potential therapeutic approach for systolic heart failure. *Science* 331, 1439-1443.

Mann, C.K., Lee, L.C., Campbell, K.S., and Wenk, J.F. (2020). Force-dependent recruitment from myosin OFF-state increases end-systolic pressure-volume relationship in left ventricle. *Biomech Model Mechanobiol*.

Marian, A.J., and Braunwald, E. (2017). Hypertrophic Cardiomyopathy: Genetics, Pathogenesis, Clinical Manifestations, Diagnosis, and Therapy. *Circ Res* 121, 749-770.

Mirsky, I., Tajimi, T., and Peterson, K.L. (1987). The development of the entire end-systolic pressure-volume and ejection fraction-afterload relations: a new concept of systolic myocardial stiffness. *Circulation* 76, 343-356.

Nag, S., Trivedi, D.V., Sarkar, S.S., Adhikari, A.S., Sunitha, M.S., Sutton, S., Ruppel, K.M., and Spudich, J.A. (2017). The myosin mesa and the basis of hypercontractility caused by hypertrophic cardiomyopathy mutations. *Nat Struct Mol Biol* 24, 525-533.

Nagueh, S.F., Shah, G., Wu, Y., Torre-Amione, G., King, N.M., Lahmers, S., Witt, C.C., Becker, K., Labeit, S., and Granzier, H.L. (2004). Altered titin expression, myocardial stiffness, and left ventricular function in patients with dilated cardiomyopathy. *Circulation* 110, 155-162.

Negroni, J.A., and Lascano, E.C. (1996). A cardiac muscle model relating sarcomere dynamics to calcium kinetics. *Journal of Molecular and Cellular Cardiology* 28, 915-929.

Parmley, W.W. (1985). Pathophysiology of congestive heart failure. *Am J Cardiol* 56, 7A-11A.

Pironet, A., Desaive, T., Kosta, S., Lucas, A., Paeme, S., Collet, A., Pretty, C.G., Kolh, P., and Dauby, P.C. (2013). A multi-scale cardiovascular system model can account for the load-dependence of the end-systolic pressure-volume relationship. *Biomed Eng Online* 12, 8.

Russel, I.K., Gotte, M.J., Bronzwaer, J.G., Knaapen, P., Paulus, W.J., and Van Rossum, A.C. (2009). Left ventricular torsion: an expanding role in the analysis of myocardial dysfunction. *JACC Cardiovasc Imaging* 2, 648-655.

Sharma, S., Razeghi, P., Shakir, A., Keneson, B.J., 2nd, Clubb, F., and Taegtmeyer, H. (2003). Regional heterogeneity in gene expression profiles: a transcript analysis in human and rat heart. *Cardiology* 100, 73-79.

Shi, Y., Lawford, P., and Hose, R. (2011). Review of zero-D and 1-D models of blood flow in the cardiovascular system. *Biomed Eng Online* 10**,** 33.

Shim, E., Leem, C., and Youn, C. (2004). *System biological modeling of circulation: From cells to system.*

Sobol, I.M. (1993). Sensitivity analysis for non-linear mathematical models. *Mathematical Modeling and Computational Experiments* 1**,** 407-414.

Streeter, D.D., Jr., Spotnitz, H.M., Patel, D.P., Ross, J., Jr., and Sonnenblick, E.H. (1969). Fiber orientation in the canine left ventricle during diastole and systole. *Circ Res* 24**,** 339-347.

Swenson, A.M., Tang, W., Blair, C.A., Fetrow, C.M., Unrath, W.C., Previs, M.J., Campbell, K.S., and Yengo, C.M. (2017). Omecamtiv Mecarbil Enhances the Duty Ratio of Human beta-Cardiac Myosin Resulting in Increased Calcium Sensitivity and Slowed Force Development in Cardiac Muscle. *J Biol Chem* 292**,** 3768-3778.

Ten Tusscher, K.H., Noble, D., Noble, P.J., and Panfilov, A.V. (2004). A model for human ventricular tissue. *Am J Physiol Heart Circ Physiol* 286**,** H1573-1589.

Ter Keurs, H.E., and Boyden, P.A. (2007). Calcium and arrhythmogenesis. *Physiol Rev* 87**,** 457-506.

Ter Keurs, H.E., Wakayama, Y., Miura, M., Shinozaki, T., Stuyvers, B.D., Boyden, P.A., and Landesberg, A. (2006). Arrhythmogenic Ca2+ release from cardiac myofilaments. *Prog Biophys Mol Biol* 90**,** 151-171.

Tewari, S.G., Bugenhagen, S.M., Vinnakota, K.C., Rice, J.J., Janssen, P.M., and Beard, D.A. (2016). Influence of metabolic dysfunction on cardiac mechanics in decompensated hypertrophy and heart failure. *J Mol Cell Cardiol* 94**,** 162-175.

Van Der Walt, S., Colbert, S.C., and Varoquaux, G. (2011). The NumPy Array: A structure for efficient numerical computation. *Computing in Science and Engineering* 13**,** 22-30.

Vonk Noordegraaf, A., Westerhof, B.E., and Westerhof, N. (2017). The Relationship Between the Right Ventricle and its Load in Pulmonary Hypertension. *J Am Coll Cardiol* 69**,** 236-243.

Wakabayashi, K., Sugimoto, Y., Tanaka, H., Ueno, Y., Takezawa, Y., and Amemiya, Y. (1994). X-ray diffraction evidence for the extensibility of actin and myosin filaments during muscle contraction. *Biophysical Journal* 67**,** 2422-2435.

Wakayama, Y., Miura, M., Sugai, Y., Kagaya, Y., Watanabe, J., Ter Keurs, H.E., and Shirato, K. (2001). Stretch and quick release of rat cardiac trabeculae accelerates Ca2+ waves and triggered propagated contractions. *Am J Physiol Heart Circ Physiol* 281**,** H2133-2142.

Wang, H., Zhang, X., Dorsey, S.M., Mcgarvey, J.R., Campbell, K.S., Burdick, J.A., Gorman, J.H., 3rd, Pilla, J.J., Gorman, R.C., and Wenk, J.F. (2016). Computational Investigation of Transmural Differences in Left Ventricular Contractility. *J Biomech Eng* 138.

Watkins, H., Ashrafian, H., and Redwood, C. (2011). Inherited cardiomyopathies. *N Engl J Med* 364**,** 1643-1656.

Wenk, J.F. (2011). Numerical modeling of stress in stenotic arteries with microcalcifications: a parameter sensitivity study. *J Biomech Eng* 133**,** 014503.

Writing Committee, M., Yancy, C.W., Jessup, M., Bozkurt, B., Butler, J., Casey, D.E., Jr., Drazner, M.H., Fonarow, G.C., Geraci, S.A., Horwich, T., Januzzi, J.L., Johnson, M.R., Kasper, E.K., Levy, W.C., Masoudi, F.A., Mcbride, P.E., Mcmurray, J.J., Mitchell, J.E., Peterson, P.N., Riegel, B., Sam, F., Stevenson, L.W., Tang, W.H., Tsai, E.J., Wilkoff, B.L., and American College of Cardiology Foundation/American Heart Association Task Force on Practice, G. (2013). 2013 ACCF/AHA guideline for the management of heart failure: a report of the American College of Cardiology Foundation/American Heart Association Task Force on practice guidelines. *Circulation* 128**,** e240-327.

Zhang, X., Kampourakis, T., Yan, Z., Sevrieva, I., Irving, M., and Sun, Y.B. (2017). Distinct contributions of the thin and thick filaments to length-dependent activation in heart muscle. *Elife* 6.

Zhang, X., Liu, Z.Q., Campbell, K.S., and Wenk, J.F. (2018). Evaluation of a Novel Finite Element Model of Active Contraction in the Heart. *Front Physiol* 9**,** 425.
